# A Ce^4+^ Aluminum Hydride Complex

**DOI:** 10.1021/jacs.6c09952

**Published:** 2026-07-11

**Authors:** Matilda I. Duffy, Nathan R. Loutsch, Benjamin M. Mullen, Hongwei Wu, Bess Vlaisavljevich, Henry S. La Pierre

**Affiliations:** † School of Chemistry and Biochemistry, 1372 Georgia Institute of Technology, Atlanta, Georgia 30332, United States; ‡ Department of Chemistry, 4083 University of Iowa, Iowa City, Iowa 52240, United States; § School of Mechanical Engineering, 1372 Georgia Institute of Technology, Atlanta, Georgia 30332, United States; ∥ Physical Sciences Division, Pacific Northwest National Laboratory, Richland, Washington 99354, United States

## Abstract

Complexes of reducing hydride ligands by high-oxidation state cerium are unknown due to the fundamental mismatch in their redox chemistry. Herein we report the synthesis, characterization, and reactivity of the first example of a Ce^4+^ aluminum hydride complex. Synthetic strategies adapted from the preparation of Ce^4+^ alkyl complexes facilitated the isolation of [Ce^4+^(κ^2^-H_3_AlC­(TMS)_3_)­(NP­(^t^Bu)_3_)_3_] (**CeHAl**). The bonding and structure of this complex is characterized by single-crystal XRD, NMR, and UV–vis–NIR spectroscopy, and DFT computations. The fundamental reactivity profile is evaluated by cyclic voltammetry and small-molecule reactions.

## Graphical abstract

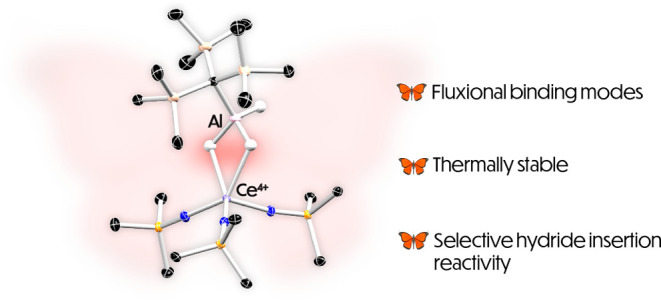

The formation of PuH_*x*_ is a critical technical challenge in the storage of plutonium metal.^1,2^ The direct study of the structure, bonding, and reactivity of this phase is complicated by its pyrophoricity and radiological challenges. Furthermore, accurately determining hydride positions near the heavy metal is complicated by the neutron cross-section of Pu.^3,4^ Molecular complexes can mitigate some of these challenges. Cerium, which can serve as a lanthanide analogue for plutonium due to its similar electrochemical behavior and Shannon ionic radius, was targeted to establish chemical methodology for the preparation of Pu complexes.^5^ Few examples of mononuclear cerium-hydride molecular complexes exist, and there are no crystallographic examples of a Ce^4+^ hydride.^6–18^

Coordination of reducing ligands, like hydrides, to a strongly oxidizing Ce^4+^ ion is difficult to achieve due to its tendency to undergo single-electron reduction to Ce^3+^ (and concomitant Ce–X bond homolysis).^19,20^ Recently, a variety of ligand architectures have been demonstrated to support complexes of oxidizing Ce^4+^ with reducing heteroligands such as Ce^4+^ halide,^21–23^ alkyl,^24,25^ phosphido,^26^ aryl,^25,27,28^ amide,^29^ imido,^30^ oxo,^30–32^ and other^33^ complexes. Herein we report the extension of synthetic strategies used to access Ce^4+^ alkyl complexes^24^ to afford the successful isolation, characterization, and reactivity studies of [Ce^4+^(κ^2^-H_3_AlC­(TMS)_3_)­(NP­(^t^Bu)_3_)_3_], (**CeHAl**) (^t^Bu = *tert*-butyl; TMS = Si­(CH_3_)_3_), the first example of a Ce^4+^ aluminum hydride complex (Figure 1A).

**Figure 1:**
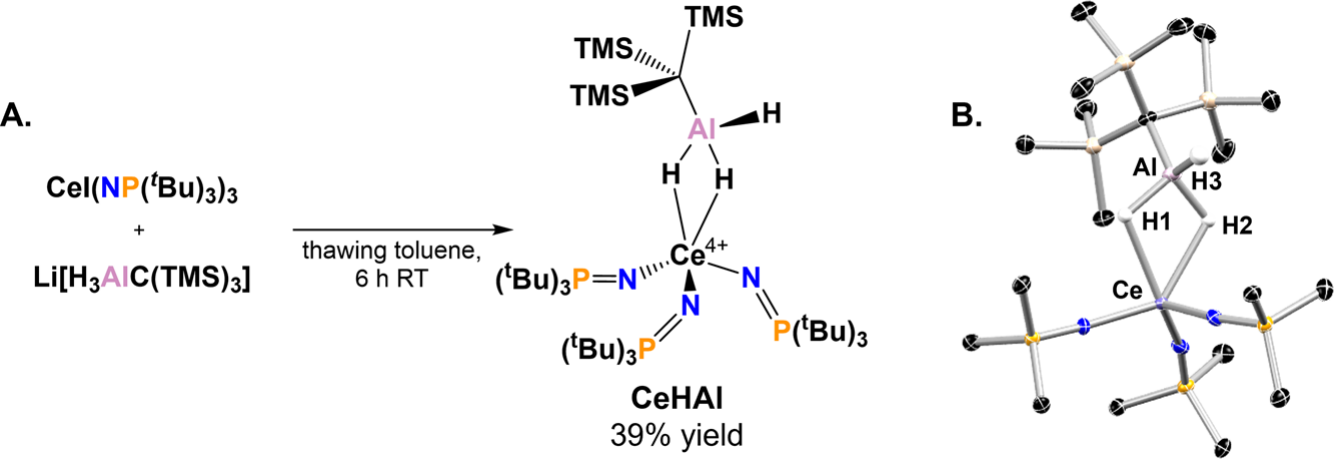
Synthetic scheme for **CeHAl** (A) and truncated molecular structure (B) as determined by SC-XRD with thermal ellipsoids (Al: pink, C: black, Ce: purple, H: white, N: blue, P: orange, Si: beige) shown at 50% probability. All nonhydride H atoms and imidophosphorane −CH_3_ groups have been omitted.

**CeHAl** is synthesized via the addition of 1.5 equivalents of Li­[H_3_AlC­(TMS)_3_][Bibr ref34] to a thawing toluene slurry of [Ce^4+^I­(NP­(^t^Bu)_3_)_3_][Bibr ref24] in a glovebox cold well. After workup, ruby red crystals of **CeHAl** grow at −35 °C in 39% yield. Incomplete conversion of the cerium starting material and the high solubility of the product in conditions in which coproducts remain soluble limit the crystalline yield.

In the solid state, the cerium ion in **CeHAl** is five-coordinate, supported by three imidophosphorane ligands and two hydrides, such that the [H_3_AlC­(TMS)_3_]^1–^ ligand is bound κ^2^ with two of the three hydrides bridging the Al and Ce atoms, and one hydride is terminal on the Al (Figure 1B), which is consistent with extant mononuclear lanthanide aluminum hydrides.^35^ The hydrides were identified in the electron difference map in the single-crystal X-ray diffraction (SC-XRD) molecular model. The average Al–H_bridging_, Al–H_terminal_, and Ce–H bond lengths are 1.61(1), 1.56(1), and 2.46(3) Å, respectively. The average Al–H–Ce angle is 105(2)° and the H–Ce–H angle is 57(1)°. The distance between the Ce and Al is 3.27(3) Å. The average Ce–N and N–P bond lengths and Ce–N–P angles are consistent with other Ce^4+^ systems.^24,36–39^

Solution NMR spectra of **CeHAl** are consistent with the overall connectivity established by SC-XRD with a single resonance observed in ^31^P­{^1^H} NMR at 11.82 ppm and a doublet at 1.41 and singlet at 0.56 ppm in ^1^H NMR corresponding to the ^t^Bu and TMS protons, respectively. However, the solution connectivity of the Al–H–Ce linkage is ambiguous. In the ^1^H­{^27^Al} NMR spectrum, a broad peak at 5.85 ppm is resolved which integrates to approximately 3 protons (Figure 2A). Due to the commensurate relaxation rate of ^27^Al and the magnitude of the ^1^H–^27^Al coupling constant, accurate integration of protons bound to Al requires decoupling the ^27^Al and ^1^H nuclei.^40,41^ The single resolved resonance indicates either that the [H_3_AlC­(TMS)_3_]^1–^ ligand is bound κ^3^ in solution or that the bound and unbound hydrides are rapidly exchanging on the NMR time scale. Variable temperature studies down to −70 °C evince no decoalesence nor change in the peak width (Figure S13). Notably, the compound is thermally stable up to 92 °C in solution, as observed by ^31^P­{^1^H} NMR (Figure S7), and the line width of the 5.85 ppm resonance broadens slightly with heating (Figure S10). Conversely, Fourier-transform infrared spectroscopy (FT-IR) studies (Figure 2B) cannot resolve this ambiguity as two features are observed in the 1800–1500 cm^–1^ region regardless of whether [H_3_AlC­(TMS)_3_]^1–^ is bound κ^2^ or κ^3^ (see the Supporting Information).^42,43^

**Figure 2:**
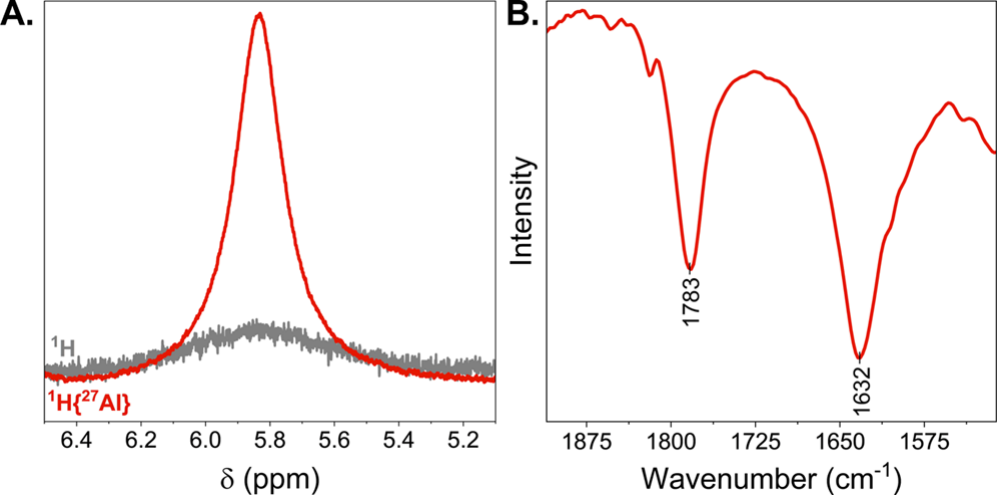
**CeHAl** characterized by (A) ^1^H (gray) and ^1^H­{^27^Al} (red) NMR at 25 °C and (B) solid-state FT-IR.

To further elucidate the solution-state behavior of **CeHAl**, density functional theory (DFT) was used to examine connectivity. DFT geometries converged to both the κ^2^ or κ^3^ coordination modes of [H_3_AlC­(TMS)_3_]^1–^ depending on functional choice (see the Supporting Information). Specifically, the GGA functional PBE converged to κ^3^ species, while use of a hybrid functional or employing a dispersion correction resulted in the κ^2^ species. In other words, the more accurate DFT choices result in good agreement with the solid state (Table S6). Nevertheless, a series of constrained optimizations were employed using CAM-B3LYP-D3 to obtain a best estimate of the energetic difference between the κ^2^- and κ^3^-structures, placing the κ^3^-complex only 2.3 kcal/mol higher in energy (Table S8). Overall, this suggests that the solid-state structure is likely retained in solution, but κ^3^ coordination may be thermally accessible.

To further support the solution structural assignment, DFT computations of the NMR shielding were performed on the κ^2^- and κ^3^-structures. The nuclear shielding constant (σ) is comprised of three components:

1 $$\sigma =\sigma_{\mathrm{dia}}+\sigma_{\mathrm{para}}+\sigma_{\mathrm{SO}}$$

where the contributions are σ_dia_ (diamagnetic), σ_para_ (paramagnetic), and σ_SO_ (spin–orbit). The magnitude of σ_SO_ is used for assessing M–L covalency and correlates to the amount of ligand σ-character and M–L orbital overlap present in the bonds.^44^ The σ_SO_ value can be indirectly obtained from DFT as the difference in σ values from a scalar relativistic (SR) and spin–orbit (SO) calculation. This computed relativistic shift is denoted as Δ_SO_. The chemical shift including spin–orbit coupling (δ_SO_) is directly comparable to experiment. The computations on the κ^2^-structure result in two distinct chemical shifts (δ_SO_ = 8.1, 7.9, 4.90 ppm) which, upon averaging, agree well with experiment (Table S13). Moreover, Δ_SO_ is larger for the κ^2^-H atoms (Table 1). On the other hand, NMR computations for the κ^3^ structure resulted in similar chemical shifts (δ_SO_ = 6.8, 7.0, 6.7 ppm), with a Δ_SO_ of 2.1 ppm. Averaging both structures also provides good agreement with experiment (δ_SO,avg_ = 6.9 ppm), consistent with the assignment of a solution species that may be fluxional.

**Table 1:** ^1^H NMR Chemical Shifts Computed with (SO−)­PBE on **CeHAl** ^a^

^1^H	δ_exp_	δ_SO_	δ_SR_	Δ_SO_
κ^2^-H		8.00	5.04	2.96
H_Term_.		4.90	4.44	0.46
H_avg_	5.85	6.97	4.84	2.12

a The relativistic shift (**Δ**_**SO**_) is the difference between the SR and SO computed values.

The Ce–H–Al natural localized molecular orbital (NLMO) includes 5.7% participation from the Ce center (72% d, 24% f), 25% from the Al center, and 68% from the hydride. On the other hand, for the NLMO along the terminal Al–H bond, the contribution from Al increases significantly to 32%, while Ce only has a minimal contribution at 1.0% (76% d, 17.3% f). The hydride contribution remains large at 67.1% (Figure S62; Table S14).

The UV–vis–NIR spectrum of **CeHAl** has a high energy feature at λ_max_ = 370 nm (ε = 5750 M^–1^ cm^–1^) with a shoulder at approximately 420 nm (Figure S51). Both features are assigned as ligand-to-metal charge transfer (LMCT) bands and are consistent with other Ce^4+^ imidophosphorane complexes.^24,36–39^ Time-dependent density functional theory (TD-DFT) calculations of **CeHAl** support this assignment (Figures S64 and S65). Natural transition orbitals (Table S16) from selected states highlight the LMCT character arising from transitions associated with the main feature at 370 nm as originating from the N 2p orbitals on the imidophosphorane ligands into the Ce 4f orbitals. A molecular orbital diagram (Figure S66) highlights the occupied valence orbitals on the [NP­(^t^Bu)_3_]^1–^ and [H_3_AlC­(TMS)_3_]^1–^ ligands and the empty virtual Ce 4f. Fitting the experimental spectrum reveals two peaks at 3.5 and 2.9 eV (354 and 430 nm, respectively) in the LMCT feature (Figure S53). These features could arise from either a lower separation of the high energy shoulder predicted by TD-DFT at 315 nm in the κ^2^-structure or the experimental observation of an admixture of κ^2^- and κ^3^-structures as the TD-DFT spectrum of the κ^3^- structures evinces a slight blue shift of the N 2p to Ce 4f transition (Figures S64 and S65). Experimental NMR and UV–vis–NIR spectra, in conjunction with DFT, cannot definitively assign whether this complex presents rapid exchange of bridging and terminal Al–H bonds in a fixed κ^2^-structure or whether exchange occurs via the accessibility of equilibrating κ^2^- and κ^3^-species in solution.

Ce L_3_-edge X-ray absorption near edge spectroscopy (XANES) further corroborates the oxidation-state assignment for **CeHAl**. Comparison of the spectrum of **CeHAl** and that of the previously reported homoleptic Ce^3+^ complex Cs­[Ce­(NP^t^Bu_3_)_4_] reveals that the white line energy of **CeHAl** is shifted to higher energy by 1.4 eV (Figure S56).^39^ This shift, in conjunction with the observed double-peak white-line feature of **CeHAl**, is diagnostic of the 4+ oxidation state.^37,38,45–53^

Bonding in **CeHAl** was also investigated via quantum theory of atoms in molecules, bond order analysis, and energy decomposition analysis (EDA). EDA results exploring the interaction of the [H_3_AlC­(TMS)_3_]^1–^ ligand with the positively charged remainder of the molecule demonstrate that the interaction is predominantly electrostatic (63.2%) with non-negligible orbitalic (26.8%) and dispersion (10.0%) contributions (Table S11). The orbitalic contribution can be further decomposed via ETS-NOCV analysis. The deformation densities indicate two pair orbital interactions: a stronger one via the κ^2^-H bonds (−12.7 kcal/mol) and a weaker “through space” interaction (−9.8 kcal/mol) involving the aluminum center (Figure 3, Table S12, Figure S61). These interactions lead to the Δ_SO_ observed via NMR.

**Figure 3:**
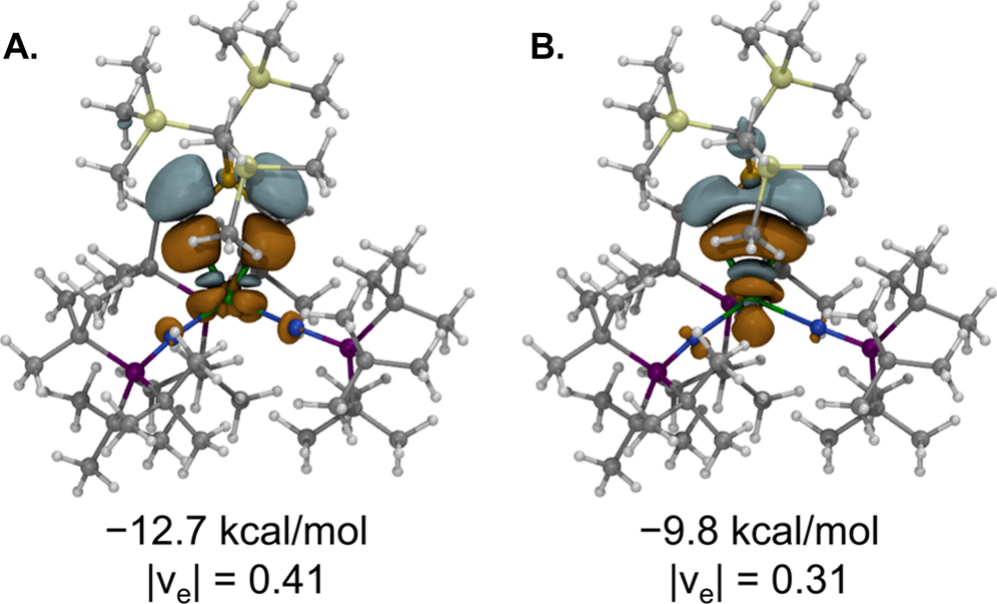
ETS-NOCV pair deformation densities for the Ce–H interaction. The interaction energy and the eigenvalue associated with electrons transferred, |v_e_|, are included. Blue and orange regions represent areas of charge depletion and accumulation, respectively.

The redox properties of **CeHAl** were analyzed via cyclic voltammetry (Figure S44).^19^ The Ce^4+/3+^ redox couple has an *E*_1/2_ value of −2.01 V vs Fc^+/0^, which is consistent with similar systems.^24^ There is a small secondary feature around −1.9 V vs Fc^+/0^ which is associated with the Ce^4+/3+^ couple and is likely a structural rearrangement, which has been previously observed.^24,36,38,54^ Even though the Δ*E*_p_ is significantly large, indicating that this is not an electrochemically reversible system, the single oxidative feature for **CeHAl** supports the notion that chemical reversibility could be accessible via synthetic routes.

However, testing this possibility reveals more complex behavior with chemical reductants, and no anionic Ce^3+^ structural analog of **CeHAl** can be identified. Reduction of **CeHAl** using alkali metals (KC_8_ and NaHg amalgam) yields a mixture of products by *in situ* NMR (Figures S15–S20). One of the reduction products was identified as a rearranged Ce^3+^ product, [Ce^3+^(NP­(^t^Bu)_3_)_2_((NP­(^t^Bu)_3_)_2_AlH_2_)], by SC-XRD analysis of a single crystal (bulk isolation is challenging on the requisite small reaction scales). Reduction with decamethylcobaltocene leads to a majority product as determined by *in situ* NMR, however this product rapidly decays (∼2 h at RT in *d*_8_-THF) and by ^31^P­{^1^H} NMR indicates the formation of a complex mixture of products (Figures S21 and S22).

**CeHAl** is relatively inert in the presence of neutral Lewis bases 4-dimethylaminopyridine and triphenylphosphine oxide, even when heated to 80 °C (Figures S23 and S24). Additionally, no reaction was observed when **CeHAl** was exposed to O_2_, H_2_, *tert*-butyl isocyanide, *N,N’*-dicyclohexylcarbodiimide (as CO and CO_2_ analogues, respectively), and benzhydrol at short reaction times (0.1 to 4 h) at room temperature (longer reaction times and heating sometimes led to complex mixtures) (Figures S25–S31). (C_6_F_5_)_3_B­(H_2_O), as a solid source of air-free water, produces H_2_ gas and leads to partial decomposition of **CeHAl** (*in situ* NMR, Figures S32 and S33).^55^

Ce^3+^ hydrides are invoked in the reduction of unsaturated α,β-ketones to allylic alcohols in the Luche reduction.^56–58^ Therefore, simple ketones were considered as a substrate for reduction. Treatment of **CeHAl** with one equivalent of benzophenone results in exclusive hydride insertion into the ketone (*in situ* ^1^H and ^31^P­{^1^H} NMR, Figures S34–S37, yield limited by further reactions of the alane with benzophenone). The identity of this insertion product, [Ce^4+^(OCHPh_2_)­(NP­(^t^Bu)_3_)_3_], (**CeOPh**_**2**_), (Ph = C_6_H_5_), was independently established on preparative scale via a salt metathesis reaction of [Ce^4+^I­(NP­(^t^Bu)_3_)_3_] and K­[OCHPh_2_] (Figure 4A, Route 2).

**Figure 4:**
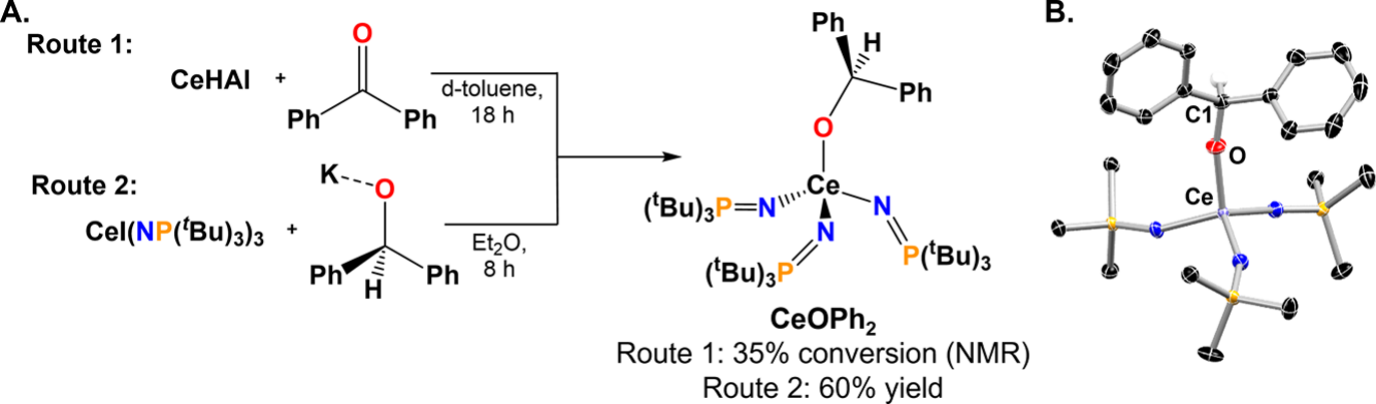
Synthetic routes for **CeOPh**_**2**_ (A) and truncated molecular structure (B) as determined by SC-XRD with thermal ellipsoids (Ce: purple, C: black, H: white, N: blue, O: red, P: orange) shown at 50% probability. Imidophosphorane −CH_3_ groups and all hydrogen atoms except the one that originates from a hydride source (Route 1) have been omitted.

In sum, we report the isolation and characterization of the first Ce^4+^ aluminum hydride. This synthetic methodology and established stability to homolytic reduction provides a foundation for the study of plutonium hydride complexes. **CeHAl** adds to a growing family of heteroleptic Ce^4+^ complexes that overcome redox incompatibility via ligand design. The thermal stability of **CeHAl** facilitates selective hydride insertion reactivity which opens novel, redox-neutral chemical pathways at Ce^4+^.

## Supplementary Material



## Data Availability

Input and output files and xyz structures available: https://doi.org/10.6084/m9.figshare.32245104.

## Notes

Experimental details, NMR, infrared, XAS, and UV–vis–NIR spectra, molar absorptivity, electrochemical analysis, XANES, crystallographic data, and computational details (PDF)
