# Supplementary material for: A Ce4+ Aluminum Hydride Complex
Source: J Am Chem Soc. 2026 Jul 11;148(28):29649–54. doi: 10.1021/jacs.6c09952 (PMC13397886; doi:10.1021/jacs.6c09952)
Supplement: Supplementary file 1 [file ja6c09952_si_001.pdf]

Supporting Information for:

A Ce^{4+} Aluminum Hydride Complex

Matilda I. Duffy†, Nathan R. Loutsch‡, Benjamin M. Mullen‡, Hongwei Wu†, Bess Vlaisavljevich‡, Henry S. La Pierre*, †, ^, §

†School of Chemistry and Biochemistry, Georgia Institute of Technology, Atlanta, Georgia, 30332 United States
‡Department of Chemistry, University of Iowa, Iowa City, Iowa, 52240 United States
^School of Mechanical Engineering, Georgia Institute of Technology, Atlanta, Georgia, 30332 United States
§Physical Sciences Division, Pacific Northwest National Laboratory, Richland, Washington, 99354 United States

Corresponding author e-mail: hsl@gatech.edu

Table of Contents

TABLE OF CONTENTS **S1**

GENERAL CONSIDERATIONS **S2**

SYNTHETIC METHODS **S4**

NMR SPECTROSCOPY **S6**

- **Li[$H_3AlC(TMS)_3$]** S6
- **CeHAl** S7
- Thermal Stability of **CeHAl** by Variable Temperature NMR S9
- **CeHAl** and Alkali Metal Reductions S12
- Reduction of **CeHAl** by $CoCp_2$* S14
- **CeHAl** and 4-dimethylaminopyridine S15
- **CeHAl** and triphenylphospine oxide S16
- **CeHAl** and O_2 .... S17
- **CeHAl** and tertbutyl isocyanide S18
- **CeHAl** and N,N'-dicyclohexylcarbodiimide S19
- **CeHAl** and H_2 .... S20
- **CeHAl** and benzhydrol S22
- **CeHAl** and $(C_6F_5)_3B(H_2O)$ S23
- **CeHAl** and benzophenone S24
- **CeOPh₂** S25

ELECTROCHEMISTRY **S29**

INFRARED SPECTROSCOPY **S31**

UV-VIS-NIR ELECTRONIC ABSORPTION SPECTRA **S33**

X-RAY ABSORPTION NEAR-EDGE SPECTROSCOPY **S35**

SINGLE CRYSTAL X-RAY DIFFRACTION **S37**

COMPUTATIONAL DETAILS **S41**

REFERENCES **S60**

General Considerations

Unless otherwise noted, all reagents were obtained from commercial suppliers. Manipulations and syntheses were conducted using Schlenk line techniques under Ar or in an N_2 filled glovebox (Vigor, <0.1 ppm O_2/H_2O). All glassware was dried at ca. 160 °C for >8 hours. Celite and molecular sieves (3 Å) were dried under vacuum at >250 °C for more than 24 hours. Pentane and toluene were purged with UHP Ar gas (Airgas) and dried through the columns of a commercial solvent purification system (JC Meyer Solvent Systems) and stored over 3 Å molecular sieves in the glovebox. C_6D_6 was dried over 3 Å molecular sieves and vacuum transferred prior to use. Deuterated toluene was freeze-pump-thawed and dried over 3 Å molecular sieves. Decamethylcobaltocene was recrystallized from pentane the day before use. Lithium aluminum hydride was purified by filtration over celite and a medium frit in toluene. $HOCHPh_2$ was sublimed under vacuum (~5x10^{-3} mmHg) at 45 °C for three days to yield pure crystalline material. The following were prepared by previously reported methods: $CeI_3(THF)_4$,[1] $HNP(^tBu)_3$,[2] $CeI(NP(^tBu)_3)_3$,[2] $X[H_3AlC(TMS)_3]$ ($X = Li^+, K^+$),[3,4] KC_8,[5] $(C_6F_5)_3B(H_2O)$,[6] $KOCHPh_2$.[7] Reactions done with gases (O_2 and H_2) were carried out in an air and moisture free glovebox. The gas canister, regulator, rubber tubing, and a balloon were brought into the glovebox. The analyte was added to a J-Young tube in deuterated solvent, then a slight vacuum was pulled on the tube. The balloon was filled with a small amount of gas (such that the balloon fits in the palm of a hand) and was quickly placed onto the J-Young tube via an adapter fitted to a standard J-Young cap. The J-Young tap was then opened, and the analyte was exposed to the gas for 5-10 minutes. The tap was closed and the balloon was removed such that the J-Young remained a closed system.

Elemental analyses on C, H, N were collected at the University of Iowa Facility (Iowa City, IA), on an Exeter Analytical CE-440 elemental analyzer at the University of Iowa MATFab Facility.

NMR spectra were obtained on a Bruker Avance III 400 MHz or 500 MHz spectrometer at 298 K, for all compounds unless otherwise noted. ^1H NMR spectra, $^1H\{^{27}Al\}$ spectra, and $^{13}C\{^1H\}$ spectra are referenced to the residual ^1H resonances of the deuterated solvent unless otherwise noted. $^{31}P\{^1H\}$ NMR spectra are referenced using an absolute reference to H_3PO_4. $^{27}Al\{^1H\}$ NMR spectra are referenced using an absolute reference to $AlNO_3$. Peak position is reported, followed by peak multiplicity, integration value, and assignment where applicable. Abbreviations for the peak multiplicity are as follows: s (singlet); d (doublet); t (triplet); m (multiplet), b (broad).

$^1H\{^{27}Al\}$ were collected using the Bruker zgig sequence with inverse gated decoupling, and the broadband decoupling sequence GARP4 (Globally Optimized Alternating-Phase Rectangular Pulses) was employed with about 3125 Hz field strength during acquisition time and the decoupler offset was set accordingly from the analyte's ^{27}Al shift.

For **electrochemical analysis,** $[^nBu_4N][BPh_4]$ was prepared as previously reported[8] and dried under vacuum at 85 °C for 24 h prior to use. Ferrocene and decamethylferrocene were sublimed before use. Electrochemical data were measured using a Pine WaveDriver 20 Bipotentiostat/Galvanostat. Measurements were performed in a glovebox under an atmosphere of N_2 with a glassy carbon working electrode (3 mm diameter), a bare Ag wire reference electrode in a fritted capillary filled with the corresponding electrolyte solution, and a platinum wire counter electrode at ambient temperature (~25 °C, 298 K). The fritted capillary was stored in electrolyte

solution when not in use, and the inner solution was replaced with fresh electrolyte solution prior to use. The glassy carbon electrode was polished before use, per established methods.[9] Electrolyte solutions were prepared in THF (**CeHAl**) with 0.05 M [$^{n}Bu_4N$][BPh_4] electrolyte. Measurements were made in positive feedback iR compensation mode (~620 Ω). Voltammograms were internally referenced by adding a small amount of ferrocene and decamethylferrocene at the end of the experiment. Ferrocene and decamethyferrocene were also used as external standards, taken before and after the experiment. All potentials are reported vs. Fc^{+}/Fc^{0} based on an external reference, the $Fc^{+/0}$ shift from before and after the experiment were averaged to account for electrode drift.

Infrared (IR) spectroscopy was conducted on a Bruker ALPHA FTIR Spectrometer from 400 to 4000 cm^{-1}, using an ATR attachment inside of a N_2 glovebox. The intensities of the peaks are reported using the following abbreviations: w (weak); medium (medium); s (strong); vs (very strong); br (broad).

UV-vis NIR spectroscopy was conducted on a Hitachi UH4150 UV-vis-NIR scanning spectrophotometer from 2200 nm to 240 nm. Characterization was performed in small-volume screw cap quartz cuvettes (Starna Scientific) with a 1 cm path length.

X-Ray Absorption Near-Edge Spectroscopy (XANES) spectra were collected at Stanford Synchrotron radiation Lightsource (SSRL) in Menlo Park, CA at beamlines 7-3 (**CeHAl**). All data reported are cerium L_3-edge transmission XANES. Data for $CeI(NP(^{t}Bu)_3)_3$, $CeBn(NP(^{t}Bu)_3)_3$ (Bn = CH_2Ph), $CeNpt(NP(^{t}Bu)_3)_3$ (Npt = $CH_2C(CH_3)_3$), and $CsCe(NP(^{t}Bu)_3)_4$ were previously reported.[10,11] Crystalline samples of the material and boron nitride (BN) (dried under vacuum at >250 °C for 24 hours before use) were shipped to the beamline in flame-sealed glass ampoules. Inside of an argon glovebox, a mixture of analyte and ~100x BN (by moles) were ground to a fine powder using a mortar and pestle. Sample holders were a 1/32" thick aluminum plate with a 3 x 15 mm^2 window in the center. One side of the plate was covered with 0.5 mil Kapton tape. The sample was loaded onto the sticky side of the Kapton tape then gently pressed into uniform thickness using the back of a spatula. The sample was sealed with a second piece of 0.5 mil Kapton tape. The aluminum holder was screwed into a sample holder provided by SSRL. The sample was removed from the glovebox and immediately submerged in liquid nitrogen to be transported to the beamline hutch. At the hutch, the sample holder was attached to the sample rod, while remaining submerged in liquid nitrogen. The sample was immediately inserted into the cryostat once fastened to the rod. The cryostat was cycled 3-5 times. Data was calibrated to the energy of the first inflection point of the K-edge of chromium foil (5989 eV). Data was collected in triplicate, then aligned, calibrated, normalized, and averaged using Athena.

Single Crystal X-Ray Diffraction (SC-XRD) experiments were performed at Georgia Institute of Technology X-ray Crystallography Facility on a Bruker D8 VENTURE dual wavelength Mo/Cu three-circle diffractometer with a microfocus sealed X-ray tube using a mirror optics as monochromator and a Bruker PHOTON III detector. The diffractometer was equipped with an Oxford Cryostream 800 low temperature device and used MoK_{α} radiation (λ = 0.71073 Å). All data were integrated with SAINT V8.40B and a multi-scan absorption correction using SADABS 2016/2 was applied.[12,13] The structure was solved by direct or dual methods with SHELXT and refined by full-matrix least-squares methods against F^2 using SHELXL 2018/3.[14,15] All non-hydrogen atoms were refined with anisotropic displacement parameters. All C-bound hydrogen

atoms were refined isotropic on calculated positions using a riding model with their U_{iso} values constrained to 1.5 times the U_{eq} of their pivot atoms for terminal sp^3 carbon atoms and 1.2 times for all other carbon atoms. Disordered moieties were refined using bond lengths restraints and displacement parameter restraints. All tables and the final CIF file were generated using FinalCif.[16]

Synthetic Methods

[Ce(κ^2-H_3AlC(TMS)$_3$)(NP(^{t}Bu)$_3$)$_3$], CeHAl: In a glovebox, all materials were chilled in a cold well prior to use. In the cold well, 0.7 mL toluene and a glass stir bar were added to CeI(NP(^{t}Bu)$_3$)$_3$ (1 equiv., 87.2 mg, 0.095 mmol). Li[H_3AlC(TMS)$_3$]·(THF) (1.5 equiv., 48.71 mg, 0.143 mmol) was transferred to the vial with the cerium solution as a slurry in 1 mL aliquots of toluene (to ensure complete transfer) until the total reaction volume was ~ 8 mL. The reaction vessel was swirled by hand for 90 seconds in the cold well, then the ruby red solution was placed on a stir plate at room temperature and the reaction mixture was stirred for ~12 h. The volatiles were removed *in vacuo* and the crude solid was triturated three times with 1 mL pentane. The residue was then extracted using a minimum amount of cold (-35 °C) pentane and was filtered over celite and glass fiber filter paper into a 1 dram shell vial. The volatiles were removed *in vacuo* and the red residue was taken up in toluene and concentrated to ~1.5 mL for crystallization. Silicone grease was added to an outer 20 mL vial, and the vial was placed in a -35 °C freezer for 8 days. A small crystal was observed after 4 days but was noted to continue to grow. The single ruby red crystal was mechanically separated from solid impurities on the vial walls using a spatula to yield single, spectroscopically pure, large crystal of the title compound (39 mg, 39% yield). Smaller crystals suitable for SC-XRD analysis can be prepared by recrystallization from pentane.
^{1}H{^{27}Al} NMR (400 MHz, toluene-d_8) δ 5.85(b, 3H), 1.41 (d, J = 12 Hz, 81H, ^{t}Bu), 0.56 (s, 27H, TMS).
^{13}C{^{1}H} NMR (101 MHz, C_7D_8) δ 41.89 (d, ^{t}Bu <u>C</u>CH_3), 30.26 (^{t}Bu C<u>C</u>H_3), 5.23 (TMS C<u>C</u>H_3).
^{31}P{^{1}H} NMR (162 MHz, C_7D_8) δ 11.82.
^{27}Al{^{1}H} NMR (104 MHz, C_7D_8) δ 135.68.
IR: 2899 (m), 1780 (w), 1633 (w), 1468 (m), 1386 (w), 1244 (m), 1182 (w), 1032 (s), 933 (m), 864 (s), 796 (s), 728 (m), 660 (m), 617 (s), 492 (m).
Elemental analysis % found(calculated) for: C 51.05(52.59), H 10.28(10.65), N 3.83(4.00). Elemental analysis was repeated with three separate samples, all with similar found % values as is reported. The low % C is consistent with incomplete combustion of the material.

CeHAl + KC_8, NMR Scale: In a glovebox, **CeHAl** (1 equiv., 11.4 mg, 0.011 mmol) was taken up in approximately 0.5 mL toluene-d_8. This solution was added to another vial containing KC_8 (1 equiv, 1.5 mg, 0.011 mmol). The solution was mixed by pipette several times, then added to an NMR tube for immediate analysis by NMR. See Figures S17-S18.

CeHAl + NaHg: In a glovebox, **CeHAl** (1 equiv., 21.8 mg, 0.021 mmol) was added to a vial containing a glass stir bar and NaHg (5% Na) (1 equiv. Na, 9.7 mg amalgam) using 3 mL of THF. The reaction mixture was stirred for ~12 hours. The volatiles were removed *in vacuo* and the crude solid was triturated two times with 1 mL pentane. The residue was then extracted using pentane and filtered over celite and glass fiber filter paper into a 1 dram shell vial. After only crystals of **CeHAl** and Ce(NP(^{t}Bu)$_3$)$_2$((NP(^{t}Bu)$_3$)$_2$AlH_2) formed under various crystallization conditions, volatiles were removed *in vacuo* and the residue was taken up in toluene-d_8 and analyzed via NMR (Figures S19-S20).

CeHAl + DmCc, NMR Scale: In a glovebox, **CeHAl** (1 equiv, 9.2 mg, 0.009 mmol) was taken up in approximately 0.5 mL d_8-THF. This solution was added to another vial containing freshly crystallized and dried decamethylcobaltocene (1 equiv, 2.9 mg, 0.009 mmol). The solution was mixed by pipette several times, then added to an NMR tube for immediate analysis by NMR. See Figures S21-S22.

CeHAl + DMAP, NMR Scale: In a glovebox, **CeHAl** (1 equiv., 7.1 mg, 0.007 mmol) was taken up in approximately 0.5 mL toluene-d_8. This solution was added to another vial containing 4-dimethylaminopyridine (DMAP) (1 equiv, 0.95 mg, 0.007 mmol). The solution was mixed by pipette several times, then added to a J-Young tube for immediate analysis by NMR. The tube was then heated in an oil bath inside of a fume hood to 50 °C for 3 hours, then heated again to 80 °C for 8 hours. See Figure S23.

CeHAl + $OPPh_3$, NMR Scale: In a glovebox, **CeHAl** (1 equiv., 5.2 mg, 0.005 mmol) was taken up in approximately 0.5 mL toluene-d_8. This solution was added to another vial containing triphenylphosphine oxide ($OPPh_3$) (1 equiv, 1.58 mg, 0.005 mmol). The solution was mixed by pipette several times, then added to a J-Young tube for immediate analysis by NMR. The tube was then heated in an oil bath inside of a fume hood to 50 °C for 3 hours, then heated again to 80 °C for 8 hours. See Figure S24.

CeHAl + *tert*-butyl isocyanide, NMR Scale: In a glovebox, **CeHAl** (1 equiv., 7.3 mg, 0.007 mmol) was taken up in approximately 0.5 mL toluene-d_8. This solution was added to another vial containing tertbutyl isocyanide (2.2 equiv, 1.3 mg, 0.015 mmol). The solution was mixed by pipette several times, then added to a J-Young tube. The tube was heated in an oil bath inside of a fume hood to 50 °C for 4 hours, then heated again to 75 °C for 22 hours. See Figure S26.

CeHAl + N,N'-dicyclohexylcarbodiimide, NMR Scale: In a glovebox, **CeHAl** (1 equiv., 5.7 mg, 0.005 mmol) was taken up in approximately 0.5 mL toluene-d_8. This solution was added to another vial containing N,N'-dicyclohexylcarbodiimide (1.25 equiv, 1.4 mg, 0.007 mmol). The solution was mixed by pipette several times, then added to a J-Young tube. The tube was heated in an oil bath inside of a fume hood to 50 °C for 4 hours, then heated again to 75 °C for 22 hours. See Figure S27.

CeHAl + $(C_6F_5)_3B(H_2O)$, NMR Scale: In a glovebox, **CeHAl** (1 equiv., 7.5 mg, 0.007 mmol) was taken up in approximately 0.5 mL toluene-d_8. This solution was added to another vial containing $(C_6F_5)_3B(H_2O)$ (1 equiv, 3.7 mg, 0.007 mmol). The solution was mixed by pipette several times, then added to a J-Young tube. Between NMR interrogations, the sample was stored inside the glovebox. See Figure S32-S33.

$Ce(OCHPh_2)(NP(^tBu)_3)_3$, $CeOPh_2$, Route A: In a glovebox, **CeHAl** (1 equiv., 6.3 mg, 0.006 mmol) was taken up in ~0.5 mL toluene-d_8. This solution was added to a vial containing benzophenone (1 equiv., 1.3 mg, 0.006 mmol). The mixture was mixed by pipette several times, then transferred to a J-Young tube. NMR was taken immediately (~10 minutes after mixing) and again after 18 hours. Between NMR interrogations, the sample was stored inside the glovebox.

After 18 hours, 54% **CeHAl** remained and there was 37% conversion to **CeOPh₂** by $^{31}P\{^1H\}$. The other 9% of the solution was an unidentified co-product at 73.9 ppm (See Figure S34-S37).

Ce(OCHPh₂)(NP(ᵗBu)₃)₃, CeOPh₂, Route B: In a glovebox, 1.0 mL Et_2O and a glass stir bar were added to $CeI(NP(^tBu)_3)_3$ (1 equiv., 54.3 mg, 0.059 mmol). $KOCHPh_2$ (1.1 equiv., 15.4 mg, 0.066 mmol) was added to the cerium solution in 0.5-1 mL Et_2O increments until the total reaction volume reached 4.5 mL Et_2O. The reaction was stirred overnight to yield a cloudy bright orange reaction mixture. The volatiles were removed *in vacuo* and the crude solid was triturated three times with 1 mL pentane. The crude residue was taken up using minimum amount of cold (-35 °C) pentane and filtered over celite and glass fiber filter paper into a 1 dram shell vial. The orange solution was concentrated to ~0.5 mL for crystallization at -35 °C overnight to yield orange crystalline plates (49.4 mg, 85% yield) of the title compound.
^1H NMR (400 MHz, C_6D_6) δ 7.88 (d, J = 8 Hz, 4H), 7.32 (t, J = 6 Hz, 4H), 7.14 (t, J = 8 Hz, 2H), 6.87 (s, 1H, OC*H*), 1.43 (d, J = 12 Hz, 81H, ᵗBu). See Figure S38 for aromatic assignments.
$^{13}C\{^1H\}$ NMR (101 MHz, C_6D_6) δ 151.43, 128.06, 127.30, 125.97, 86.54 (O*C*H), 41.49 (d, ᵗBu *C*CH_3), 30.28 (ᵗBu C*C*H_3). See Figures S39, S40, and S42 for aromatic assignments.
$^{31}P\{^1H\}$ NMR (162 MHz, C_6D_6) δ 14.48.
IR: 2994 (m), 2959 (m), 2897 (m), 2867 (m), 1482 (m), 1471 (m), 1445 (m), 1385 (m), 1362 (m), 1259 (w), 1183 (m), 1056 (vs), 934 (m), 805 (m), 755 (m), 728 (m), 698 (m), 671 (w), 613 (s), 529 (m), 494 (m), 467 (m), 430 (m).
Elemental analysis % found(calculated) for: C 60.18(60.51), H 9.77(9.56), N 3.96(4.32).

NMR Spectroscopy

Li[H₃AlC(TMS)₃]

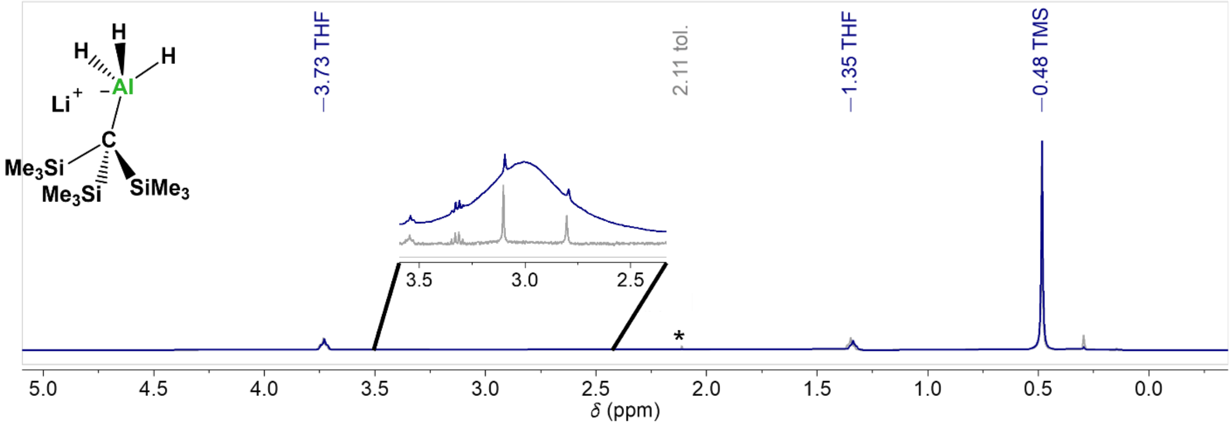

Figure S1: $^1H\{^{27}Al\}$ NMR (blue trace) overlaid with ^1H NMR (gray trace) of **Li[H₃AlC(TMS)₃](THF)** in C_6D_6. Terminal protons on aluminum can be spectroscopically silent due to coupling between the proton and aluminum nuclei. The three hydrides in **Li[H₃AlC(TMS)₃](THF)** are not visible in a standard ^1H NMR (gray trace) but can be found at 3.00 ppm by applying $^1H\{^{27}Al\}$ NMR (blue trace). Residual toluene is denoted in the figure with *. Integration of the proton spectrum was used to determine the number of THF molecules coordinated to the complex.

CeHAl

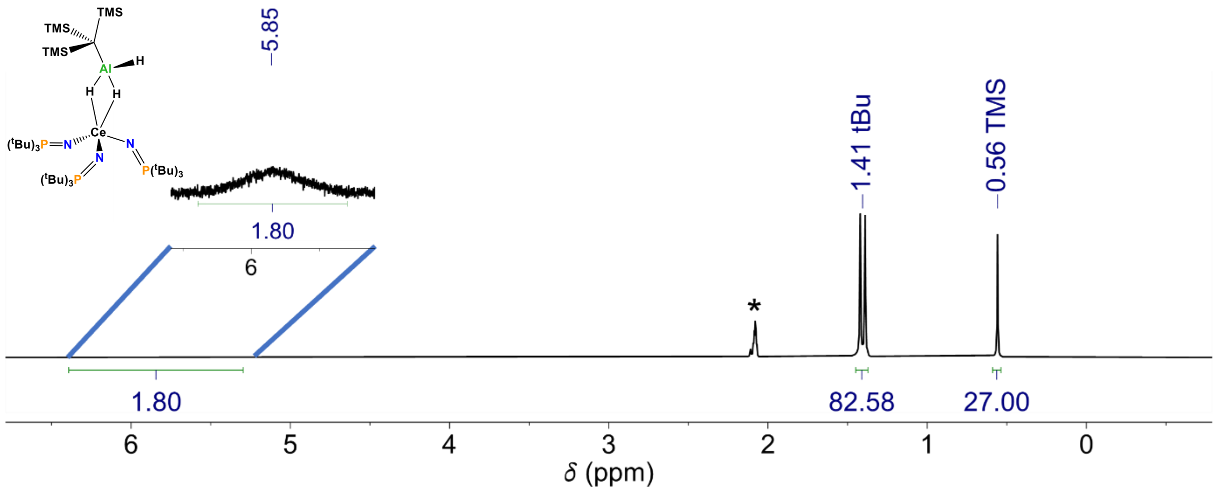

Figure S2: ^{1}H NMR of **CeHAl** in toluene-d$_8$. Residual solvent is denoted in the figure with *.

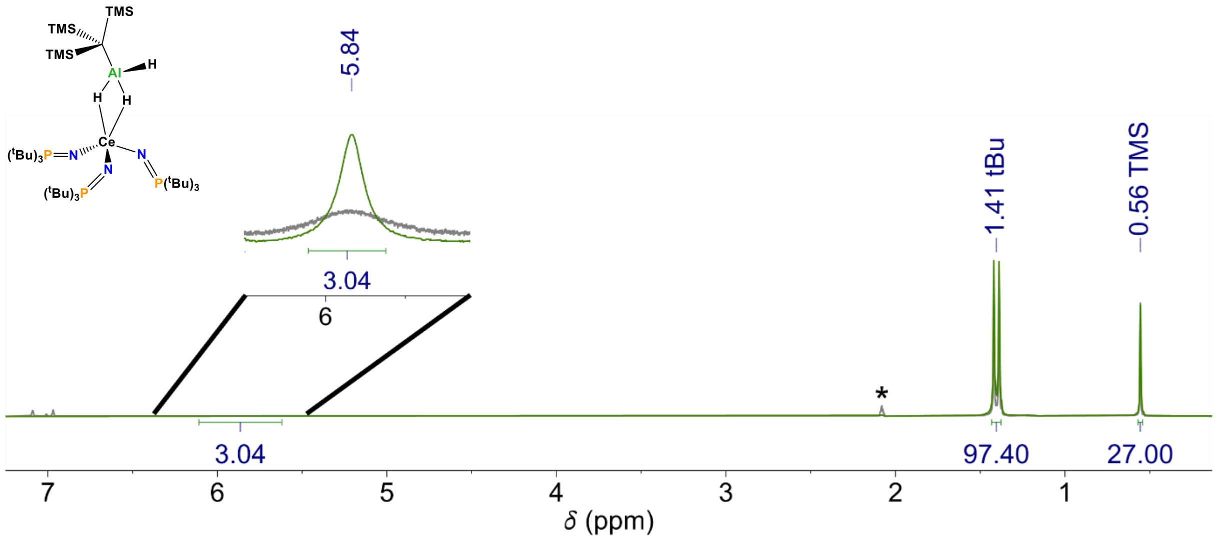

Figure S3: ^{1}H{^{27}Al} NMR (green trace) overlaid with ^{1}H NMR (gray trace) of **CeHAl** in toluene-d$_8$. The 3H signal 5.85 ppm demonstrates the effect of decoupling from the Al nucleus. Residual solvent is denoted in the figure with *.

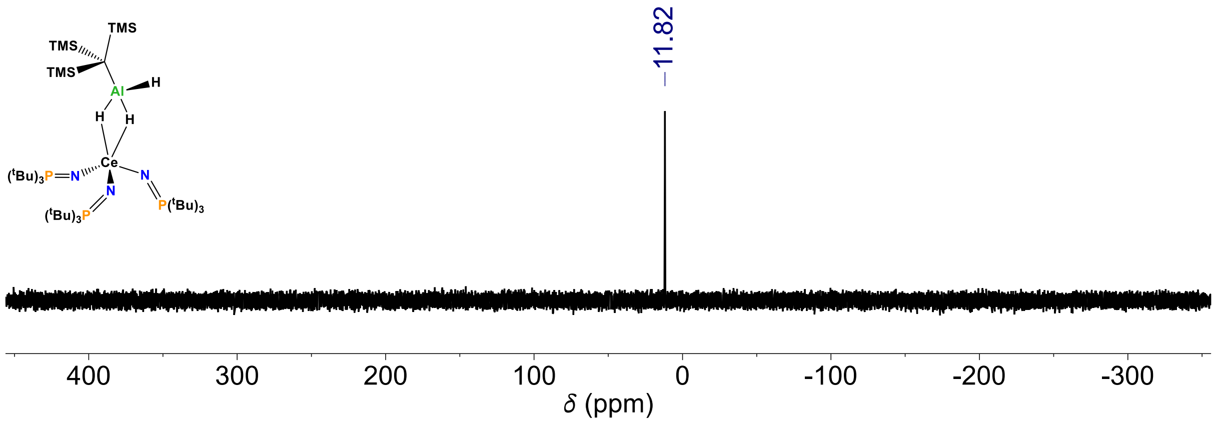

Figure S4: $^{31}P\{^1H\}$ NMR of **CeHAl** in toluene-d$_8$.

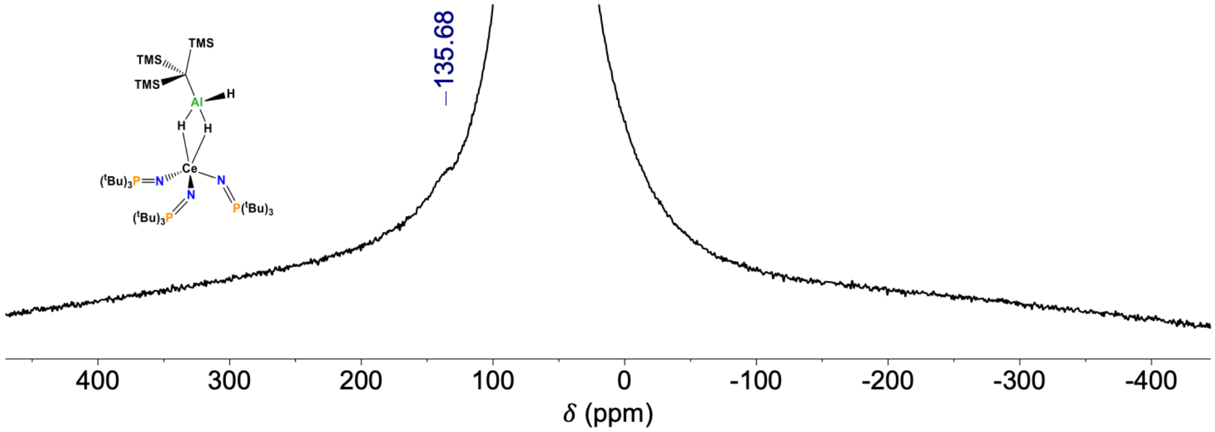

Figure S5: $^{27}Al\{^1H\}$ NMR of **CeHAl** in toluene-d$_8$. The large signal from approximately 100 to -20 ppm is an instrument feature.

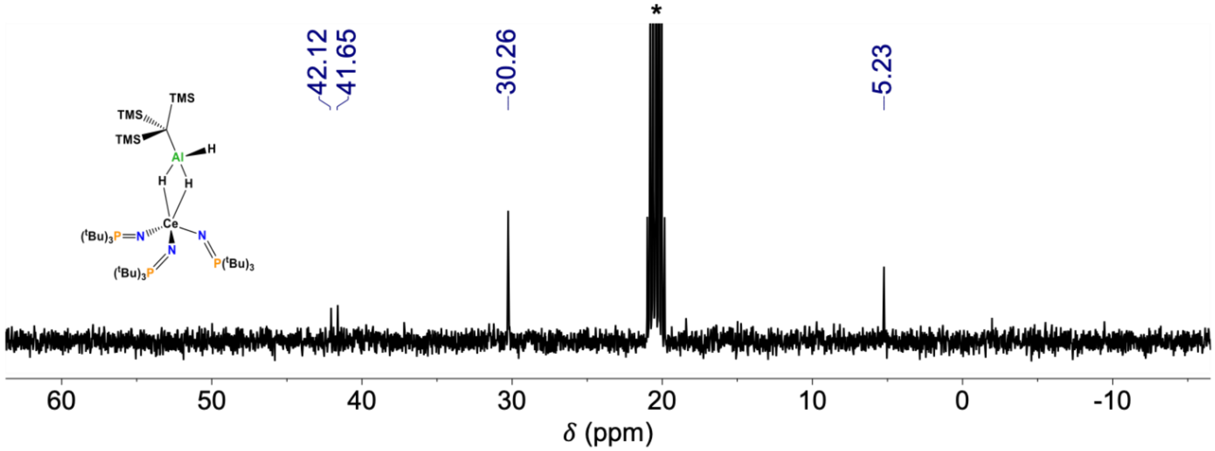

Figure S6: $^{13}C\{^1H\}$ NMR of **CeHAl** in toluene-d$_8$. The ^{13}C resonances of toluene-d$_8$ are noted in the figure as *.

*Thermal Stability of **CeHAl** by Variable Temperature NMR*

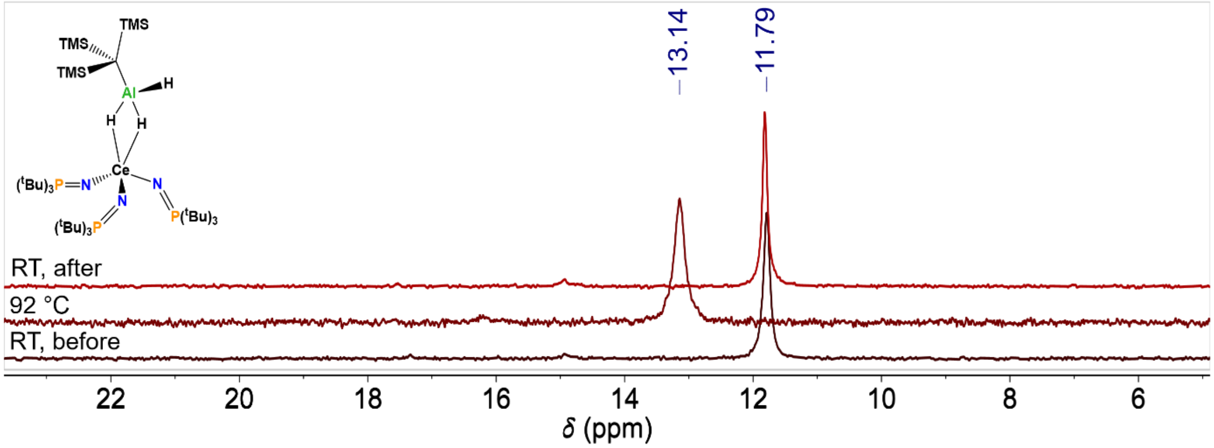

Figure S7: Variable temperature $^{31}P\{^{1}H\}$ NMR of **CeHAl** in toluene-d8 at room temperature before heating, at 92 °C, and room temperature after heating.

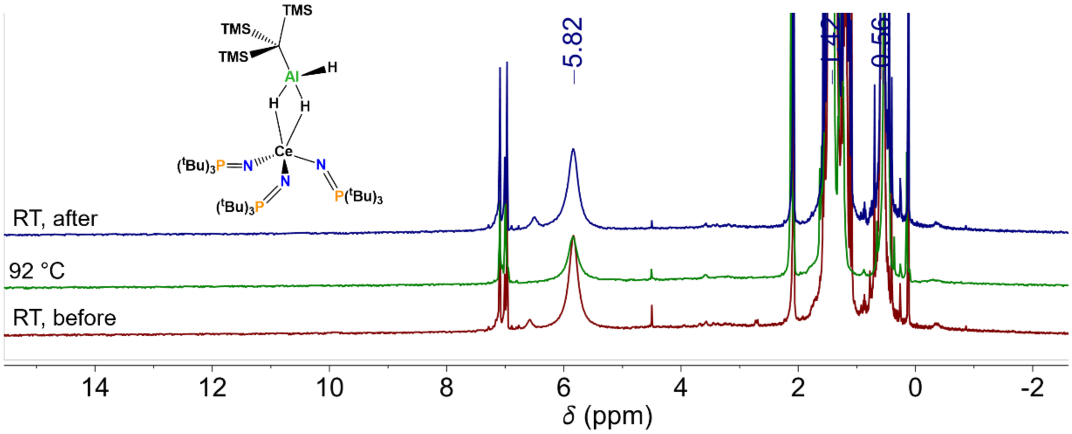

Figure S8: Variable temperature $^{1}H\{^{27}Al\}$ NMR of **CeHAl** in toluene-d8 at room temperature before heating, at 92 °C, and room temperature after heating. No significant change was observed after heating. The region between 3.0 and -0.5 ppm is shown in **Figure S9**.

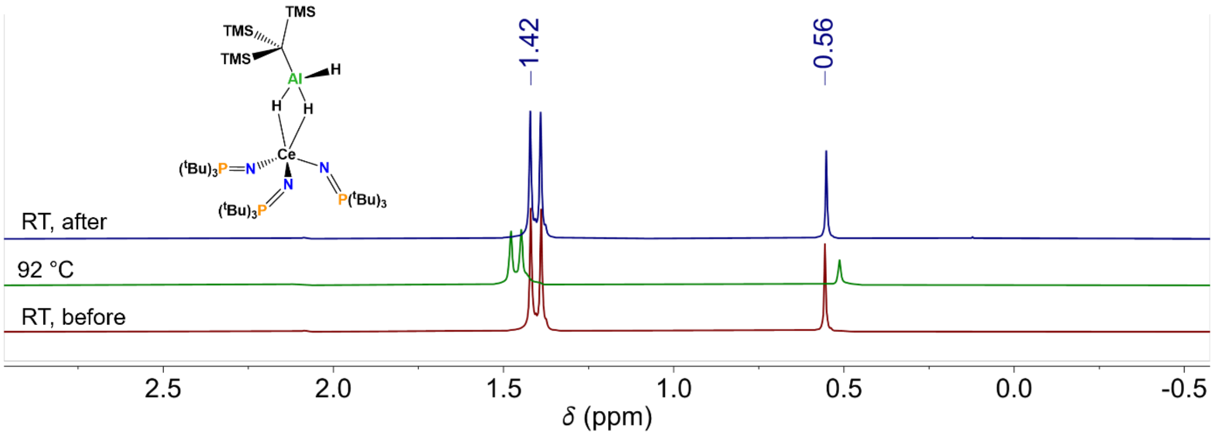

Figure S9: Variable temperature $^{1}H\{^{27}Al\}$ NMR of **CeHAl** in toluene-d₈ at room temperature between 3 and -0.5 ppm before heating, at 92 °C, and room temperature after heating. No significant change was observed after heating.

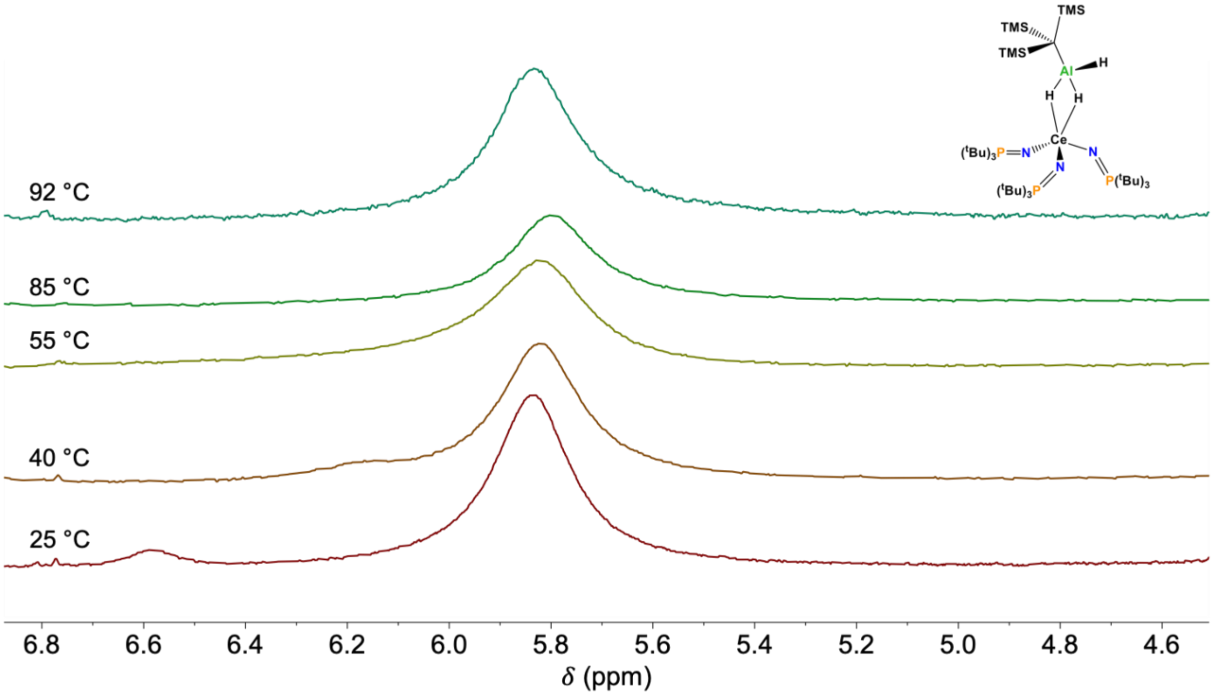

Figure S10: Variable temperature $^{1}H\{^{27}Al\}$ NMR of the hydride peak in **CeHAl** in toluene-d_8. The line width of the peak is observed to broaden slightly as the temperature increases.

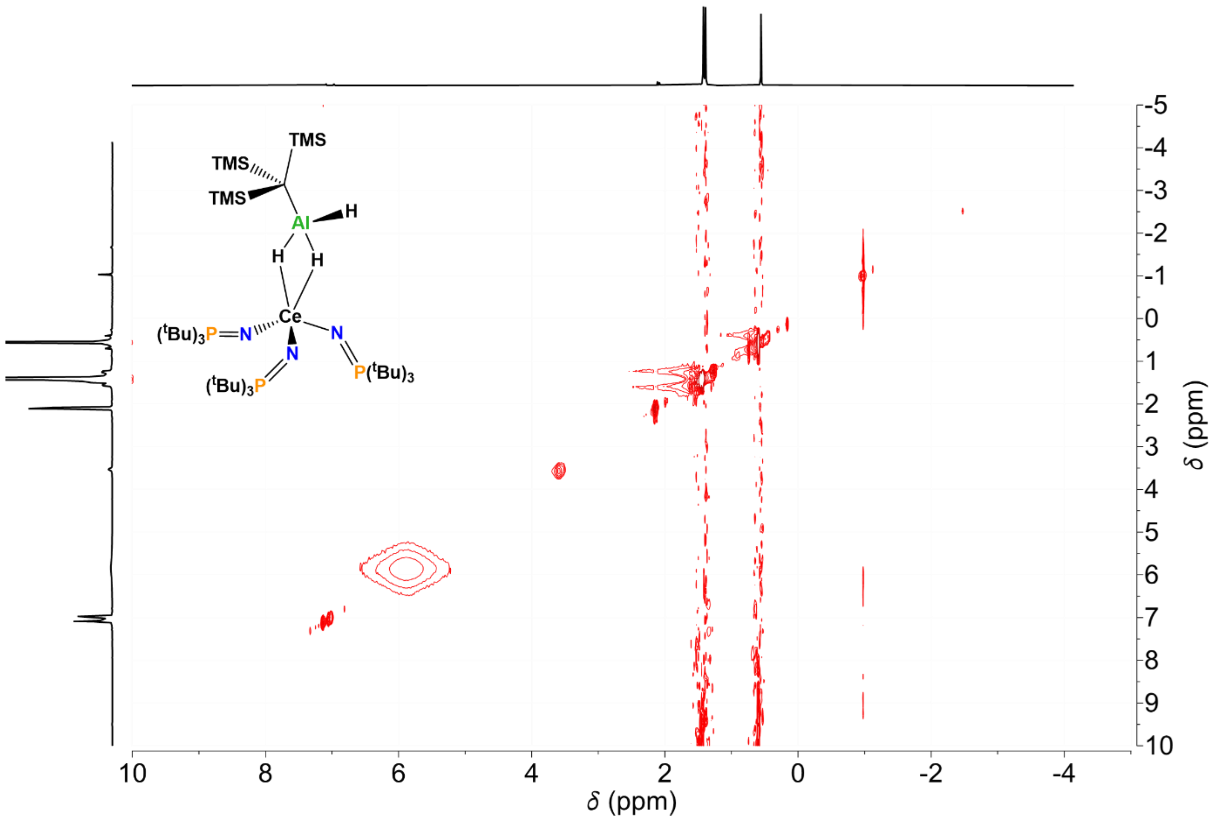

Figure S11: ^{1}H EXSY NMR of **CeHAl** in toluene-d$_8$ after heating to 92 °C and cooling back to room temperature.

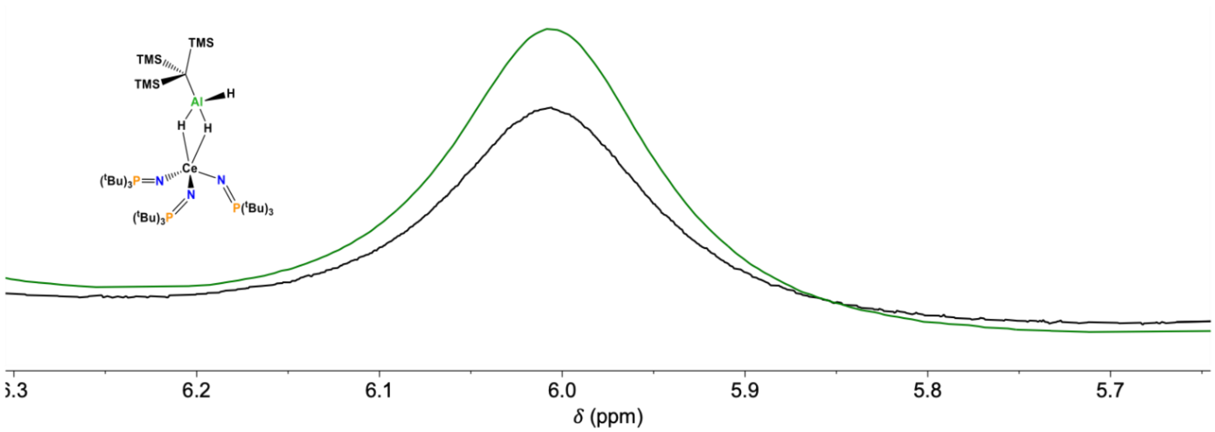

Figure S12: ^{1}H{^{27}Al} NMR (green trace) overlaid with ^{1}H NMR (black trace) of **CeHAl** in toluene-d$_8$ at -70 °C. A large change in the peak shape when decoupling from the ^{27}Al nucleus was not observed at low temperature.

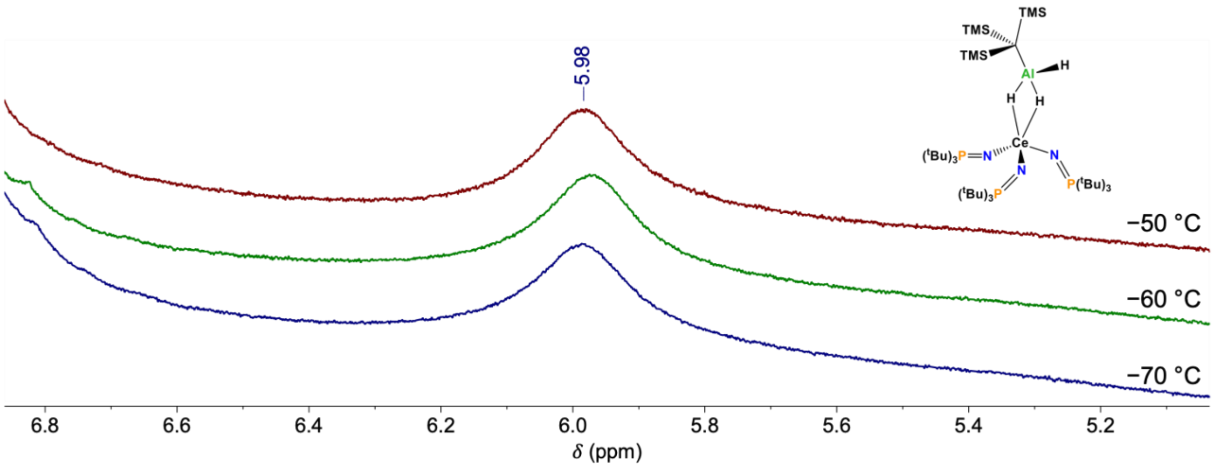

Figure S13: Variable temperature ^{1}H NMR of the hydride peak in **CeHAl** in toluene-d$_8$. The line width of the peak does not change as the temperature decreases.

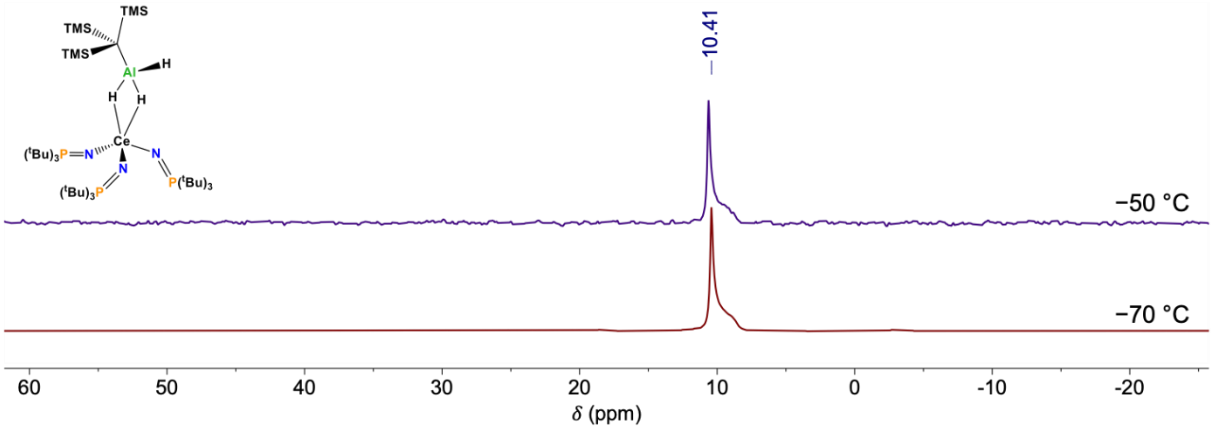

Figure S14: Variable temperature ^{31}P{^{1}H} NMR of **CeHAl** in toluene-d$_8$ at -50 °C and -70 °C.

***CeHAl** and Alkali Metal Reductions*

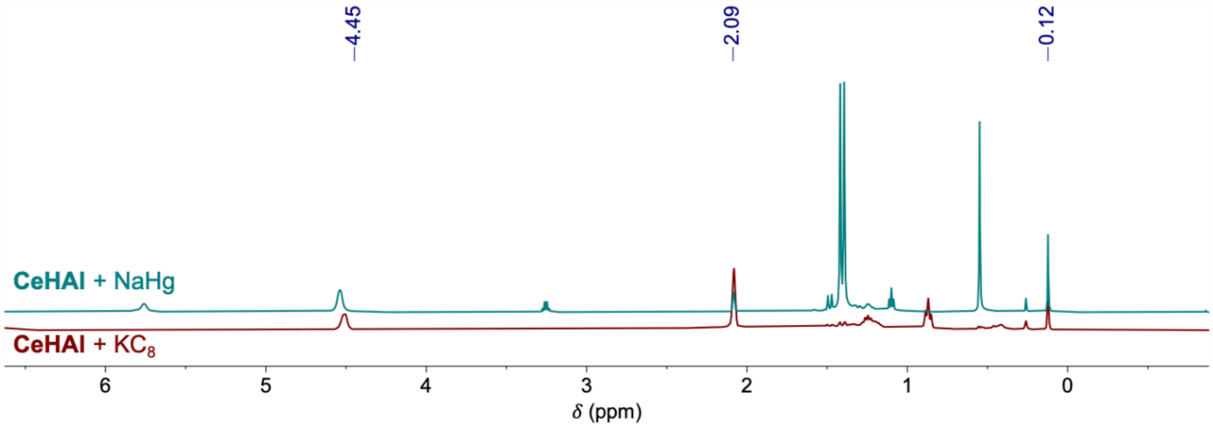

Figure S15: Overlay of ^{1}H spectra for the two alkali metal reductions of **CeHAl**. The common peaks are labeled in the spectrum.

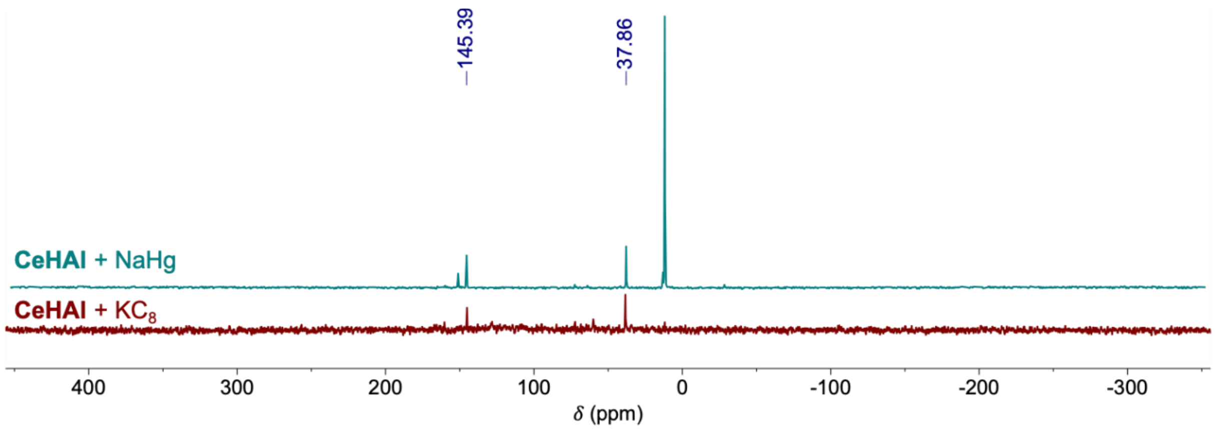

Figure S16: Overlay of $^{31}P\{^{1}H\}$ spectra for the two alkali metal reductions of **CeHAl**. The common peaks are labeled in the spectrum.

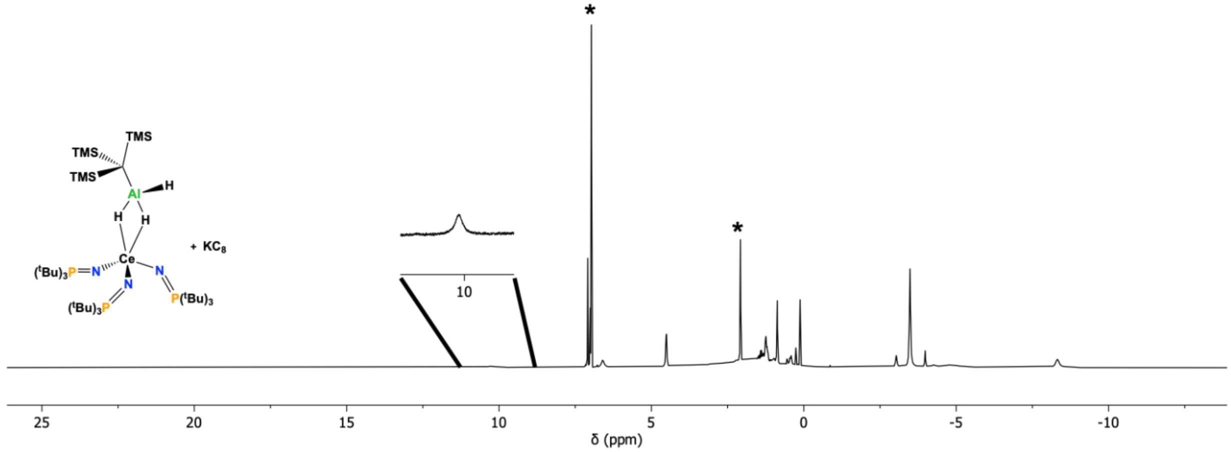

Figure S17: ^{1}H NMR of 11.4 mg **CeHAl** and 1.5 mg (1 equiv.) of KC_8 (NMR scale) in toluene-d_8. This reaction results in a mixture of products. Residual solvent is denoted with *.

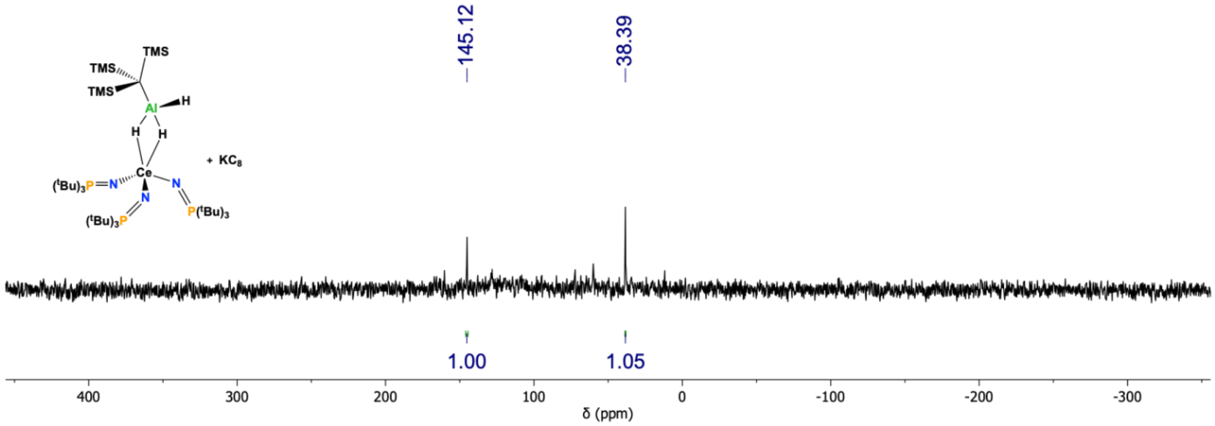

Figure S18: $^{31}P\{^{1}H\}$ NMR of 11.4 mg **CeHAl** and 1.5 mg (1 equiv.) of KC_8 (NMR scale) in toluene-d_8.

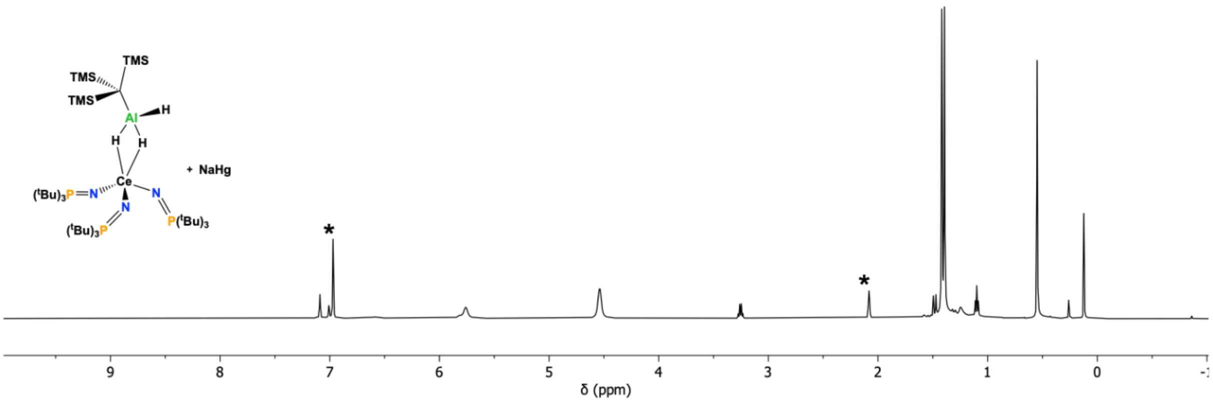

Figure S19: ^{1}H NMR of the reaction of **CeHAl** and NaHg amalgam (5% Na) in toluene-d_8. The reaction was worked up (see synthetic methods), yet the product appears to be a mixture. Residual solvent is denoted with *.

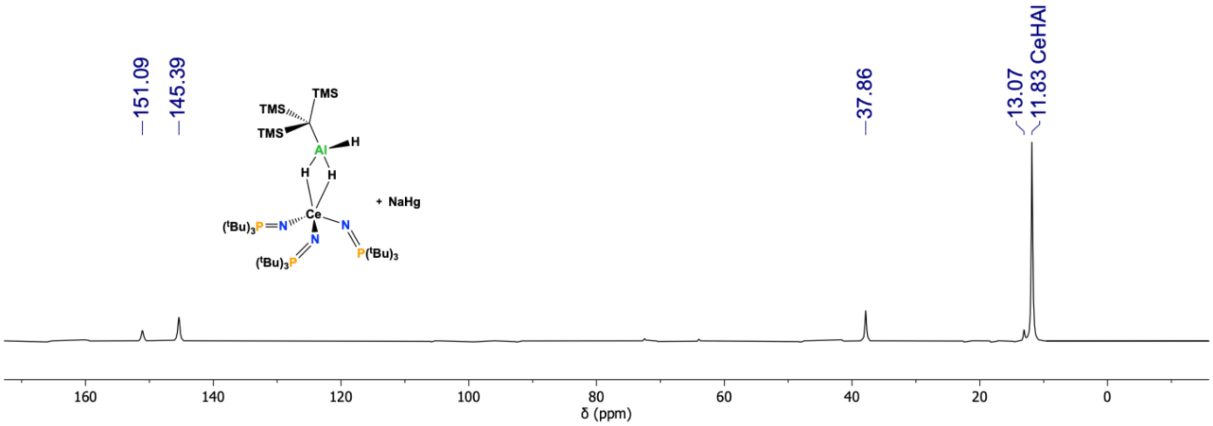

Figure S20: ^{31}P{^{1}H} NMR of the reaction of **CeHAl** and NaHg amalgam (5% Na) in toluene-d_8. The reaction was worked up (see synthetic methods), yet the product appears to be a mixture.

*Reduction of **CeHAl** by $CoCp_2$**

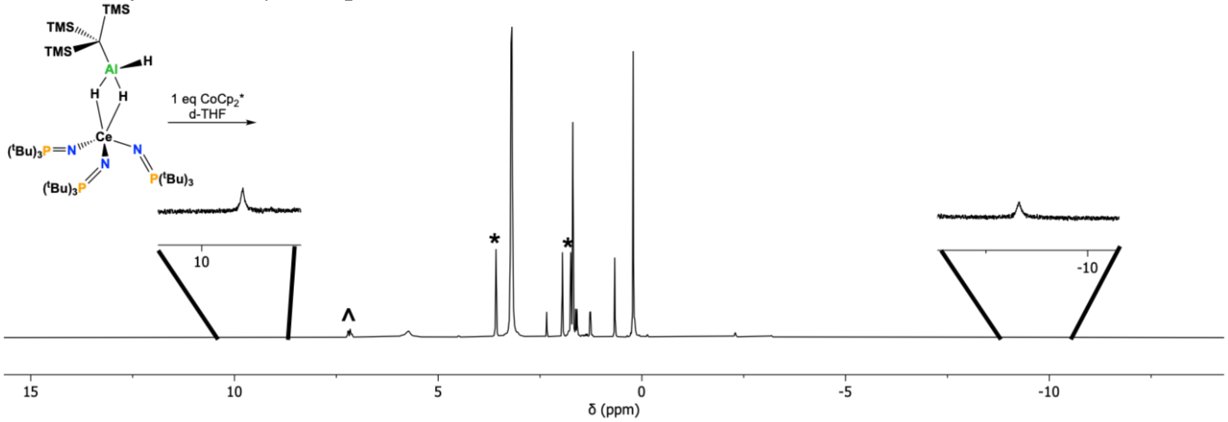

Figure S21: ^{1}H NMR of 9.2 mg **CeHAl** and 2.9 mg (1 equiv.) of decamethylcobaltocene ($CoCp_2$*) in d_8-THF. The reaction produces a mixture of products. Residual solvent is denoted in the figure with *. Residual toluene is denoted in the figure with ^.

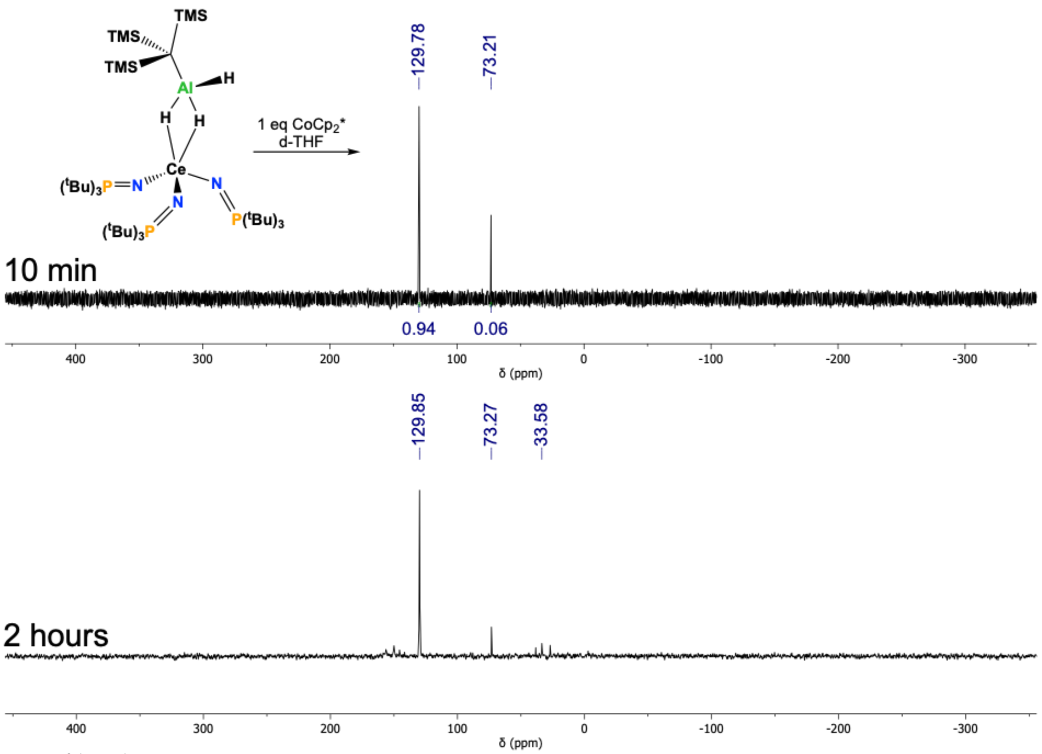

Figure S22: $^{31}P\{^1H\}$ NMR of 9.2 mg **CeHAl** and 2.9 mg (1 equiv.) of $CoCp_2^*$ in d_8-THF. Peak integrations are normalized to sum of 1.00 to illustrate percent composition of the reaction solution. The reaction results in a mixture of products. The initial primary product at 129.78 ppm has a short lifetime as observed by $^{31}P\{^1H\}$ NMR.

***CeHAl** and 4-dimethylaminopyridine*

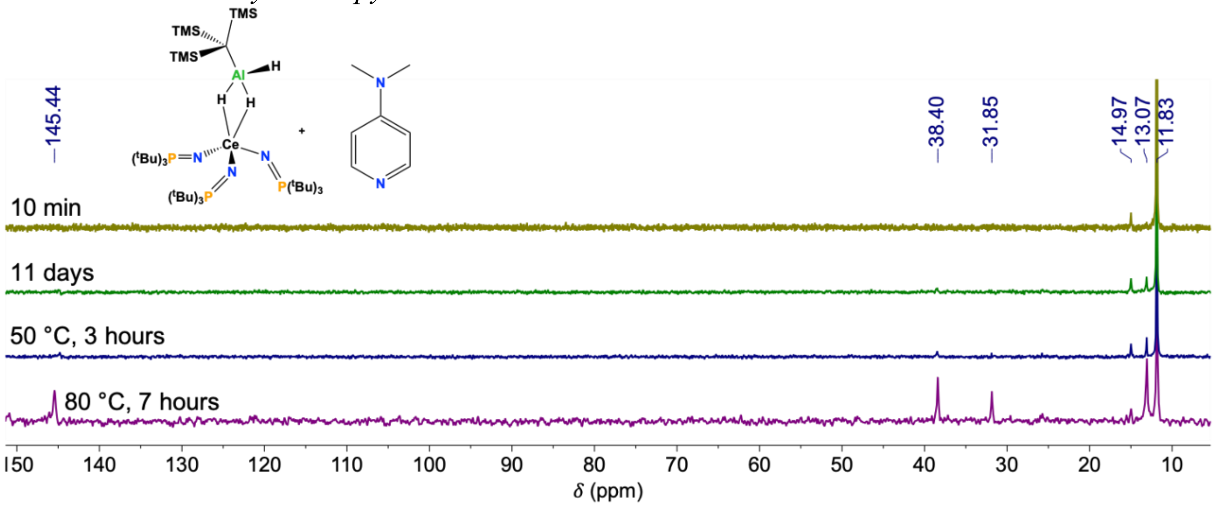

Figure S23: $^{31}P\{^1H\}$ NMR of 7.1 mg **CeHAl** and 0.95 mg (1 equiv.) of DMAP in toluene-d_8 over time and after heating. The identities of signals are as follows: δ 145.44, 38.40, 31.85 (undefined); 14.97 ($CeI(NP(^tBu)_3)_3$); 13.07 (undefined); 11.83 (unreacted **CeHAl**).

CeHAl and triphenylphospine oxide

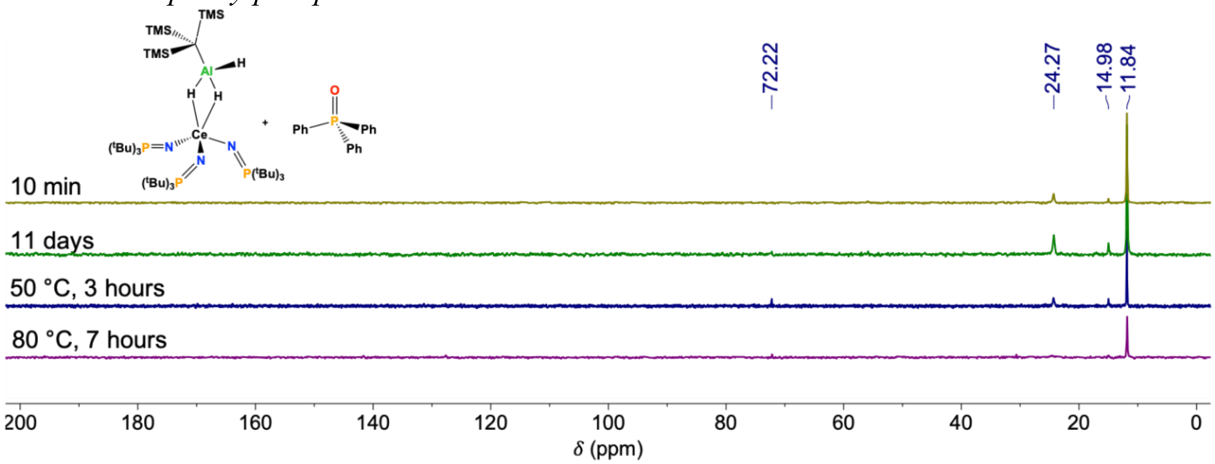

Figure S24: $^{31}P\{^{1}H\}$ NMR of 5.2 mg **CeHAl** and 1.58 mg (1 equiv. of $OPPh_3$ in toluene-d_8 over time and after heating. The identities of signals are as follows: δ 72.22, 24.27 (undefined); 14.98 ($CeI(NP(^{t}Bu)_3)_3$); 11.84 (unreacted **CeHAl**).

***CeHAl** and O_2*

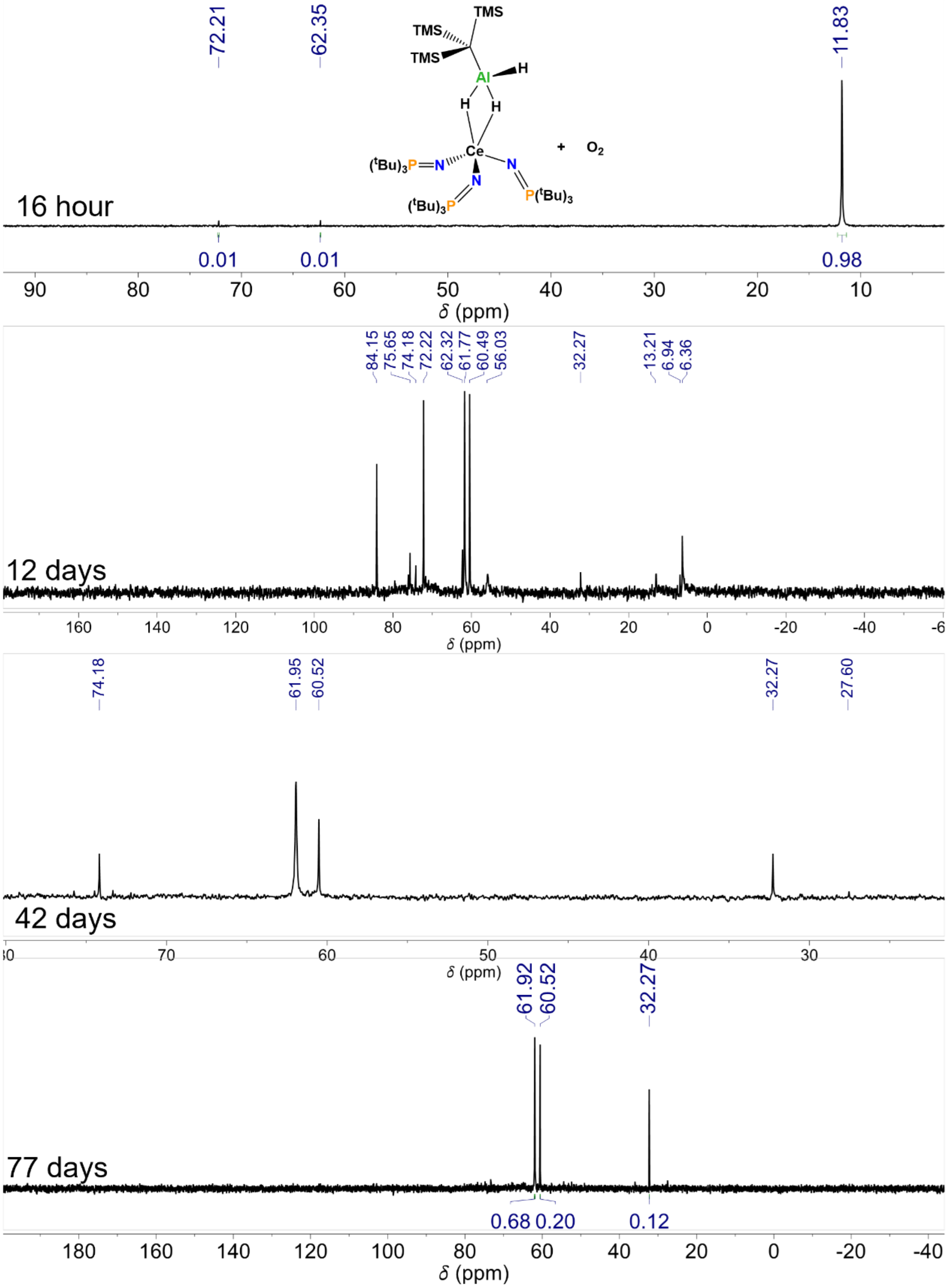

Figure S25: $^{31}P\{^{1}H\}$ NMR of **CeHAl** and excess O_2 in toluene-d$_8$ after 16 hours, 12 days, 42 days, and 77 days. Peak integrations are normalized to sum of 1.00 to illustrate percent composition of the reaction solution. The identities of the signal at δ 11.82 (unreacted **CeHAl**) is the only identified signal in the four spectra. On day 12, the solution was much darker than day 1. By day 42, the solution had become much lighter in color.

***CeHAl** and tertbutyl isocyanide*

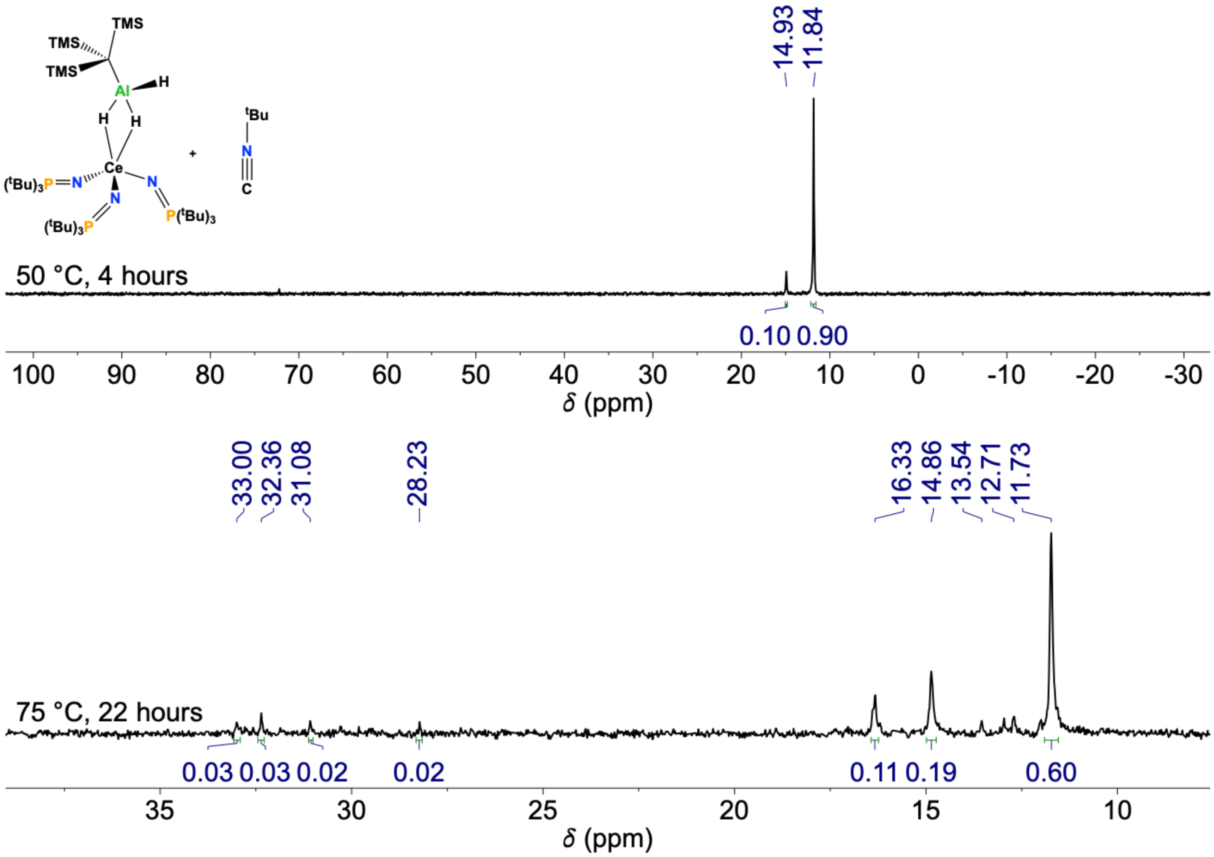

Figure S26: $^{31}P\{^{1}H\}$ NMR of 7.3 mg **CeHAl** and 1.3 mg (2.2 equiv.) of *tert*-butyl isocyanide (NMR scale) in toluene-d_8 after heating at 50 °C for 4 hours (top panel) and then at 75 °C for an additional 22 hours (bottom panel). Peak integrations are normalized to sum of 1.00 to illustrate percent composition of the reaction solution. The identities of the signals are as follows for the top panel: δ 14.93 (CeI(NP(^{t}Bu)$_3$)$_3$); 11.84 (unreacted **CeHAl**) and for the bottom panel: δ 33.00, 32.36, 31.08, 28.23, 16.33 (undefined); 14.86 (CeI(NP(^{t}Bu)$_3$)$_3$); 11.73; 13.54, 12.71 (undefined); 11.73 (unreacted **CeHAl)**.

***CeHAl** and N,N'-dicyclohexylcarbodiimide*

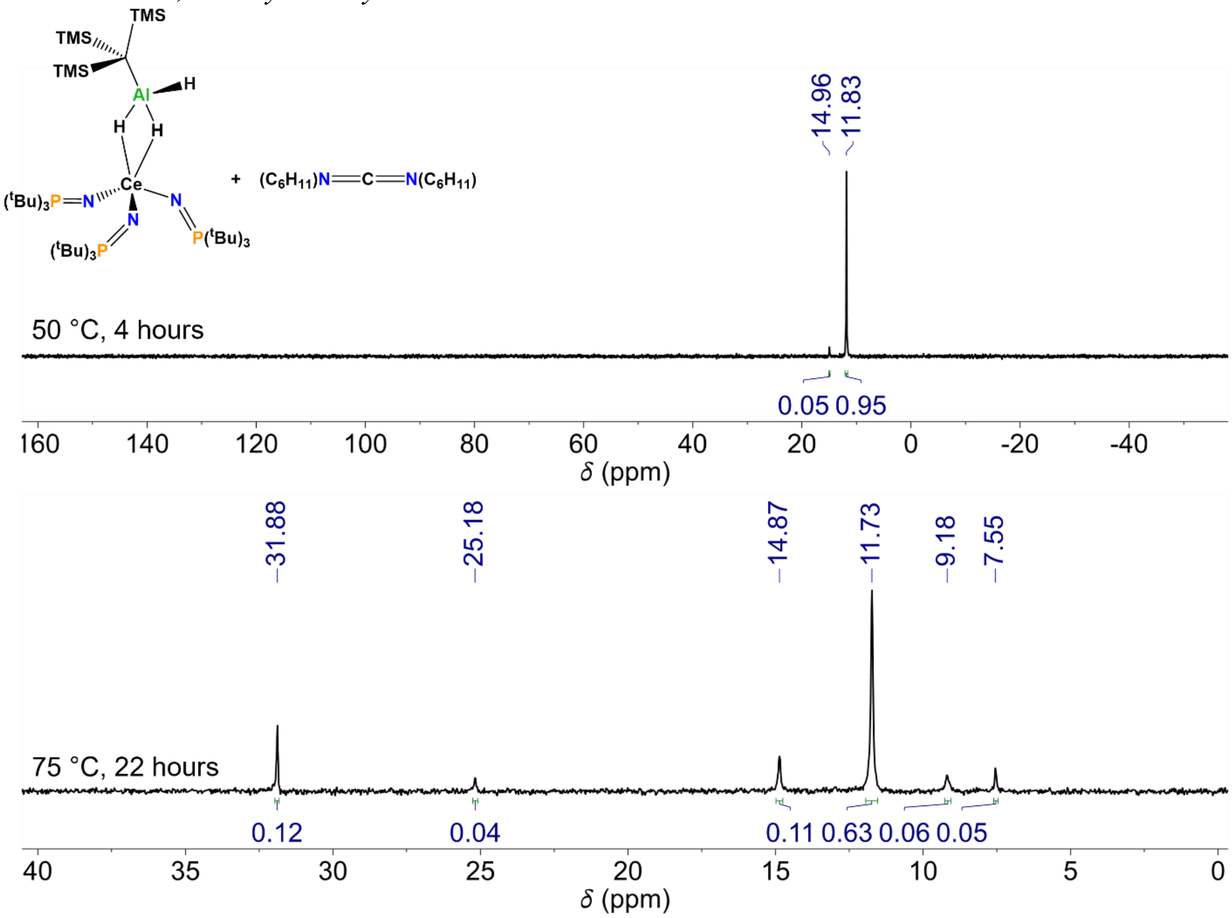

Figure S27: $^{31}P\{^{1}H\}$ NMR of 5.7 mg **CeHAl** and 1.4 mg (1.2 equiv.) of *N*-*N'*-dicyclohexylcarbodiimide (NMR scale) in toluene-d_8 after heating at 50 °C for 4 hours (top panel) and then at 75 °C for an additional 22 hours (bottom panel). Peak integrations are normalized to sum of 1.00 to illustrate percent composition of the reaction solution. The identities of the signals are as follows for the top panel: δ 14.96 (CeI(NP(^{t}Bu)$_3$)$_3$); 11.83 (unreacted **CeHAl)** and for the bottom panel: δ 31.88, 25.18 (undefined); 14.87 (CeI(NP(^{t}Bu)$_3$)$_3$); 11.73 (unreacted **CeHAl**); 9.18, 7.55 (undefined). Discrepancies in shift are attributed to referencing errors.

***CeHAl** and H_2*

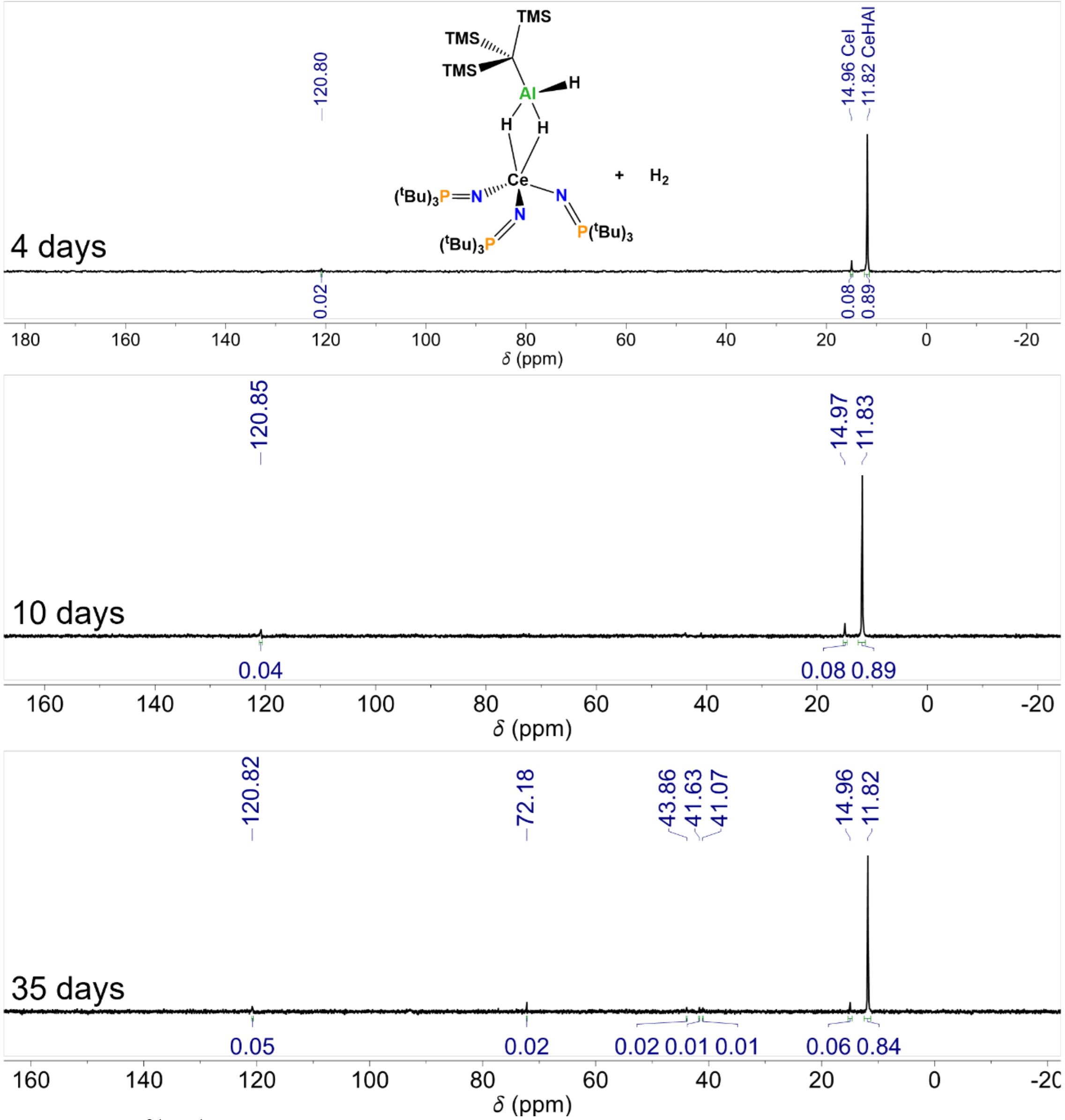

Figure S28: $^{31}P\{^1H\}$ NMR of **CeHAl** and excess H_2 in toluene-d_8 after 4 days, 10 days, and 35 days. Peak integrations are normalized to sum of 1.00 to illustrate percent composition of the reaction solution. The identities of the signals at δ 14.96 ($CeI(NP(^tBu)_3)_3$) and 11.82 (unreacted **CeHAl**) are the only identified signals across the three spectra.

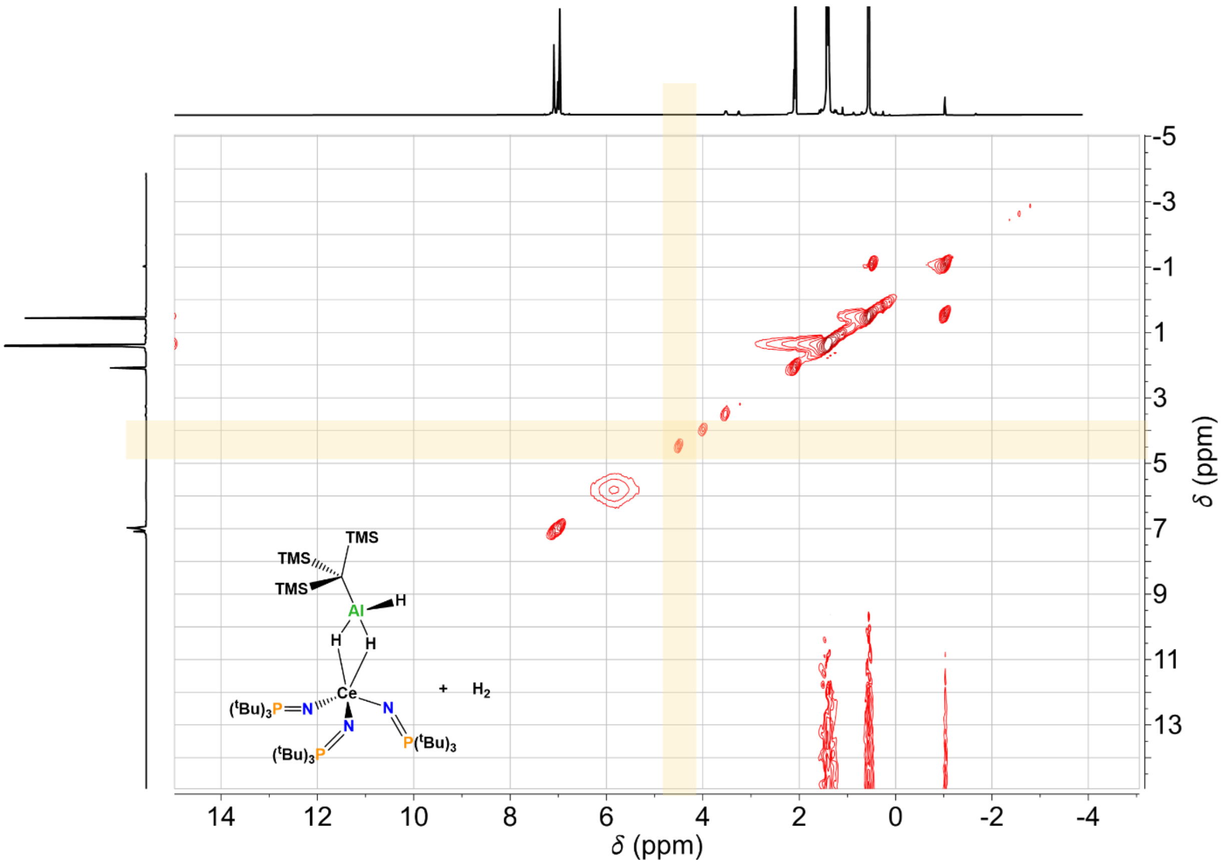

Figure S29: ^{1}H NOESY NMR of **CeHAl** and excess H_2 in toluene-d_8 after 10 days. The highlighted region is where intermolecular coupling with H_2 would be observed, if present. Notably, there is no coupling between **CeHAl** hydrides and H_2, indicating that there is no hydrogen exchange mechanism. Peaks indicating other observed coupling are assigned in the next figure.

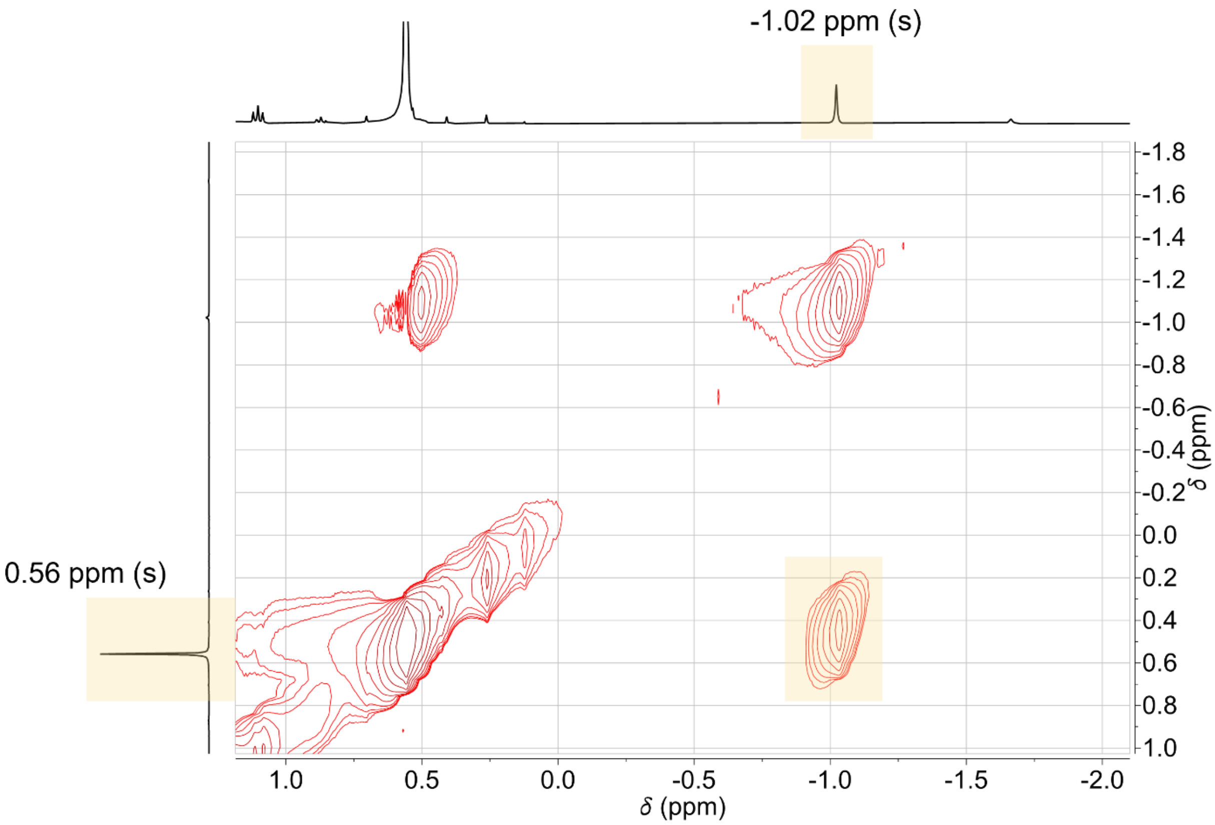

Figure S30: Enlarged area of ^{1}H NOESY NMR of **CeHAl** and excess H_2 in toluene-d_8 after 10 days. Highlighting the cross-peak resulting from coupling between the TMS group on **CeHAl** and a peak at -1.02 ppm. This coupling was not observed in ^{1}H EXSY NMR of neat **CeHAl** (**Figure S11**). Notably, there is no coupling between **CeHAl** hydrides and H_2, indicating that there is no hydrogen exchange mechanism.

***CeHAl** and benzhydrol*

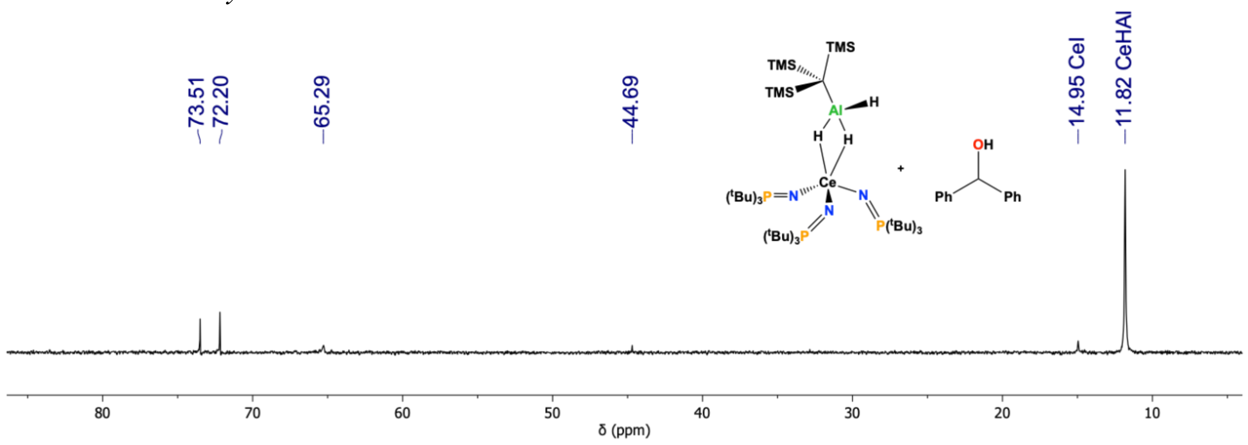

Figure S31: $^{31}P\{^{1}H\}$ NMR of 9.1 mg **CeHAl** and 1.6 mg (1 equiv.) of benzhydrol (NMR scale) in toluene-d_8 after 18 hours. The identities of the signals are as follows: δ 73.90, 72.20, 65.29, 44.69 (undefined); 14.95 ($CeI(NP(^tBu)_3)_3$); 11.82 (unreacted **CeHAl**).

***CeHAl** and $(C_6F_5)_3B(H_2O)$*

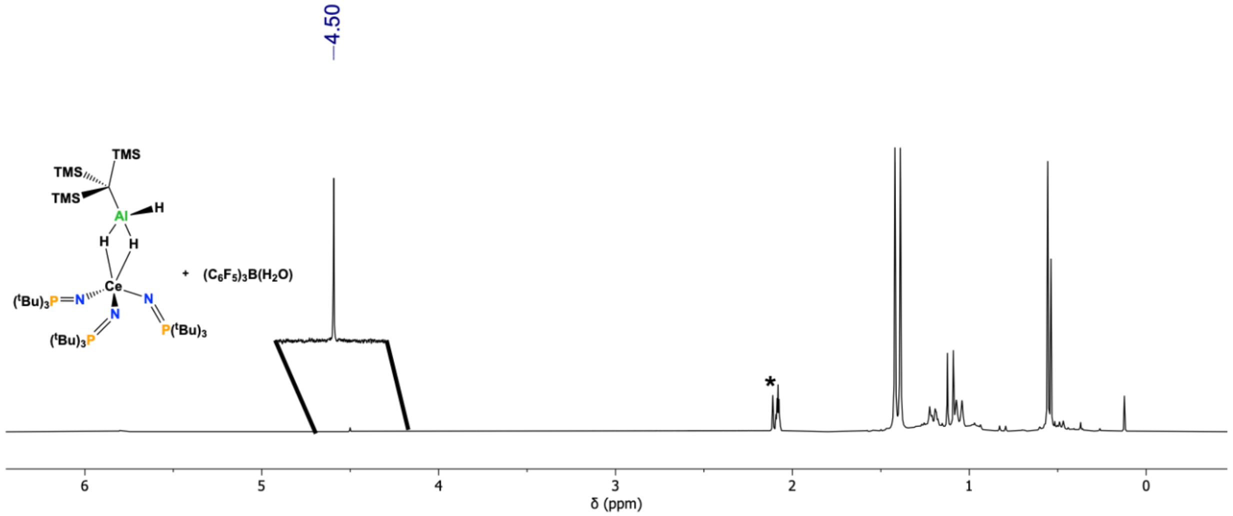

Figure S32: ^{1}H NMR of 7.5 mg **CeHAl** and 3.7 mg (1 equiv.) of $(C_6F_5)_3B(H_2O)$ (NMR scale) in toluene-d_8. Upon addition of $(C_6F_5)_3B(H_2O)$, H_2 gas was produced as evidenced by the peak at 4.50 ppm.

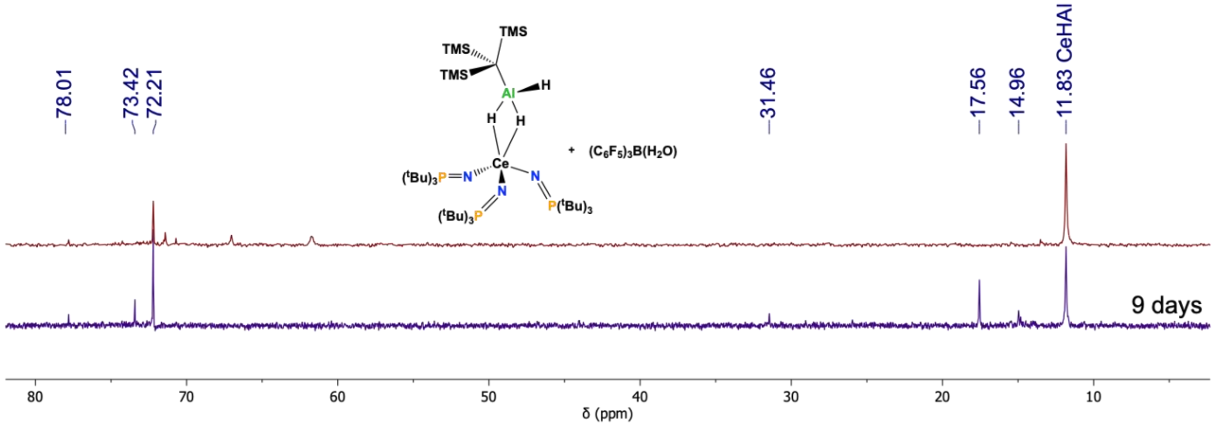

Figure S33: $^{31}P\{^1H\}$ NMR of 7.5 mg **CeHAl** and 3.7 mg (1 equiv.) of $(C_6F_5)_3B(H_2O)$ (NMR scale) in toluene-d_8 immediately upon addition and 9 days later. The identities of the signals are as follows: δ 78.01, 73.42, 72.21, 31.46, 17.56 (undefined); 14.96 ($CeI(NP(^tBu)_3)_3$ (present in original sample, it is not formed in the reaction); 11.83 (unreacted **CeHAl**).

***CeHAl** and benzophenone*

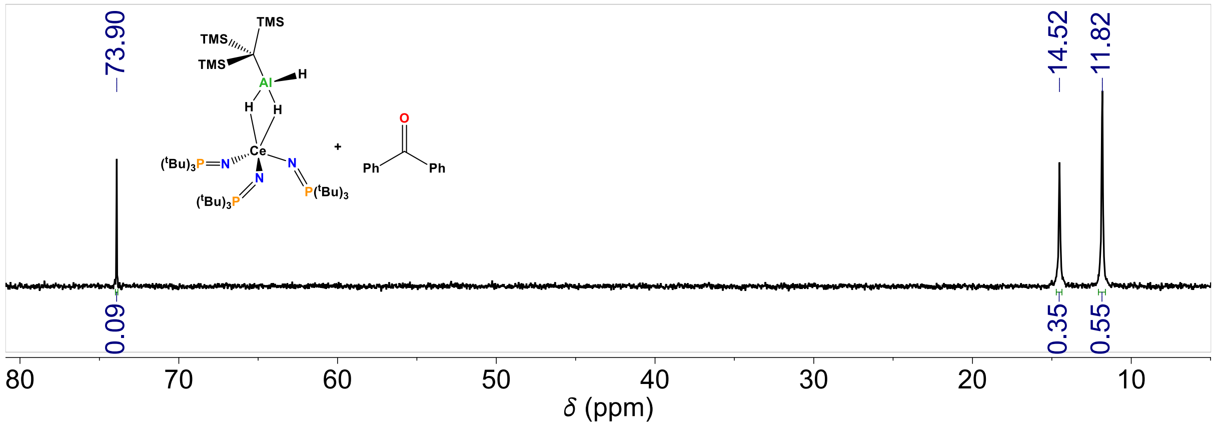

Figure S34: $^{31}P\{^{1}H\}$ NMR of 6.3 mg **CeHAl** and 1.3 mg (1 equiv.) of benzophenone (NMR scale) in toluene-d_8 after 18 hours. Peak integrations are normalized to sum of 1.00 to illustrate percent composition of the reaction solution. The identities of the signals are as follows: δ 73.90 (undefined); 14.52 (**$CeOPh_2$**); 11.82 (unreacted **CeHAl**).

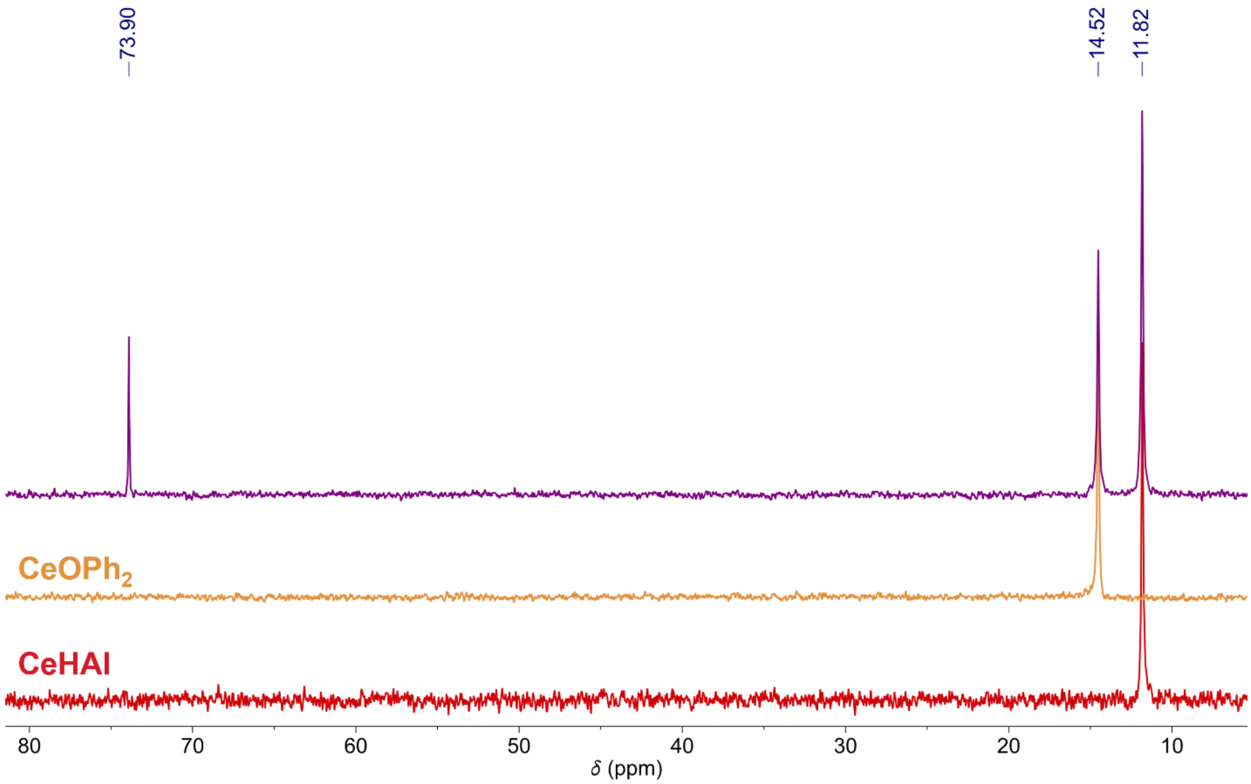

Figure S35: $^{31}P\{^{1}H\}$ NMR of 6.3 mg **CeHAl** and 1.3 mg (1 equiv.) of benzophenone (purple trace), **$CeOPh_2$** (orange trace), and **CeHAl** (red trace) in toluene-d_8.

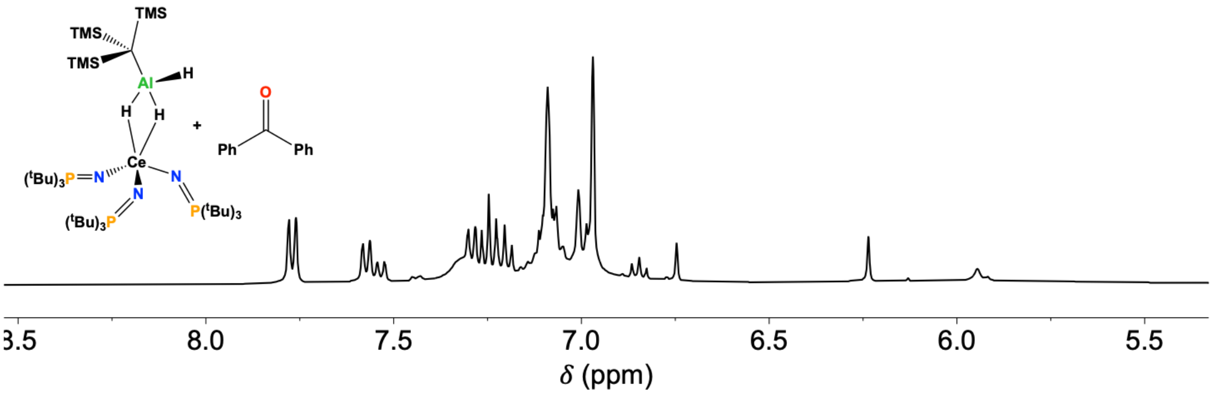

Figure S36: ^{1}H NMR aromatic region of 6.3 mg **CeHAl** and 1.3 mg (1 equiv.) of benzophenone (NMR scale) in toluene-d$_8$ after 18 hours.

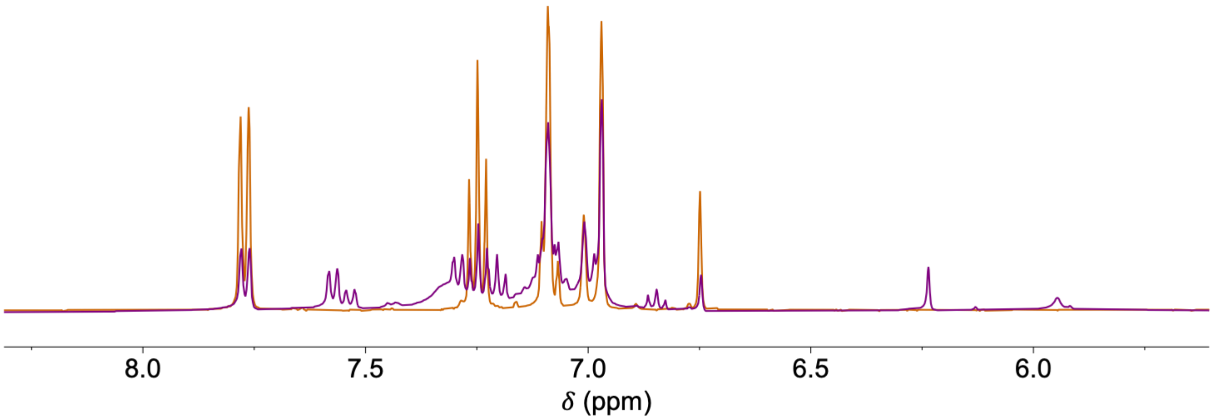

Figure S37: ^{1}H NMR aromatic region of 6.3 mg **CeHAl** and 1.3 mg (1 equiv.) of benzophenone (NMR scale) in toluene-d$_8$ after 18 hours (purple trace) overlaid with **CeOPh$_2$** (orange trace) to highlight the aromatic features unique to the reaction mixture.

CeOPh$_2$

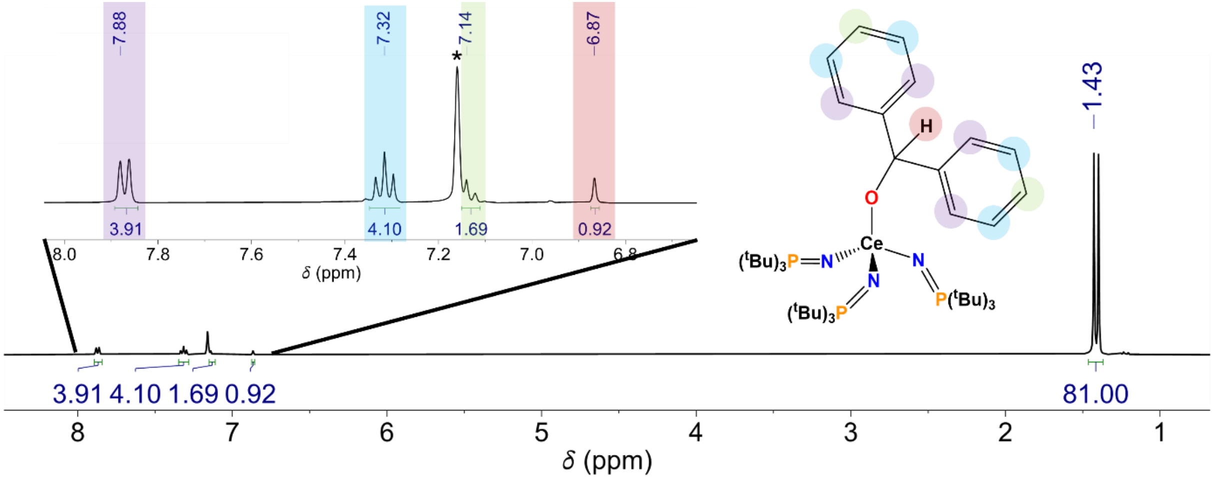

Figure S38: ^{1}H NMR of **CeOPh$_2$** in C_6D_6. Residual solvent is denoted in the figure with *.

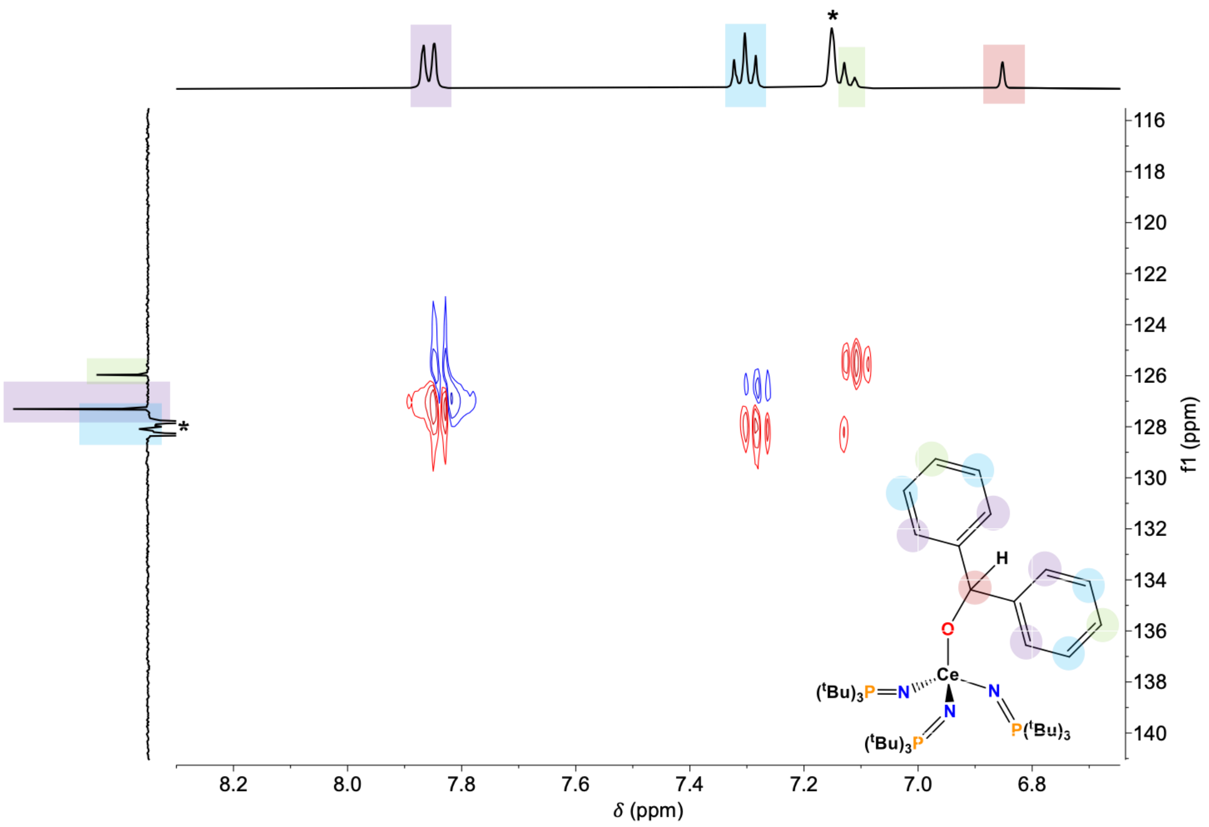

Figure S39: HSQC ^{1}H-^{13}C NMR of the aromatic region of **CeOPh2** in C_6D_6. Residual solvent is denoted in the figure with *.

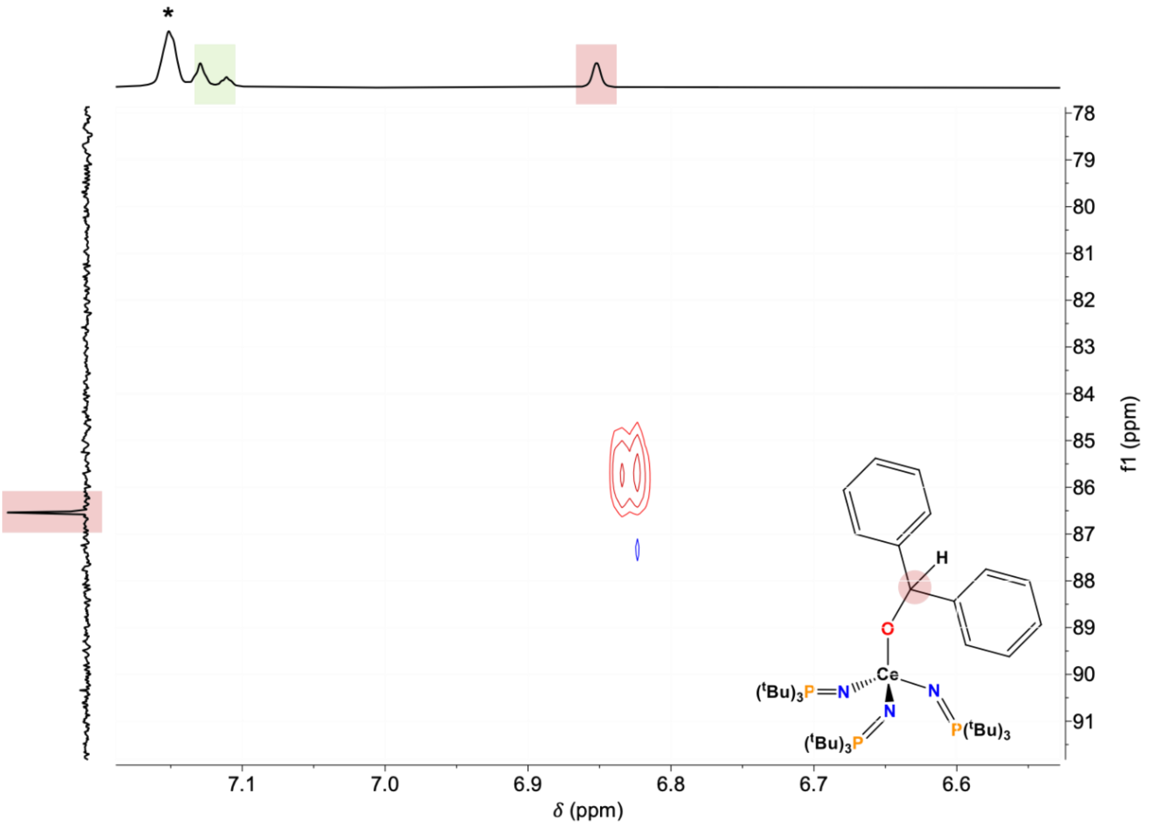

Figure S40: HSQC ^{1}H-^{13}C NMR of the -OCH region of **CeOPh$_2$** in C_6D_6. Residual solvent is denoted in the figure with *.

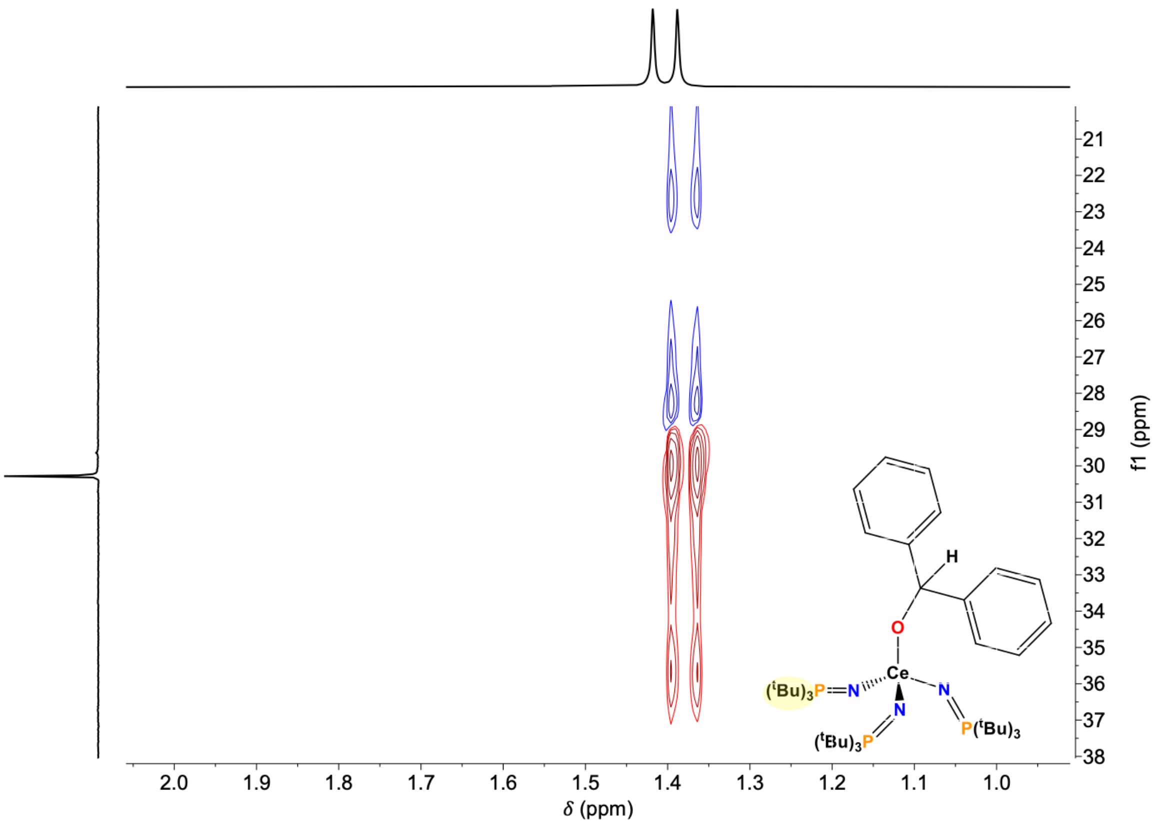

Figure S41: HSQC ^{1}H-^{13}C NMR of the imidophosphorane region of **CeOPh2** in C_6D_6.

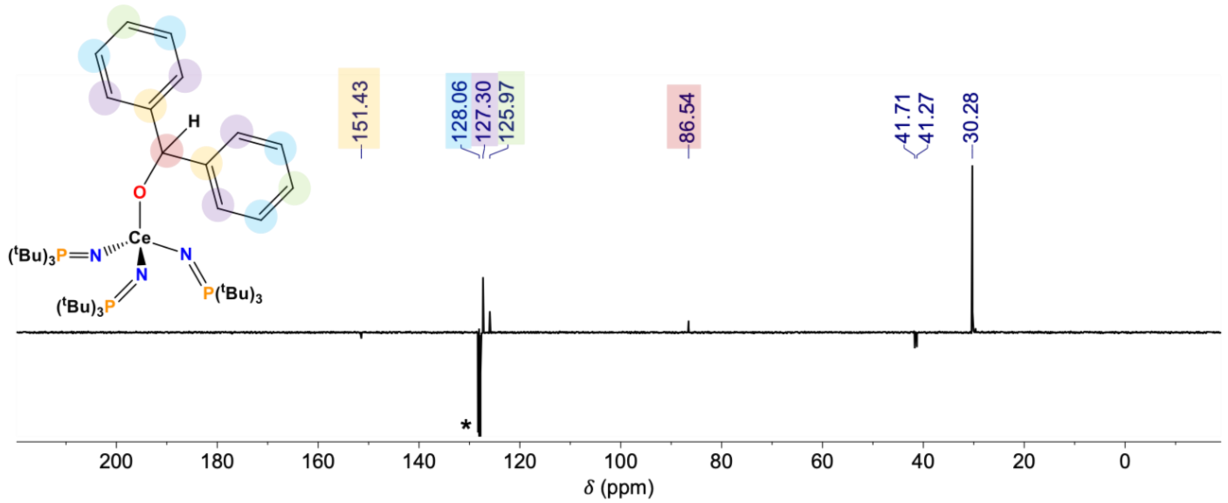

Figure S42: DEPTQ135 $^{13}C\{^{1}H\}$ NMR of **CeOPh2** in C_6D_6. Residual solvent is denoted in the figure with *.

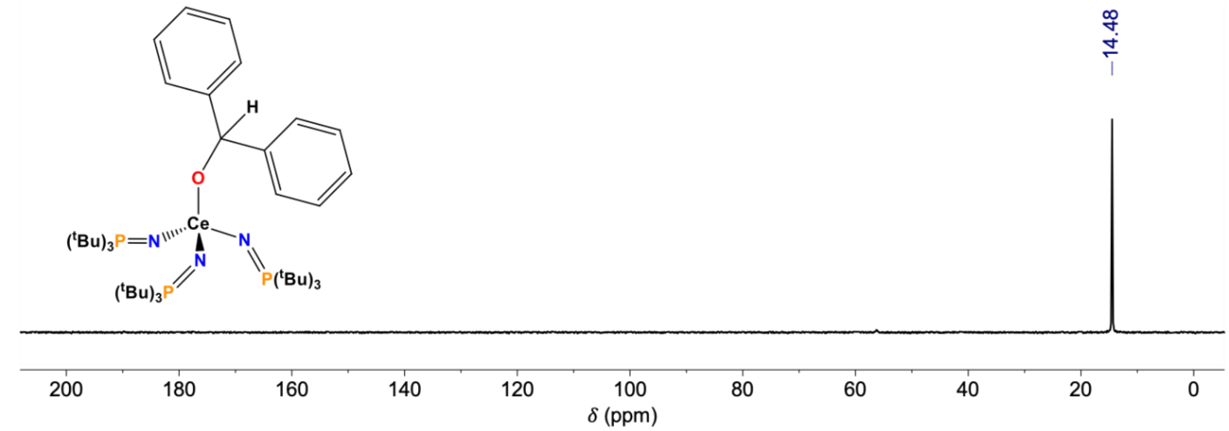

Figure S43: $^{31}P\{^{1}H\}$ NMR of **CeOPh₂** in C_6D_6.

Electrochemistry

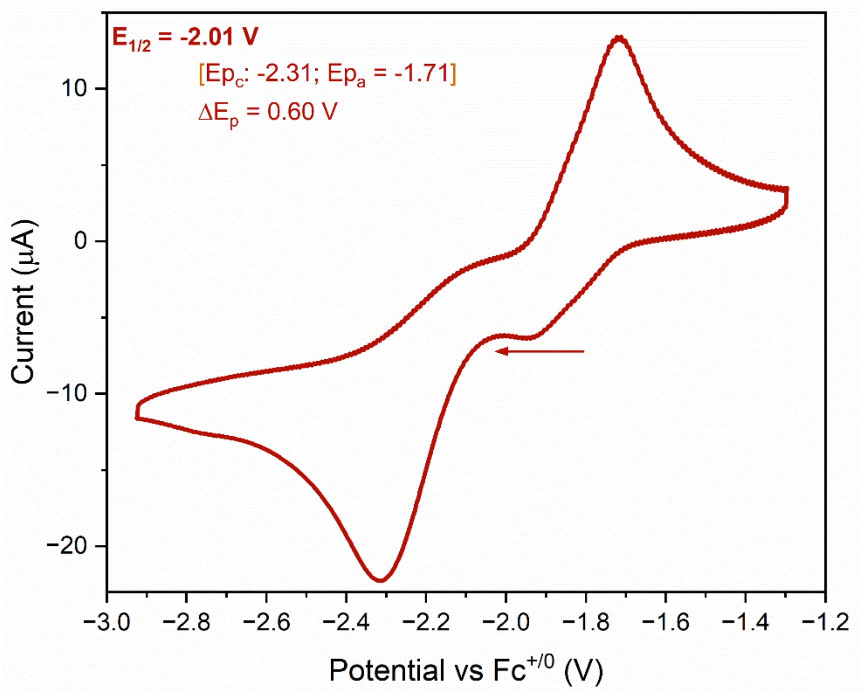

Figure S44: Cyclic voltammogram of **CeHAl** (1.72 mM in 0.05 M [^{n}Bu$_4$N][BPh$_4$] in THF). WE: GC; CE: Pt wire; RE: fritted Ag^0 wire; iR compensation of 620 Ω; scan rate 200 mV/sec. The arrow indicates scan direction and the starting potential of the sweep.

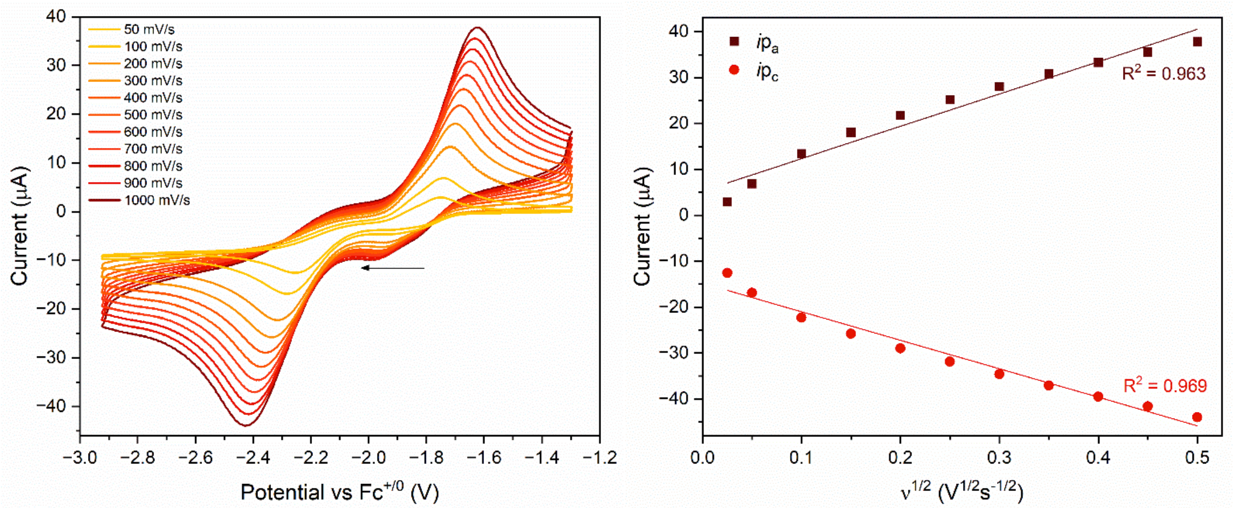

Figure S45: Left: scan-rate dependence of 1.7 mM **CeHAl** in 0.05 M [^{n}Bu$_4$N][BPh$_4$] in THF. Right: The corresponding Randles-Sevcik plot. WE: GC; CE: Pt wire; RE: fritted Ag^0 wire; iR compensation of 620 Ω.

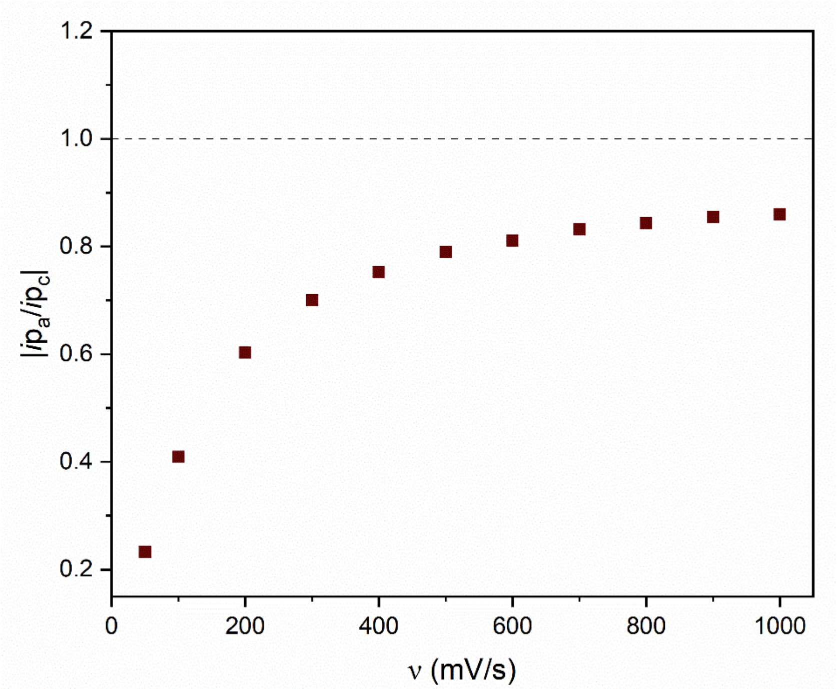

Figure S46: Ratio of anodic and cathodic peak currents for 1.7 mM **CeHAl** in 0.05 M [^{n}Bu$_4$N][BPh$_4$] in THF. A peak current ratio of 1 indicates a chemically reversible process in which the reduced compound is stable in solution.[17]

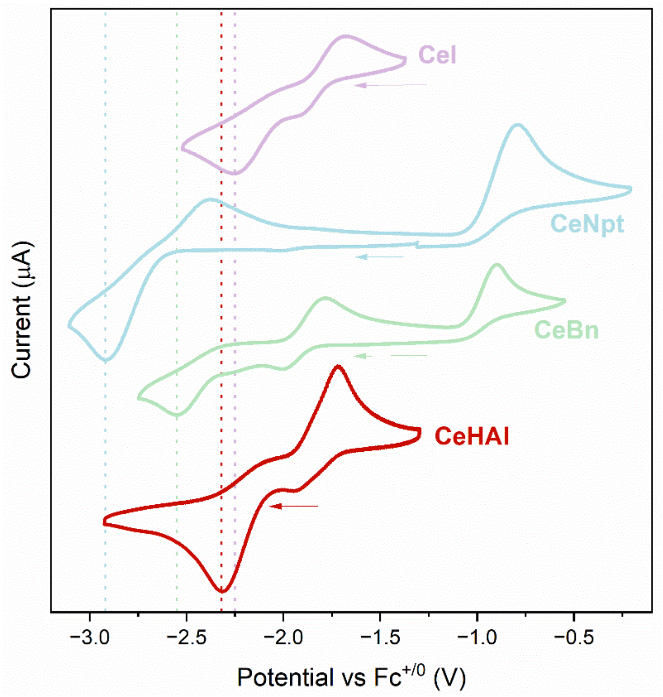

Figure S47: Overlay of cyclic voltammograms for CeI(NP(^{t}Bu)$_3$)$_3$, CeNpt(NP(^{t}Bu)$_3$)$_3$, and CeBn(NP(^{t}Bu)$_3$)$_3$ (abbreviated as CeI, CeNpt, CeBn respectively). The reduction potential for each complex is indicated by a dotted line in the corresponding color. CeNpt and CeBn have two oxidative features, assigned to the metal (more negative) and the ligand (less negative). CeI: 3 mM in 0.1 M [^{n}Bu4N][BPh4] in THF. CeNpt and CeBn: 3 mM in 0.2 M [^{n}Bu4N][PF6] in PhF. All collected with WE: GC; CE: Pt wire; RE: fitted Ag^0 wire; scan rate 200 mV/sec. The arrow indicates scan direction and the starting potential of the sweep. Data for CeI, CeNpt, and CeBn are reproduced from published data.[2,9]

Infrared Spectroscopy

CeHAl exhibits two weak vibrational modes associated with the hydrides at 1780 and 1633 cm^{-1}. Based on previously published work with this aluminum hydride ligand scaffold in which both κ^2 and κ^3 geometries are observed (Ti: κ^2, Th and U: κ^3).[4,18] The FT-IR spectra across the three compounds and two different binding modes are similar, such that determination of binding mode cannot be made based on symmetry considerations and the characteristic features present in FT-IR. The Ti complex exhibits one medium feature at 1831 cm^{-1} and one weak broad feature at approximately 1600 cm^{-1}. The Th and U complexes exhibit two weak broad features around 1680 cm^{-1} (Th) and 1574 cm^{-1} (U). In both the Th and U spectrum, the mode at larger wavenumber is slightly stronger. As such, **CeHAl** exhibits vibrational modes that are sharper than those previously reported but exhibit similar intensity and number of features as both κ^2 and κ^3 geometries. No information pertaining to the ambiguous bonding mode in **CeHAl** can thus be determined based on extant literature.

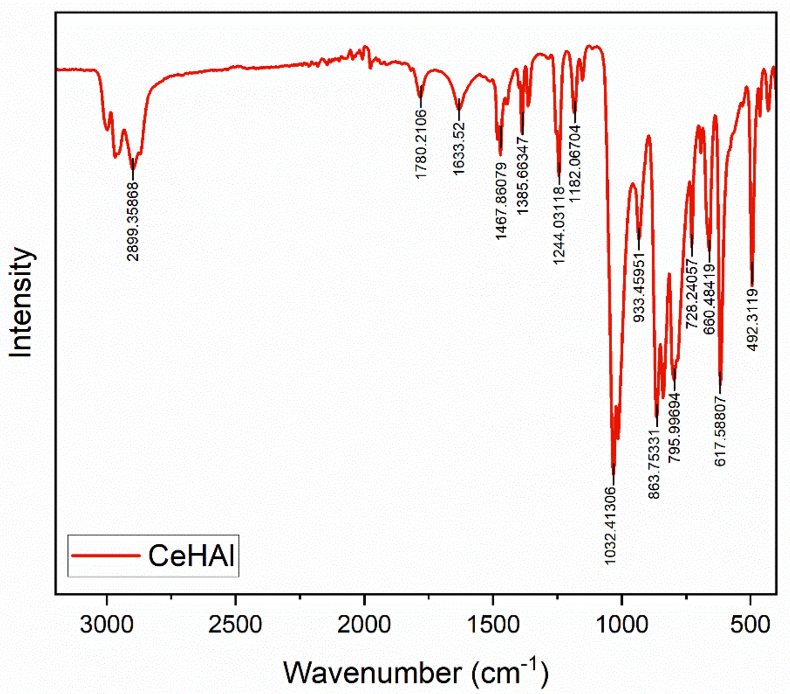

Figure S48: Solid-state IR spectrum of **CeHAl**, with modes labeled.

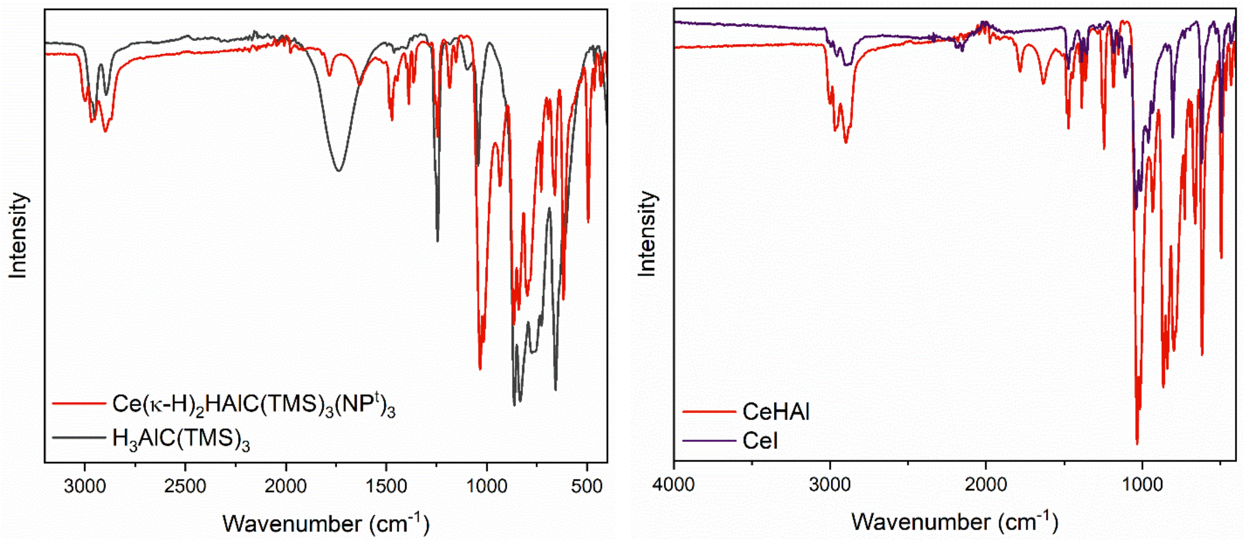

Figure S49: Solid-state IR spectra for **CeHAl** overlaid with Li[$H_3AlC(TMS)_3$] (left) and CeI(NP(^{t}Bu)$_3$)$_3$ (abbreviated as CeI) (right) to demonstrate which modes are unique to **CeHAl**: 1780 cm^{-1} and 1633 cm^{-1}.

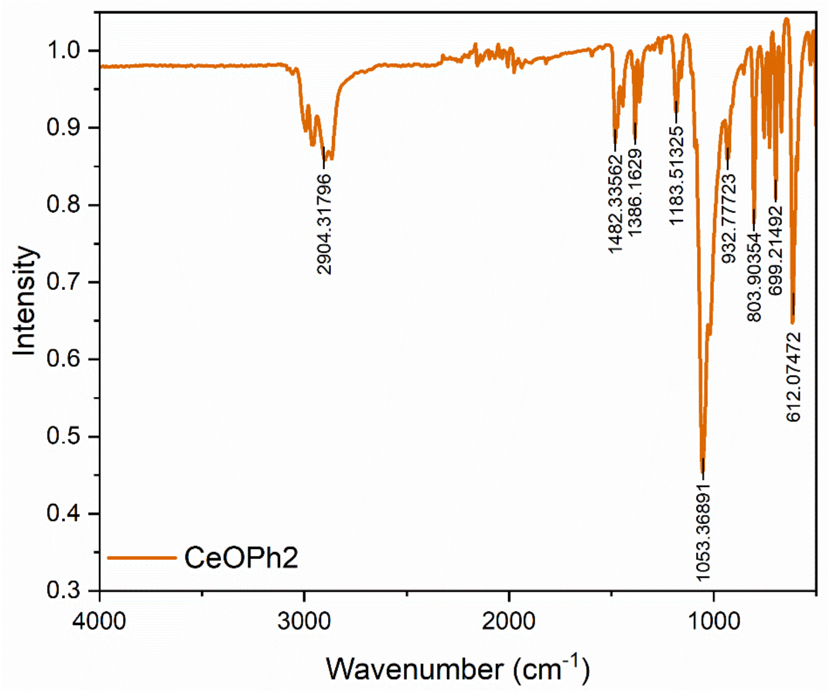

Figure S50: Solid-state IR spectrum of **$CeOPh_2$**.

UV-vis-NIR Electronic Absorption Spectra

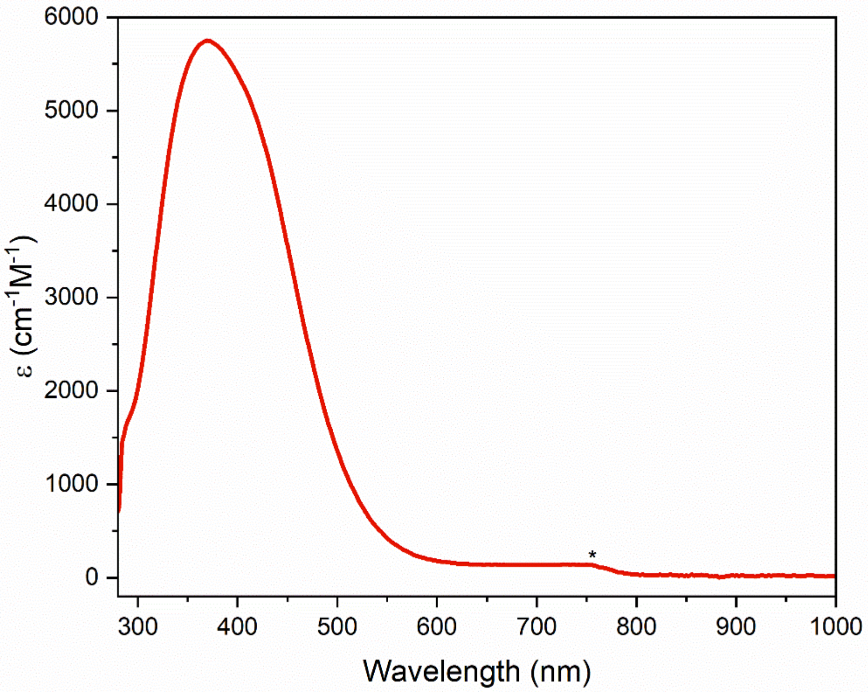

Figure S51: Molar absorptivity of **CeHAl** (140 μM in toluene) at 298 K. * indicates a solvent artifact.

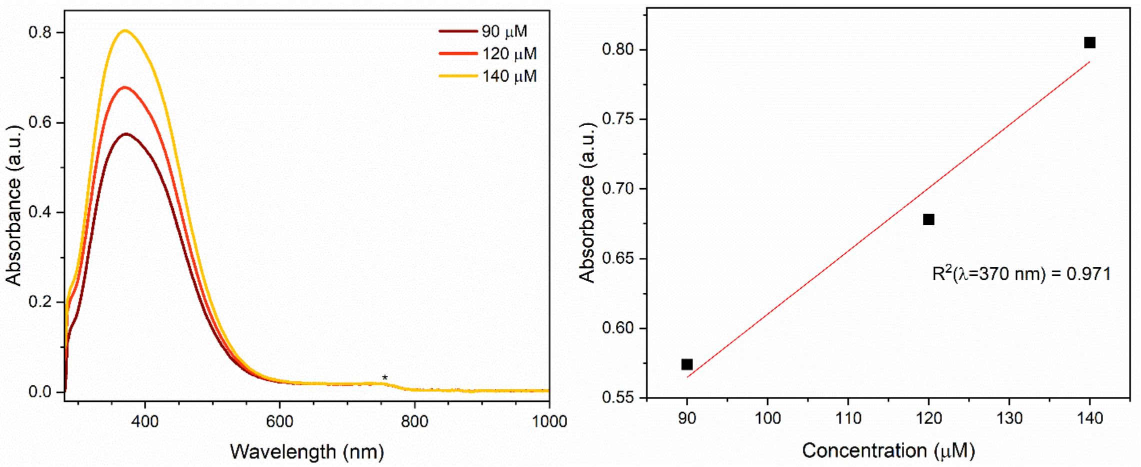

Figure S52: UV-vis-NIR spectra of **CeHAl** in toluene (left) and the corresponding linear regression of the absorbance at λ_{max} = 370 nm (right). * indicates a solvent artifact.

Fitting Details: The LMCT feature associated with **CeHAl** (λ_{max} = 370 nm, ε = 5750 M^{-1} cm^{-1}) was fit using a Gaussian function in Origin Lab using the Multi-Peak Fit function. Both the area and offset of the two peaks were set to be the same variable (i.e. one value for A and one value for y_0). A Jacobian transformation[19] was applied to the data to account for the conversion to energy from wavelength.

Table S1: Fit parameters for LMCT feature in the UV-Vis-NIR spectrum of **CeHAl**.

	**Energy (eV)**	**Energy (nm)**	**FWHM (eV)**	**Area**
**Peak 1**	3.499(1)	354.4	0.868(4)	4.17×10^5
**Peak 2**	2.8847(6)	429.8	0.743(3)	4.17×10^5

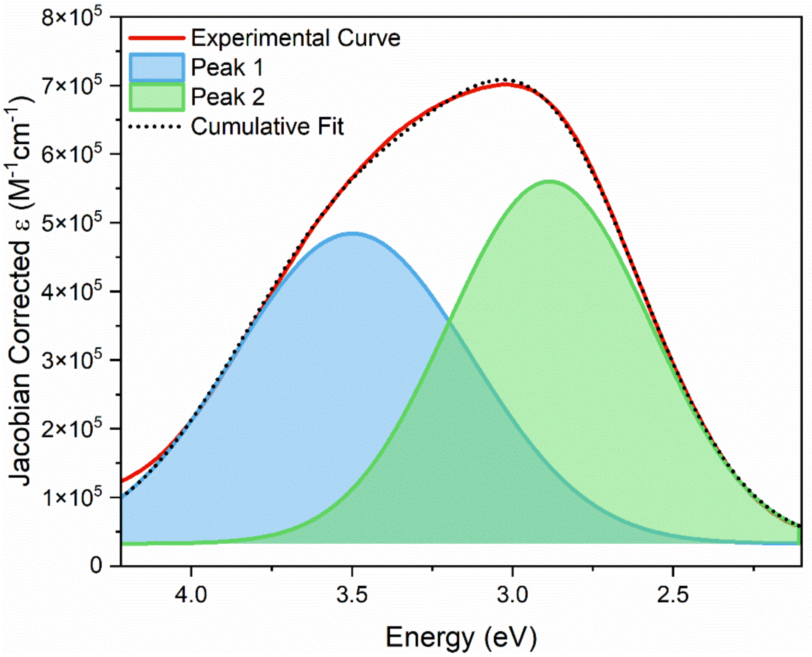

Figure S53: Fit of the LMCT feature in the UV-Vis-NIR spectrum of **CeHAl** using a Gaussian function implemented in Origin.

X-Ray Absorption Near-Edge Spectroscopy

Cerium L_3-edge transmission XANES data for $CeI(NP(^tBu)_3)_3$, $CeNpt(NP(^tBu)_3)_3$, and $CeBn(NP(^tBu)_3)_3$ and $CsCe(NP(^tBu)_3)_4$ have been previously reported, and the published normalized data were replotted and used for this analysis.[10,11]

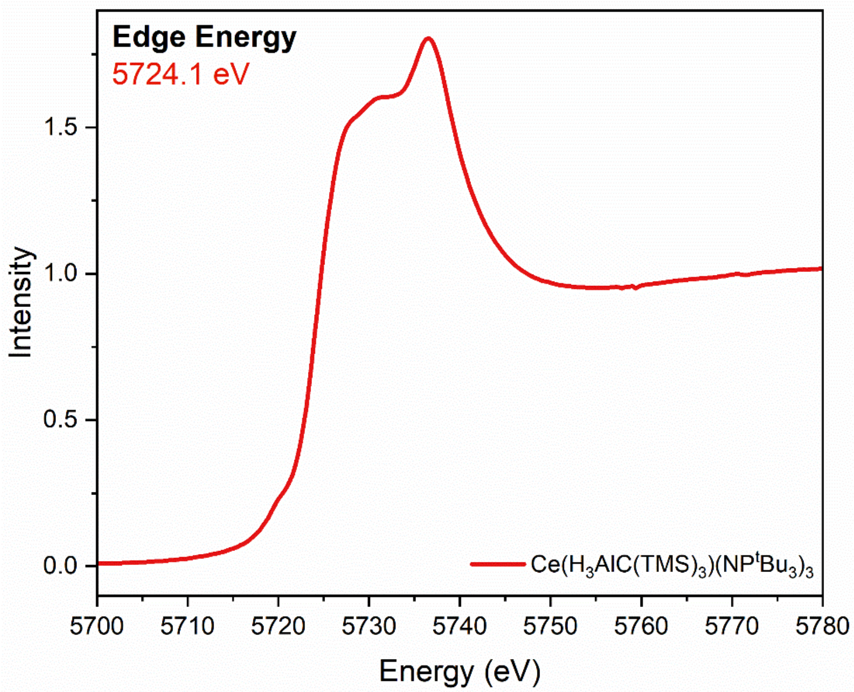

Figure S54: Cerium L_3-edge transmission XANES spectrum of **CeHAl** with a cerium edge energy of 5724.1 eV.

Fitting Details: CeHAl was fit using least-squares curve fitting as implemented in the LMFIT software in Python.[20] Transitions were modeled using a pseudo-Voigt function (50/50 Gaussian-Lorentzian). The parameter σ was fixed at 3.35 eV, in which σ is half of the FWHM for each component. The multi-peak feature at the L_3-edge, characteristic of tetravalent lanthanides, has been previously described as excitations to the 5d band, associated with final states of the $4f^n5d^1$ configuration and a formally trivalent configuration and ligand hole (L), $4f^{n+1}L5d^1$.[10,11]

Table S2: Fit parameters for cerium L_3-edge transmission XANES of **CeHAl**.

	Energy (eV)	σ	Area
**Step**	5728(2)	3(fixed)	-
**Pre-Edge**	5718(2)	3.35(fixed)	0.19(1)
**p2**	5726.8(1)	3.35(fixed)	7.6(1)
**p3**	5731.4(3)	3.35(fixed)	3.80(9)
**p4**	2738.9(1)	3.35(fixed)	6.23(2)

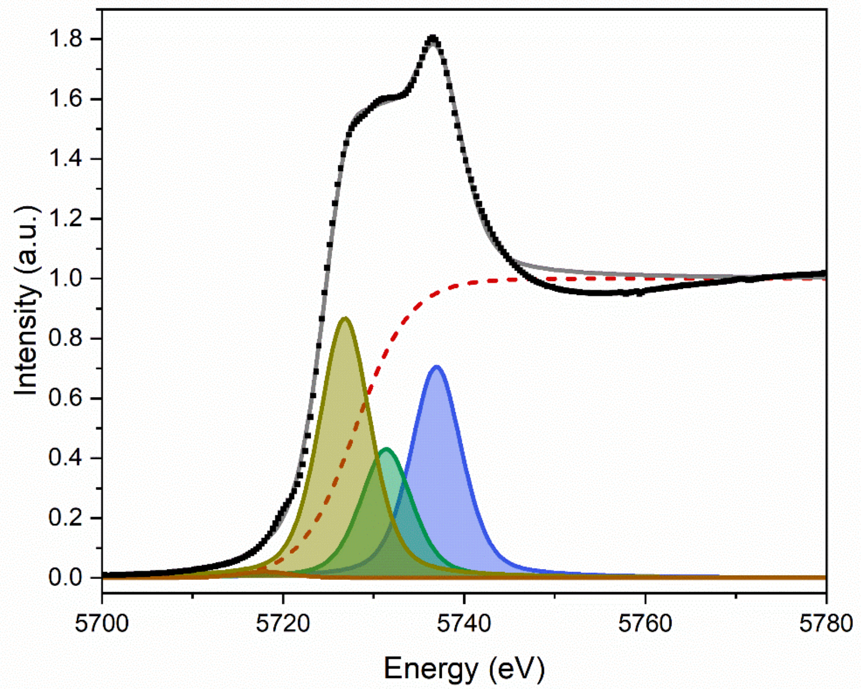

Figure S55: Cerium L_3-edge transmission XANES fit for **CeHAl**. Pre-edge: orange, p2: yellow, p3: green, p4: blue. The step is shown in red, the overall sum of the fits is shown as a solid grey trace, the experimental data is shown as a dotted black trace.

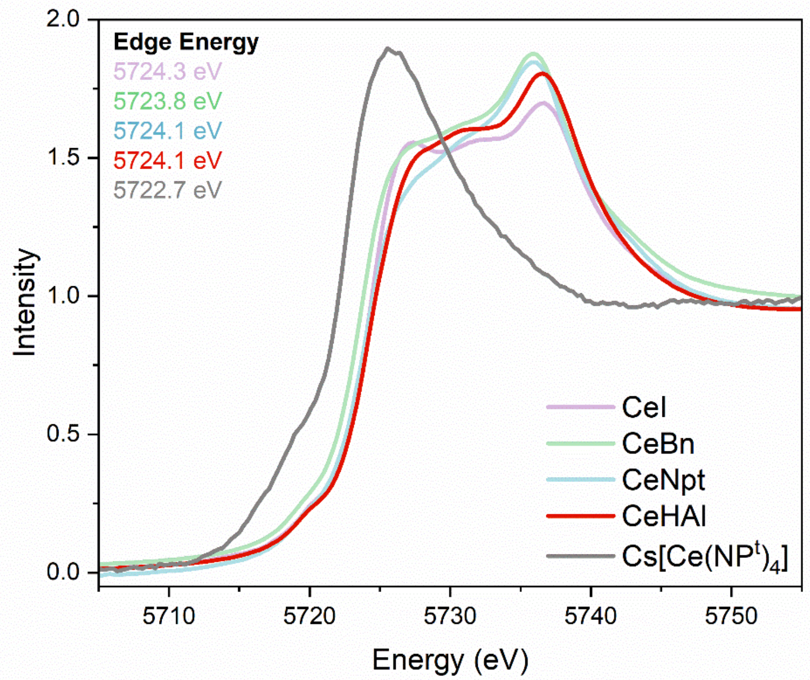

Figure S56: L_3-edge transmission XANES spectra of CeI(NP(^{t}Bu)$_3$)$_3$, CeBn(NP(^{t}Bu)$_3$)$_3$, CeNpt(NP(^{t}Bu)$_3$)$_3$ (abbreviated as CeI, CeBn, CeNpt respectively), and CsCe(NP(^{t}Bu)$_3$)$_4$. CsCe(NP(^{t}Bu)$_3$)$_4$ has a cerium edge energy that is -1.4 eV below the average tetravalent cerium edge energy. A smaller edge energy is expected for a trivalent system compared to a tetravalent

one. The feature at 5714 eV in the $CsCe(NP(^tBu)_3)_4$ (gray) trace is associated with the cesium L_1-edge.[11]

Single Crystal X-Ray Diffraction

Crystals suitable for X-ray diffraction were covered in Cargille-NVH oil in a N_2 glovebox and transferred to the diffractometer in a capped 20 mL vial. The crystals in NVH oil were transferred to a microscope slide and were mounted on a MiTeGen or nylon loop under a microscope. The loop was placed onto a Bruker D8 VENTURE diffractometer that was pre-cooled to 100 K. All data was collected at 100(2) K throughout the collection.

The asymmetric unit for **CeHAl** contains 2 molecules and 2 pentane; no other suitable space group was found to reduce the Z'. Hydride atoms were located on the electron difference map and refined freely. After their approximate location converged, a riding command was added (riding on Al). The hydride U_{iso} values were set to -1.2 as slight constraints on the ellipsoid size. The large ellipsoid is likely, in part, due to the relative location of the light atoms (H) directly bonded to a very heavy atom (Ce). This is a known difficulty associated with X-ray diffraction, as the phase problem has not been well resolved beyond krypton.

Crystals of **$Ce(NP(^tBu)_3)_2((NP(^tBu)_3)_2AlH_2)$** were multi-domain. The structure was solved using one of two domains present in the mounted crystal; a merged dataset was used (HKLF4). The R_{int} was taken from the .abs file. Additionally, two ether molecules were found using the SQUEEZE function in Olex2. The ether molecules were not clear in the difference map and could not be solved without using the SQUEEZE function.

Table S3: SC-XRD Crystallographic Data for reported structures.

	**CeHAl**	**$CeOPh_2$**	**$Ce(NP(^tBu)_3)_2((NP(^tBu)_3)_2AlH_2)$**
Empirical formula	$C_{51}H_{123}AlCeN_3P_3Si_3$	$C_{49}H_{92}CeN_3OP_3$	$C_{56}H_{130}AlCeN_4O_2P_4$
Formula weight	1122.80	972.28	1182.61
Temperature [K]	100.15	100.00	100.00
Crystal system	monoclinic	triclinic	triclinic
Space group (number)	$P2_1/c$ (14)	$P\overline{1}$ (2)	$P\overline{1}$ (2)
a [Å]	12.924(3)	11.4478(6)	12.604(3)
b [Å]	44.247(18)	13.0582(7)	13.065(3)
c [Å]	23.201(9)	19.0311(9)	21.042(5)
α [°]	90	96.433(2)	86.244(7)
β [°]	93.616(11)	93.469(2)	88.952(6)
γ [°]	90	109.013(2)	64.439(5)
Volume [$Å^3$]	13241(8)	2658.5(2)	3119.0(13)
Z	8	2	2
ρ_{calc} [gcm^{-3}]	1.126	1.215	1.259
μ [mm^{-1}]	0.859	0.981	0.887
F(000)	4864	1036	1282
Crystal size [mm^3]	0.1×0.13×0.369	0.146×0.2×0.304	0.362×0.561×0.612
2θ range [°]	3.82 to 52.81 (0.80 Å)	4.22 to 56.89 (0.75 Å)	4.15 to 52.97 (0.80 Å)
Index ranges	−16 ≤ h ≤ 13, −55 ≤ k ≤ 48, −28 ≤ l ≤ 29	−15 ≤ h ≤ 15, −17 ≤ k ≤ 17, −25 ≤ l ≤ 25	−15 ≤ h ≤ 15, −16 ≤ k ≤ 16, 0 ≤ l ≤ 26
Reflections collected	210182	218301	12241
Independent reflections	27085 [R_{int} = 0.1491, R_{sigma} = 0.1010]	13335 [R_{int} = 0.0533, R_{sigma} = 0.0219]	12239 [R_{int} = 0.0912, R_{sigma} = 0.0289]
Data / Restraints / Parameters	27085 / 0 / 1193	13335 / 24 / 582	12239 / 0 / 567
Goodness-of-fit on F^2	1.033	1.093	1.083
Final R indexes [I≥2σ(I)]	R_1 = 0.0518, wR_2 = 0.0870	R_1 = 0.0338, wR_2 = 0.0748	R_1 = 0.0407, wR_2 = 0.1087
Final R indexes [all data]	R_1 = 0.1173, wR_2 = 0.1117	R_1 = 0.0420, wR_2 = 0.0846	R_1 = 0.0464, wR_2 = 0.1137
Largest peak/hole [$eÅ^{-3}$]	1.30/−1.31	1.75/−0.74	1.22/−0.85
CCDC Number	2553428	2553429	2553430

Table S4: Relevant average bond metrics for tetravalent cerium complexes supported by imidophosphorane ligands.

	Average Distance (Å)		Average Angle (°)
	Ce–N	N–P	Ce–N–P
**CeHAl**	2.122(7)	1.576(5)	167(3)
**$CeOPh_2$**	2.151(14)	1.564(2)	160(3)
**Published Heteroleptic Tetravalent Cerium Complexes**			
**$CeI(NP(^tBu)_3)_3$** [2]	2.11(2)	1.58(2)	160(1)
**$CeBn(NP(^tBu)_3)_3$** [2]	2.142(7)	1.57(1)	167(5)
**$CeNpt(NP(^tBu)_3)_3$** [2]	2.147(6)	1.566(4)	162.1(7)
**Published Homoleptic Tetravalent Cerium Complexes**			
**$Ce(NP(^tBu)_3)_4$** [11]	2.176(4)	1.562(3)	Not Reported
**$Ce(NP^*)_4$** [21]	2.237(1)	1.557(2)	163.0(1)
**$Ce(NP(pip)_3)_4$** [21,22]	2.20(2)	1.42(4)	166.9(1)
**$Ce(NPC)_4$** [23]	2.160(6)	1.539(1)	157.3(7)

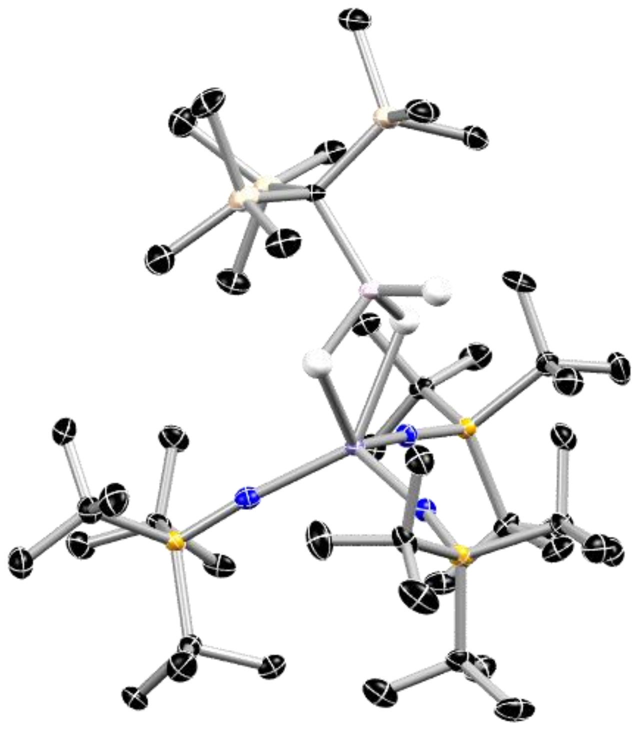

Figure S57: Molecular structure of **CeHAl** with thermal ellipsoids shown at 50% probability. H atoms, except for hydrides, are omitted for clarity. The asymmetric unit contains 2 **CeHAl** and 2 pentane molecules, they have been omitted for clarity. Al shown in in light pink, Ce shown in purple, C shown in black, H shown in white, N shown in blue, P shown in orange, Si shown in beige.

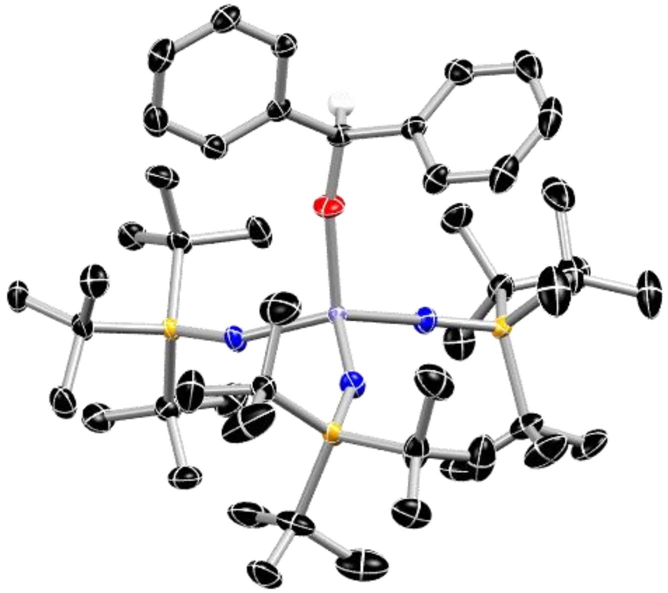

Figure S58: Molecular structure of **CeOPh₂** with thermal ellipsoids shown at 50% probability. H atoms, except for the hydrogen on C1, are omitted for clarity. C shown in black, Ce shown in purple, H shown in white, N shown in blue, O shown in red, P shown in orange.

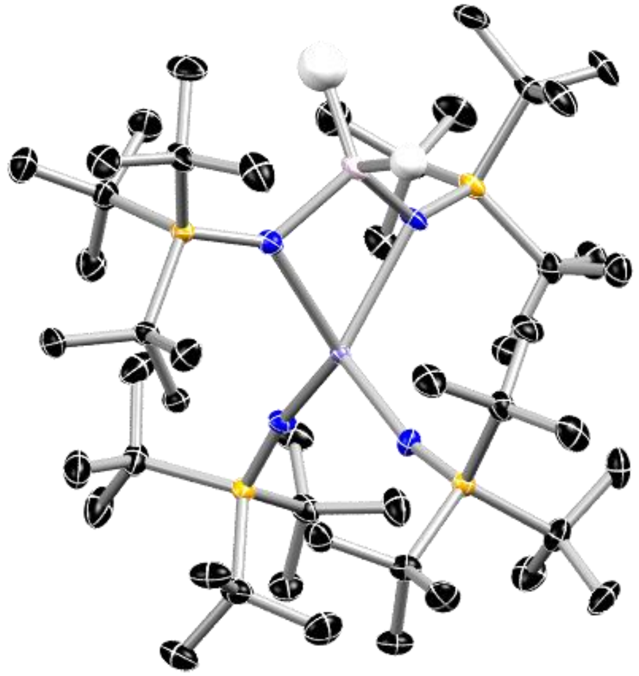

Figure S59: Molecular structure of **$Ce(NP(^tBu)_3)_2((NP(^tBu)_3)_2AlH_2)$** with thermal ellipsoids shown at 50% probability. H atoms, except for hydrides, are omitted for clarity. Al shown in in light pink, C shown in black, Ce shown in purple, H shown in white, N shown in blue, P shown in orange.

Table S5: Additional Bond Metrics for **$CeOPh_2$**.

Bond Length (Å)		Bond Angle (°)	
**Ce–O**	2.130(2)	**Ce–O–C**	167.3(2)
**O–C**	1.393(3)	**O–C–C_{Ph} (average)**	110.8(2)

Computational Details

Geometry Optimizations of **CeHAl** were performed using Density Functional Theory (DFT) as implemented in the Turbomole program package v7.8.[24,25] For all atoms, the def2-TZVP basis set was used along with Grimme's D3 dispersion correction.[26,27] The Cartesian gradient was converged to 1×10^{-4} a.u., the SCF energy was converged to 1×10^{-7} a.u., and the grid size was set to m4.[28] The resolution of identity (RI) approximation was employed for integral evaluation.[29] These structures were confirmed to be minima via harmonic vibrational analysis. Functional testing (Tables S6-S7) primarily resulted in κ^2 structures, in line with the crystal structure, however, PBE (with no dispersion corrections) yielded a κ^3 structure (Figure S60).[27,30-38] Therefore, the CAM-B3LYP-D3 optimized structure (**CeHAl-κ^2**) was used for its excellent Ce-Al and Al-C distances resulting in the κ^2 binding seen in the crystal structure. Neglecting dispersion corrections did result in the formation of a κ^3 structure (**CeHAl-κ^3**), also found to be at a minimum. For the κ^3 structure, the PBE geometry was reoptimized with CAM-B3LYP-D3 and several constraints for further analysis, facilitating comparisons between the two coordination modes.

Kohn-Sham density functional calculations were performed on **CeHAl-κ^2** with the 2024 release of Amsterdam Modeling Suite (AMS).[39] The computations of the NMR shielding constants (σ) were performed with the NMR module[40,41] in the Amsterdam Density Functional (ADF) package, using both the scalar relativistic (SR) and spin-orbit (SO) zero order regular approximation (ZORA) Hamiltonian.[42-44] The functional used for the NMR calculations was the generalized gradient approximation (GGA) functional Perdew-Burke-Ernzerhof (PBE).[30-33] The all-electron Slater-type atomic orbital (STO) basis sets of triple-ζ doubly polarized (TZ2P) quality were used for Ce, Al, N, P and each hydride with no frozen core.[45] The TZP basis was used for the remaining C, Si, and H atoms.[45] The conductor-like screening model (COSMO) was used to describe solvent effects (toluene).[46] To quantify the composition of chemical bonds of interest, natural localized molecular orbitals (NLMOs)[47] were attained with the NBO 7.0 program as interfaced with AMS.[48,49] ^{1}H NMR chemical shifts (δ) are computed taking tetramethylsilane (TMS) as the reference. The shielding (σ) from **CeHAl** is subtracted from the reference in accordance with Equation 1.

$$\delta_{probe} = \sigma_{ref} - \sigma_{probe} \quad (1)$$

Quantum Theory of Atoms in Molecules (QTAIM)[50,51] and Energy Decomposition Analysis (EDA),[52-55] as implemented in ADF 2024, was performed on **CeHAl** based on the PBE density utilizing the TZ2P basis set. For EDA, Grimme's D3 dispersion corrections are also employed; the anionic fragment included aluminum along with all three hydrides and the $-C(TMS)_3$ backbone. The cationic fragment contained the remaining molecule, including the Ce metal. Both fragments and the neutral complex are modeled as closed-shell singlet states.

Time-dependent density functional theory (TD-DFT) calculations were performed on **CeHAl** to elucidate the UV-Vis-NIR spectra. The CAM-B3LYP functional along with Grimme's D3 dispersion corrections as implemented in ORCA 6.1.1.[56,57] The SARC-ZORA-TZVP basis is used for Ce while the ZORA-def2-TZVP basis is used for all other atoms.[58-60] The resolution of the identity chain of spheres (RIJCOSX) approximation is employed.[61-63] In TD-DFT, 100 roots were computed, and natural transition orbitals were attained. Input and output files available in the following figshare: 10.6084/m9.figshare.32245104.

Table S6. Interatomic distances of interest for **CeHAl** using several DFT functionals.

Bond	Exp	PBE	PBE0	HSE06	B3LYP	CAM-B3LYP
Ce-Al	3.27(3)	3.036	3.245	3.247	3.296	3.277
Ce-N1	2.121(3)	2.162	2.125	2.129	2.155	2.132
Ce-N2	2.127(6)	2.167	2.116	2.120	2.144	2.121
Ce-N3	2.117(8)	2.164	2.125	2.129	2.154	2.132
N-P1	1.574(8)	1.585	1.571	1.571	1.575	1.565
N-P2	1.576(5)	1.586	1.573	1.573	1.577	1.567
N-P3	1.579(4)	1.586	1.571	1.571	1.575	1.565
Ce-H1	2.48(1)	2.618	2.476	2.476	2.525	2.494
Ce-H2	2.44(1)	2.603	2.463	2.462	2.500	2.483
Ce-H3	4.18(5)	2.634	3.866	3.876	3.901	3.962
Al-H1	1.63(1)	1.661	1.674	1.673	1.665	1.661
Al-H2	1.60(4)	1.661	1.672	1.672	1.665	1.659
Al-H3	1.56(1)	1.661	1.606	1.605	1.598	1.594
Al-C	2.009(1)	2.002	2.006	2.007	2.028	2.009

Table S7. Interatomic distances of interest for **CeHAl** using several DFT functionals with D3.

Bond	Exp	PBE-D3	PBE0-D3	HSE06-D3	B3LYP-D3	CAM-B3LYP-D3
Ce-Al	3.27(3)	3.277	3.256	3.258	3.282	3.272
Ce-N1	2.121(3)	2.149	2.114	2.124	2.131	2.117
Ce-N2	2.127(6)	2.141	2.108	2.113	2.126	2.112
Ce-N3	2.117(8)	2.150	2.116	2.123	2.135	2.122
N-P1	1.574(8)	1.583	1.567	1.568	1.568	1.560
N-P2	1.576(5)	1.586	1.570	1.571	1.571	1.564
N-P3	1.579(4)	1.584	1.568	1.569	1.569	1.561
Ce-H1	2.48(1)	2.439	2.419	2.420	2.440	2.437
Ce-H2	2.44(1)	2.496	2.478	2.472	2.513	2.498
Ce-H3	4.18(5)	4.247	4.218	4.206	4.254	4.245
Al-H1	1.63(1)	1.679	1.672	1.673	1.661	1.658
Al-H2	1.60(4)	1.670	1.661	1.663	1.650	1.648
Al-H3	1.56(1)	1.612	1.601	1.601	1.592	1.589
Al-C	2.009(1)	2.015	1.999	2.005	2.012	1.999

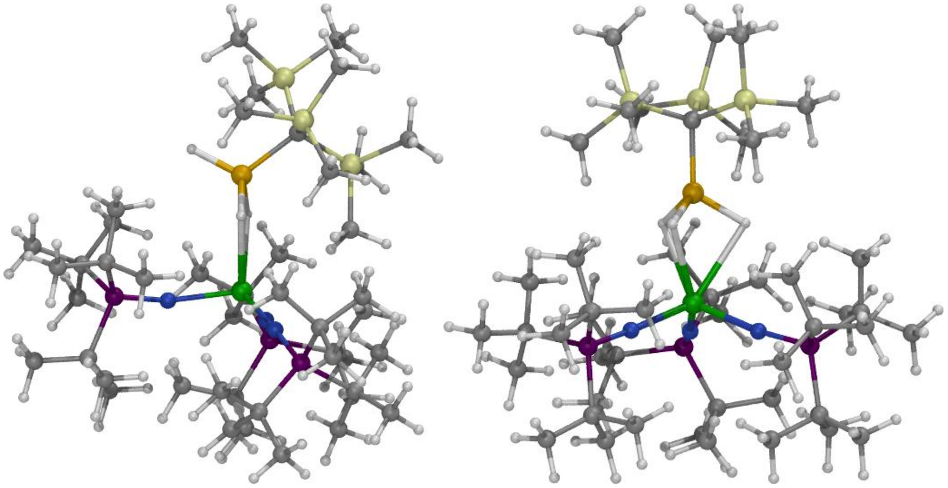

Figure S60. DFT optimized structure **CeHAl-κ²** (left) and **CeHAl-κ³** (right) from the CAM-B3LYP-D3 and PBE functionals, respectively. The following nonstandard colors were used: Ce (green), Al (orange), P (purple), and Si (yellow).

Table S8. Relative CAM-B3LYP-D3 energies (kcal/mol) on optimized geometries. A) CAM-B3-LYP-D3 (k^2) optimized geometry. B) Single point on PBE optimized geometry. C) Al-C bond length constrained optimization of **B**. D) Al-κ^3-H constrained optimization of **C**.

**A**	0.00
**B**	15.7
**C**	2.5
**D**	2.3

Table S9. Selected Bond Lengths and/or Interatomic Distances and the respective Mayer, Gophinatan-Jug, and Nalewajski-Mrozek (N-M) bond orders, variants 1-3, for the optimized geometry of **CeHAl** based on the PBE density.

Atoms	Distance	Mayer	G-J	N-M1	N-M2	N-M3
Ce-Al	3.2720	0.1074	0.1930	0.2096	-0.0909	0.1859
Ce-H1	2.4371	0.1559	0.2077	0.2321	0.3741	0.2426
Ce-H2	2.4984	0.1521	0.1879	0.2100	0.3524	0.2193
Ce-N1	2.1171	1.1065	1.3886	1.6124	1.9835	1.7059
Ce-N2	2.1119	1.1339	1.4112	1.6376	2.0030	1.7307
Ce-N3	2.1218	1.1032	1.3820	1.6051	1.9776	1.6989
Al-H1	1.6581	0.6214	0.6799	0.6926	0.7487	0.7312
Al-H2	1.6477	0.6345	0.6992	0.7123	0.7670	0.7514
Al-H3	1.5891	0.8251	0.8956	0.9144	0.9607	0.9673
Al-C	1.9987	0.7022	0.7307	0.7705	1.0698	0.8315

Table S10. QTAIM (3, -1) bond critical points (BCPs) and (3, +1) ring critical points (RCPs) located between atom centers listed in column 1 for **CeHAl**. Topological analysis is based on the PBE density computed in ADF. Delocalization indices (δ_{DI}) computed for each atom pair.

Bond	Type	ρ	$\nabla^2\rho$	Elip.	V(r)	G(r)	E(r)	δ_{DI}
Ce-Al	RCP	0.02187	0.049949	-1.78271	-0.01398	0.013236	-0.00075	0.0378
Ce-H1	BCP	0.02873	0.060262	0.22587	-0.02050	0.017782	-0.00272	0.2358
Ce-H2	BCP	0.03216	0.064957	0.18853	-0.02409	0.020163	-0.00392	0.2124
Al-H1	BCP	0.06792	0.159295	0.05906	-0.07821	0.059015	-0.01919	0.2841
Al-H2	BCP	0.06986	0.162727	0.05230	-0.08161	0.061145	-0.02046	0.2951
Al-H3	BCP	0.08164	0.184605	0.00890	-0.10362	0.074885	-0.02873	0.3806

Table S11. Energy Decomposition Analysis (EDA) using the PBE functional on the optimized geometry of **CeHAl**. Energies in kcals/mol.

	INT E	Pauli	Elect	Orb	Disp	%Elec	%Orb	%Disp
**1A**	-104.99	58.75	-103.49	-43.93	-16.32	63.2	26.8	10.0

Table S12. ETS-NOCV analysis results for the interaction of the Al containing ligand with the remaining molecule (PBE-D3 functional). NOCV pair orbital interaction energies are given for the two strongest interactions (others smaller than 5 kcal/mol). For each pair, the NOCV eigenvalue, $|v_e|$, which corresponds to the number of electrons transferred is given in parentheses. The total of all orbital interaction energies (kcal/mol) is also reported.

	**CeHAl**
NOCV1	-12.7 (0.41)
NOCV2	-9.8 (0.32)
Total	**-43.69**

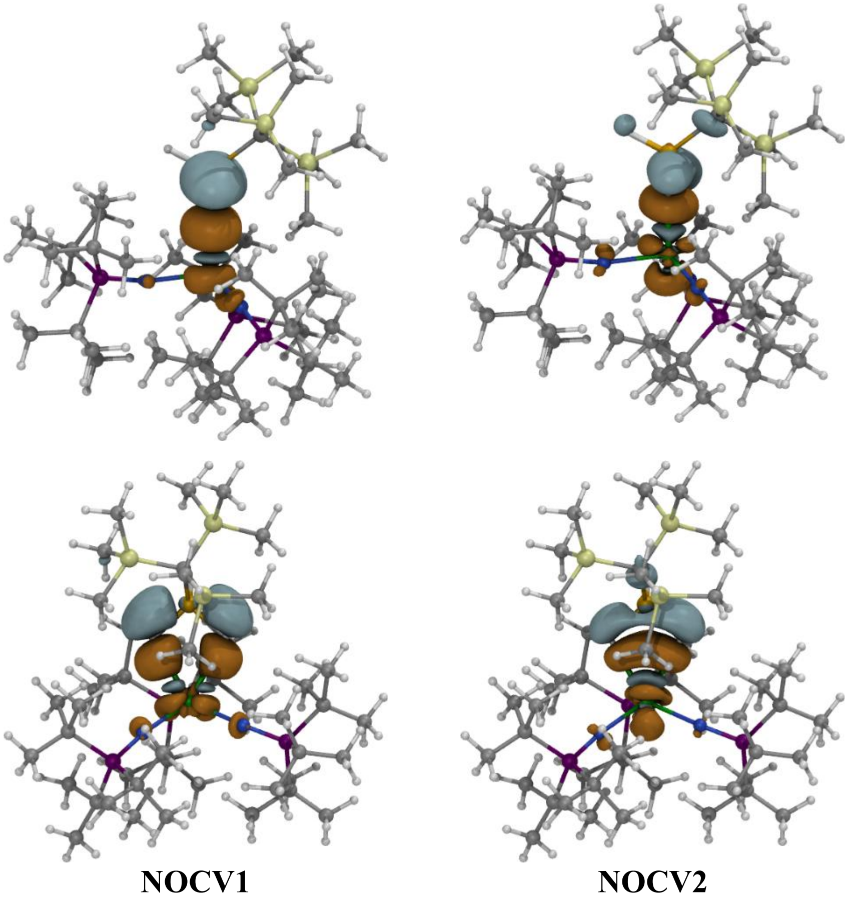

Figure S61. ETS-NOCV deformation densities based on the PBE density computed in AMS2024. Plotted with an isosurface of 0.001 a.u. Blue and orange regions represent areas of charge depletion and accumulation, respectively. NOCV1 and NOCV2 are shown in two different orientations in the top and bottom.

Computed Spectra

Table S13. Nuclear Magnetic Resonance shielding constants (σ) and chemical shifts (δ) relative to tetramethylsilane (TMS) at the scalar relativistic and spin-orbit (-SO) ZORA levels of theory with the PBE functional. Relativistic shifts (Δ_{SO}) computed as the difference between spin-orbit and scalar relativistic chemical shifts.

Complex	Atoms	Method	σ_{calc} (ppm)	δ_{calc} (ppm)	Δ_{SO} (ppm)	δ_{expt} (ppm)
**TMS**	H	PBE / PBE-SO	31.46 / 31.46	--	0.001	0
**CeHAl** (κ^2)	H1	PBE / PBE-SO	26.44 / 23.40	5.02 / 8.06	3.04	5.85
	H2	PBE / PBE-SO	26.39 / 23.52	5.06 / 7.94	2.88	
	H3	PBE / PBE-SO	27.02 / 26.56	4.44 / 4.90	0.46	
	H_{avg}	PBE / PBE-SO	26.62 / 24.49	4.84 / 6.97	2.12	
**CeHAl** (κ^3)	H1	PBE / PBE-SO	26.81 / 24.69	4.65 / 6.77	2.12	5.85
	H2	PBE / PBE-SO	26.82 / 24.47	4.64 / 6.99	2.35	
	H3	PBE / PBE-SO	26.75 / 24.75	4.71 / 6.71	2.00	
	H_{avg}	PBE / PBE-SO	26.79 / 24.64	4.67 / 6.82	2.15	
κ^2 & κ^3	H_{avg}	PBE / PBE-SO	26.70 / 24.57	4.75 / 6.89	2.14	5.85

121 **122** **123**

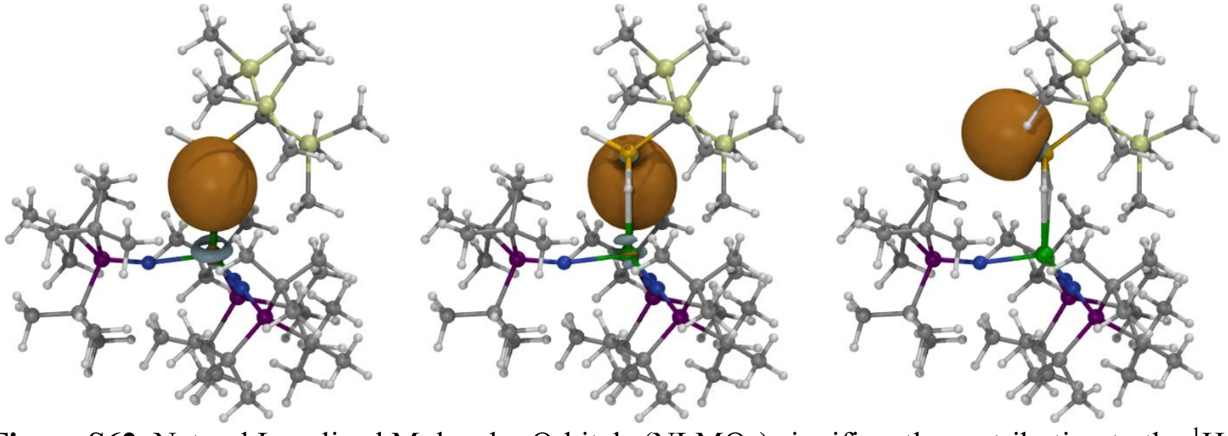

Figure S62. Natural Localized Molecular Orbitals (NLMOs) significantly contributing to the ^{1}H NMR shielding tensors of **CeHAl-κ²**. NLMOs attained at the PBE (toluene) level of theory plotted with an isosurface value of 0.04 a.u.

Table S14. NLMO Analysis in the Natural Atomic Orbital (NAO) Basis of the three NLMOs contributing to the ^{1}H NMR shielding tensors of **CeHAl-κ^{2}** based on the PBE (toluene) density computed in ADF.

**NLMO**	**121**	**122**	**123**
**% Ce**	**5.786**	**5.556**	**0.950**
s	4.03	3.44	10.39
p	0.63	0.68	0.11
d	71.81	72.24	84.69
f	23.53	23.64	4.81
**% Al**	**25.179**	**25.810**	**32.303**
s	31.08	33.52	39.92
p	68.44	66.02	59.71
d	0.46	0.45	0.35
f	0.01	0.01	0.01
**% H**	**67.880**	**67.478**	**65.910**
s	99.58	99.54	99.49
p	0.41	0.45	0.51
d	0.01	0.01	0.01

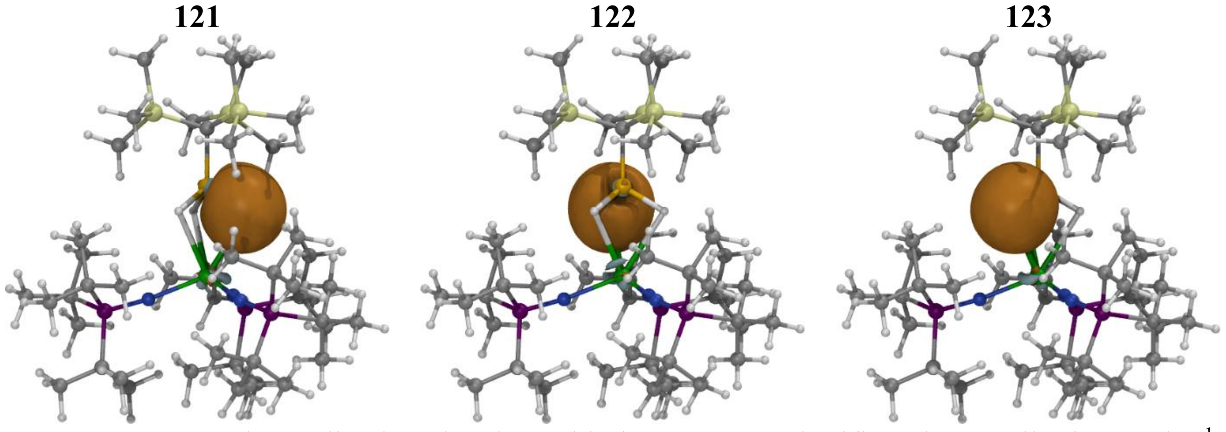

Figure S63. Natural Localized Molecular Orbitals (NLMOs) significantly contributing to the ^{1}H NMR shielding tensors of **CeHAl-κ^{3}**. NLMOs attained at the PBE (toluene) level of theory plotted with an isosurface value of 0.04 a.u.

Table S15. NLMO Analysis in the Natural Atomic Orbital (NAO) Basis of the three NLMOs contributing to the ^{1}H NMR shielding tensors of **CeHAl-κ³** based on the PBE (toluene) density computed in ADF.

NLMO	121	122	123
**% Ce**	**5.932**	**6.168**	**5.743**
s	2.77	2.67	2.89
p	0.34	0.37	0.35
d	75.20	74.29	76.01
f	21.70	22.67	20.75
**% Al**	**28.049**	**27.633**	**28.277**
s	31.93	32.09	32.71
p	67.61	67.45	66.84
d	0.44	0.44	0.43
f	0.02	0.02	0.02
**% H**	**64.863**	**65.082**	**28.277**
s	99.54	99.54	99.54
p	0.46	0.45	0.45
d	0.01	0.01	0.01

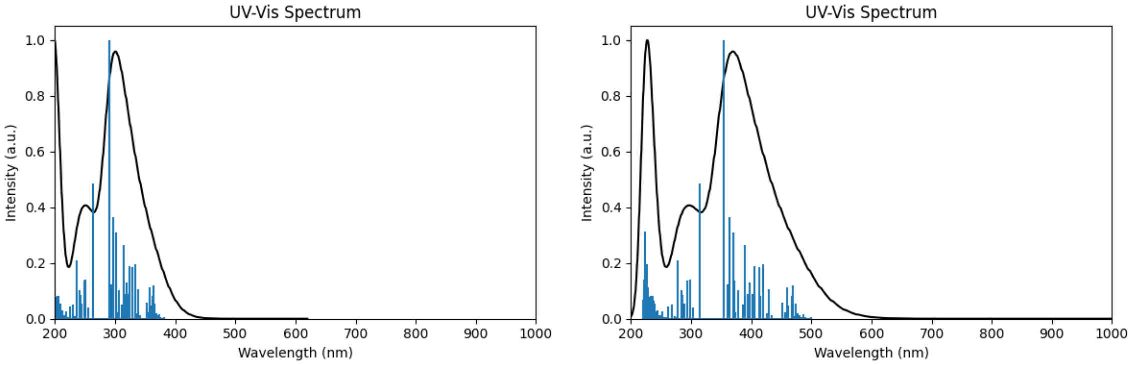

Figure S64. Normalized computed TD-DFT Spectra (blue) of the UV-Vis Spectra (CAM-B3LYP-D3) on **CeHAl-κ²**. Gaussian broadening with a FWHM of 0.5 eV (black). (left) Unshifted results. (right) To align with experiment, a 0.77 eV red shift is applied to the TDDFT results to align the experimental λ_{max} (370 nm) with the computed feature at 4.12 eV (301 nm).

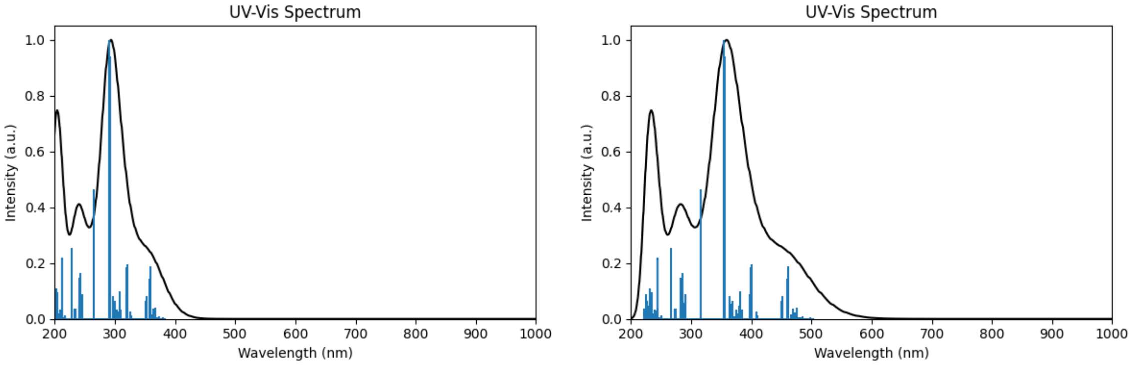

Figure S65. Normalized computed TD-DFT Spectra (blue) of the UV-Vis Spectra based on the CAM-B3LYP-D3 density on **CeHAl-κ³**. Gaussian broadening with a FWHM of 0.5 eV (black).

(left) Unshifted results. (right) To align with experiment, a 0.77 eV red shift is applied to the TDDFT results.

Table S16. Natural Transition Orbital Pairs with contributions to each state larger than 10% for selected states in the CAM-B3LYP-D3 TD-DFT UV-Vis Spectra of **CeHAl-κ²** (Figure S64). Each donor (left) and acceptor (right) orbitals plotted with an isosurface value of 0.04 a.u. Energies redshifted by 0.77 eV to align with experiment.

State 15: 2.638 eV (470.1 nm)		
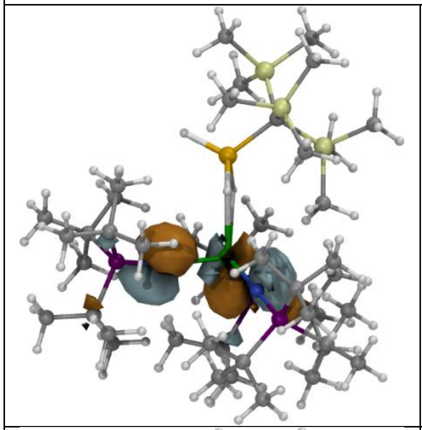	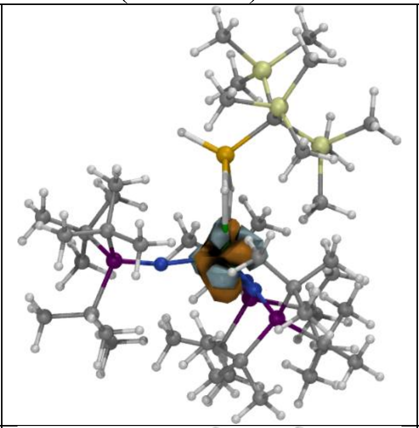	51.8%
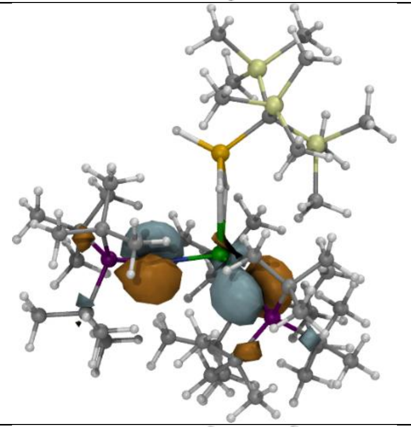	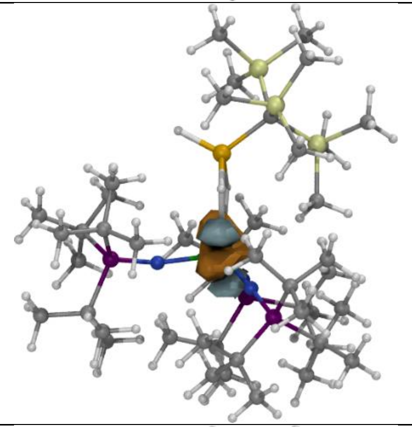	28.3%
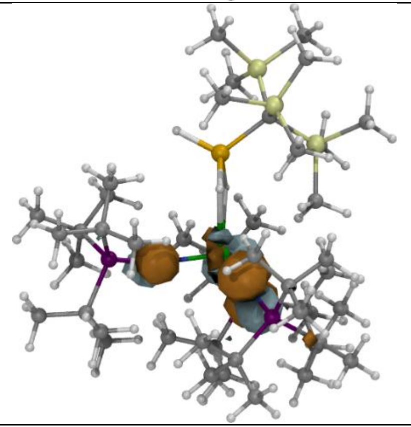	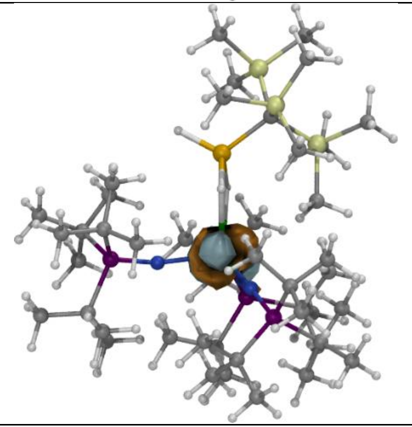	14.9%
State 41: 3.494 eV (354.9 nm)		

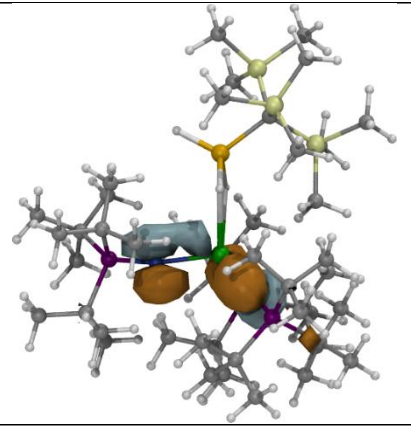	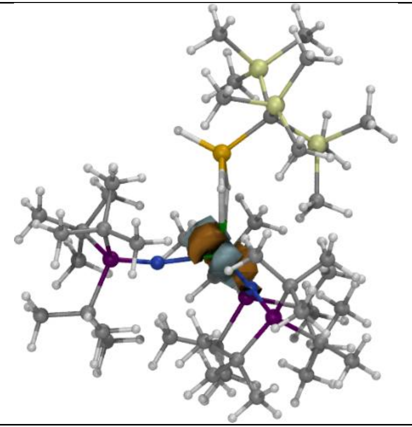	88.1%
State 42: 3.943 eV (314.5 nm)		
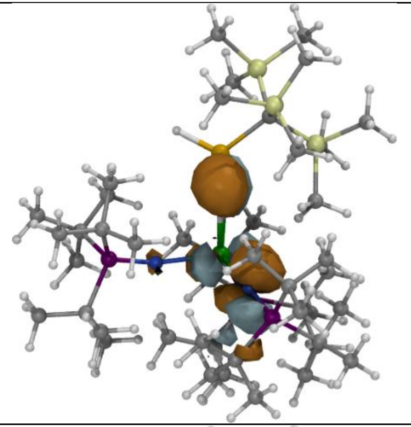	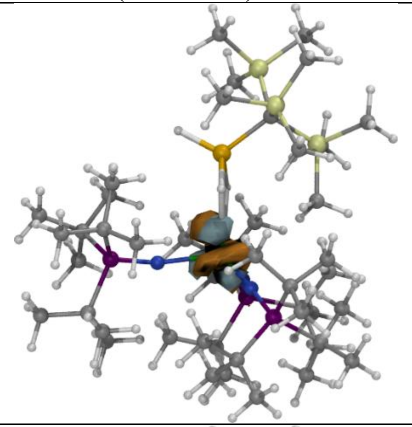	42.8%
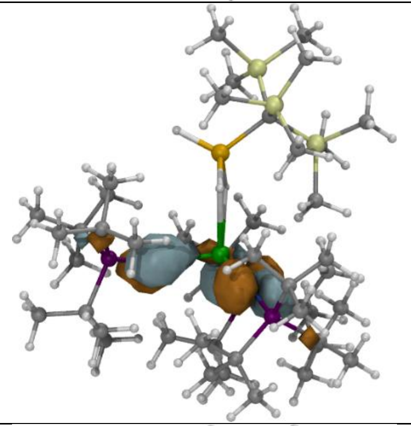	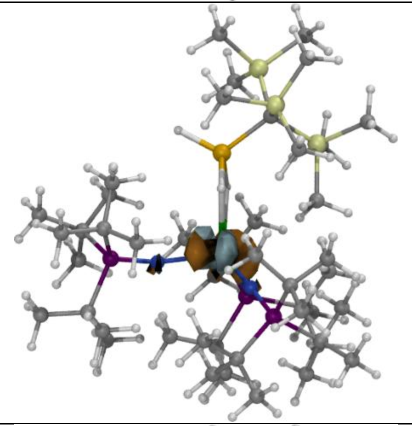	16.5%
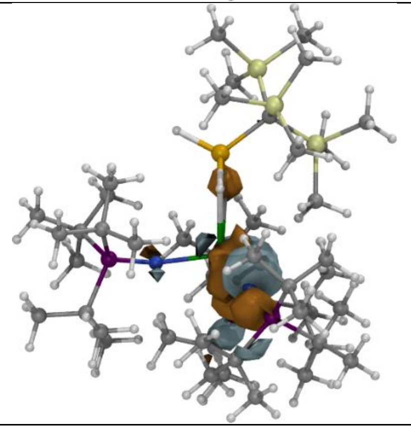	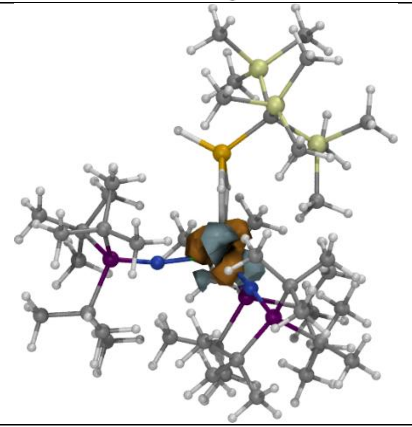	12.9%

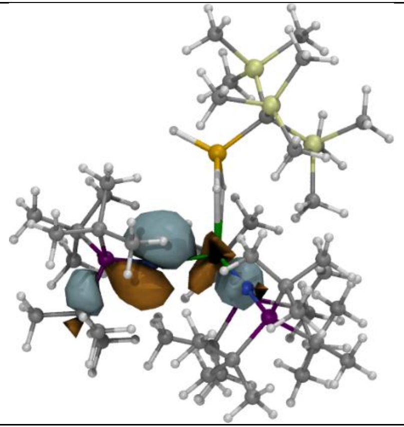	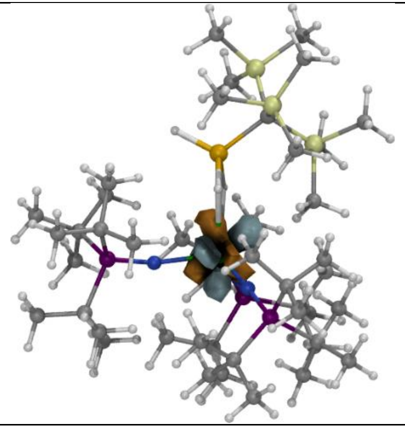	10.0%
State 50: 4.462 eV (277.9 nm)		
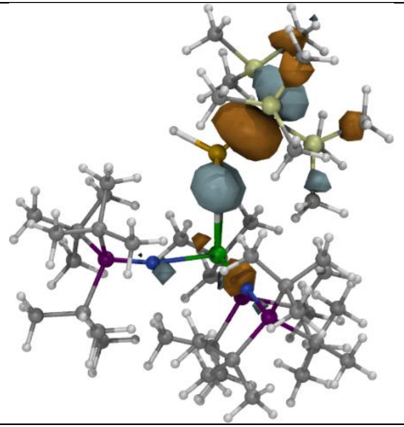	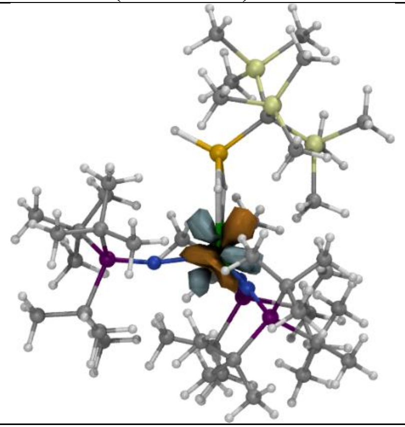	97.9%
State 92: 5.461 eV (227.1 nm)		
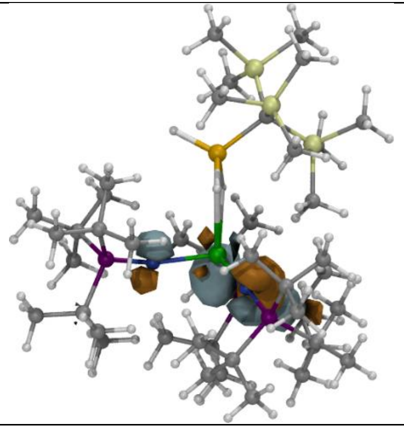	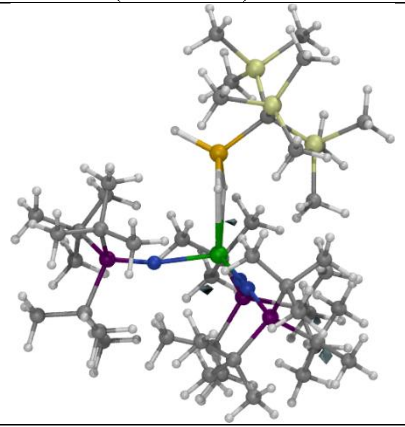	55.1%

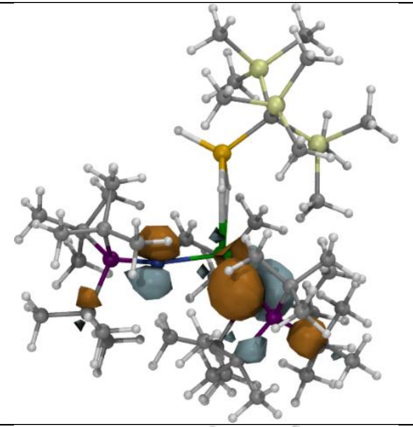	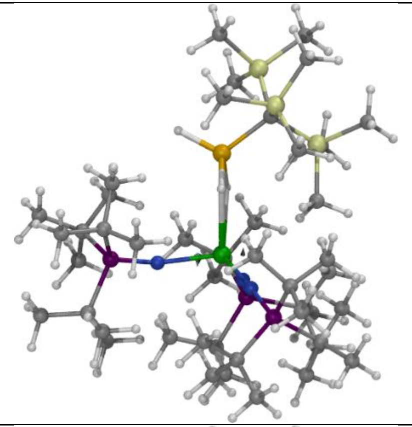	27.6%
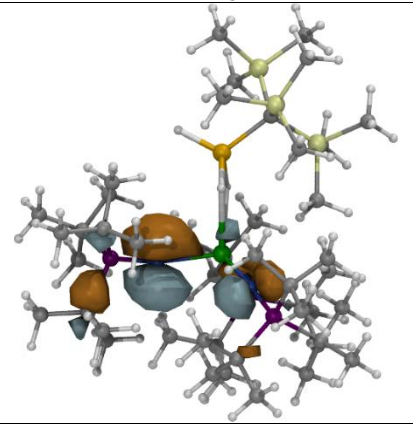	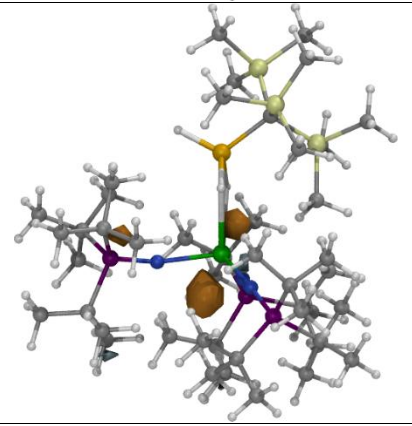	10.2%
State 95: 5.545 eV (223.6 nm)		
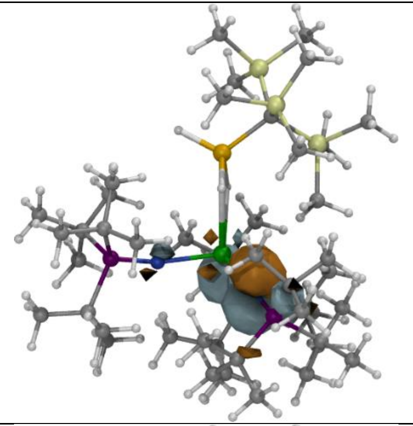	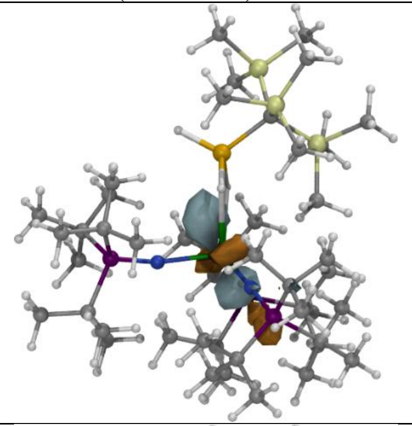	62.0%
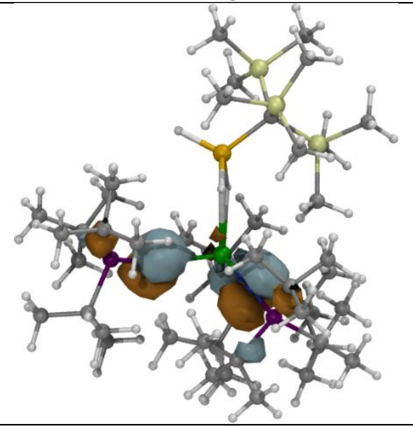	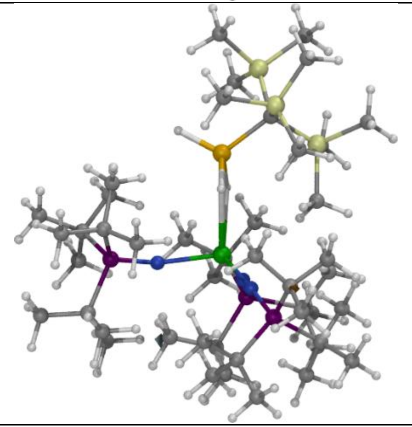	17.6%

Table S17. Natural Transition Orbital Pairs with contributions to each state larger than 10% for selected states in the CAM-B3LYP-D3 TD-DFT UV-Vis Spectra of **CeHAl-κ³** (Figure S65). Each donor (left) and acceptor (right) orbitals plotted with an isosurface value of 0.04 a.u. Energies redshifted by 0.77 eV to align with experiment.

State 15: 2.660 eV (466.2 nm)		
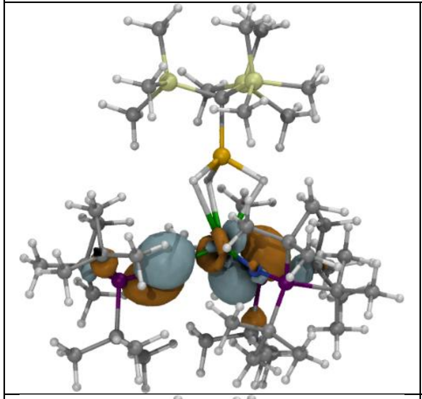	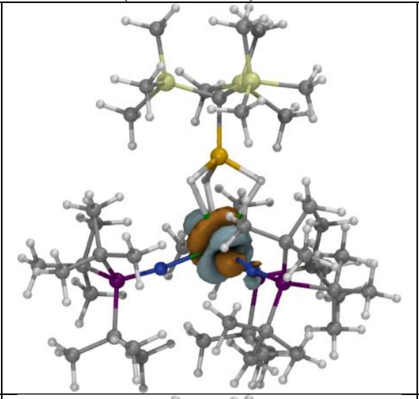	72.6%
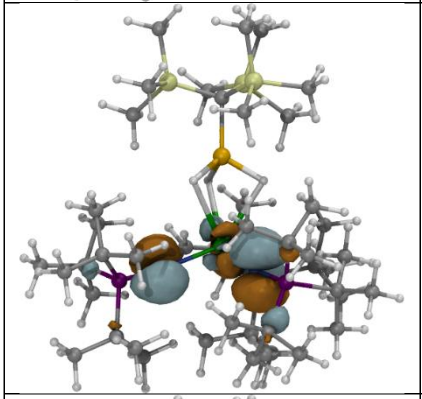	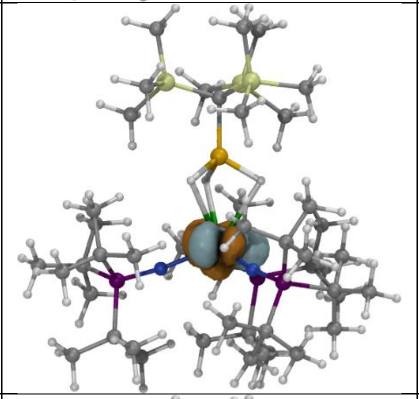	20.3%
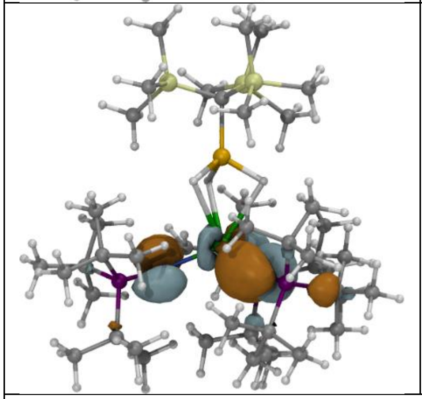	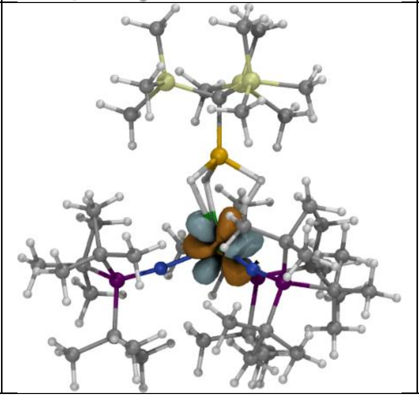	6.3%
State 41: 3.499 eV (354.4 nm)		

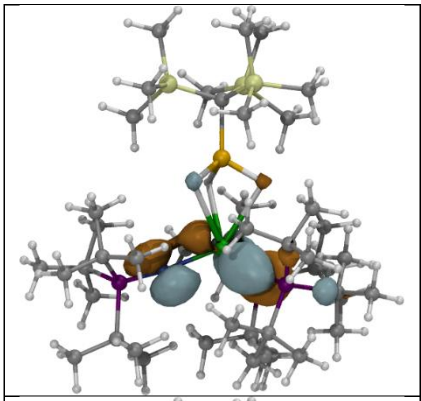	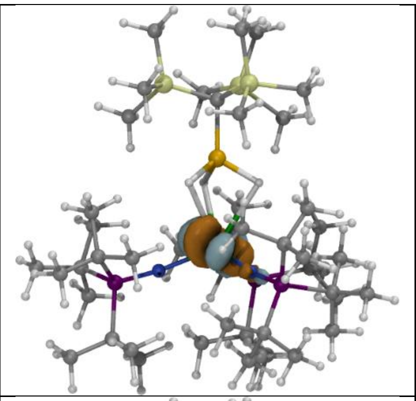	71.6%
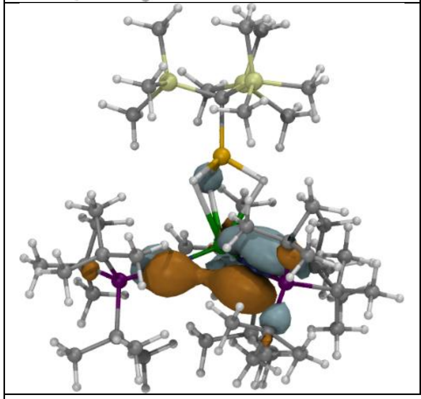	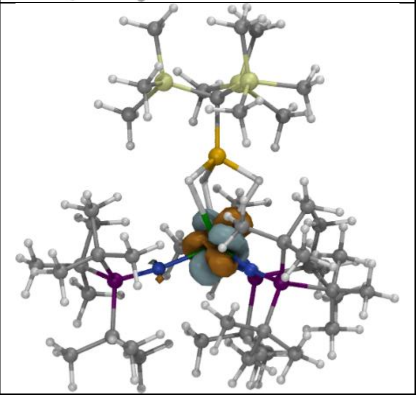	22.0%
State 42: 3.922 eV (316.2 nm)		
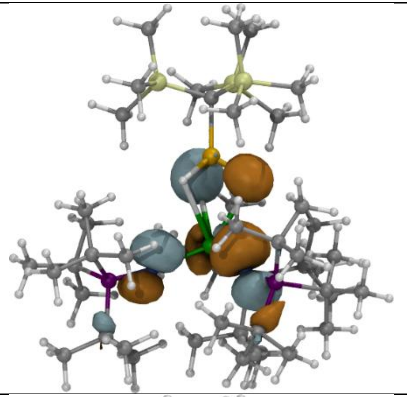	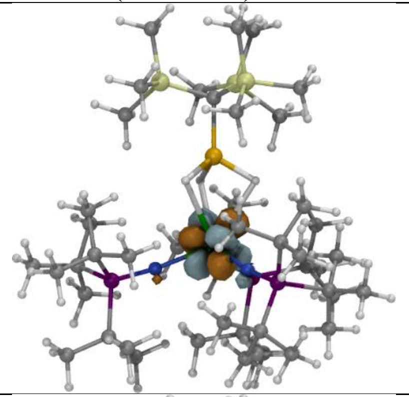	26.9%
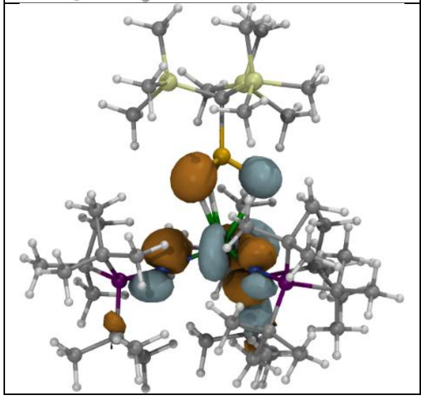	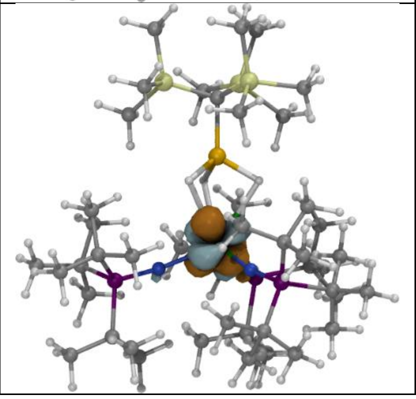	22.8%

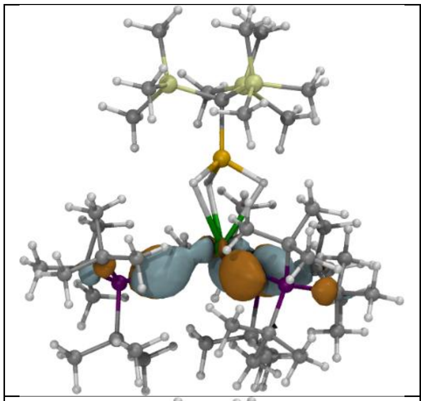	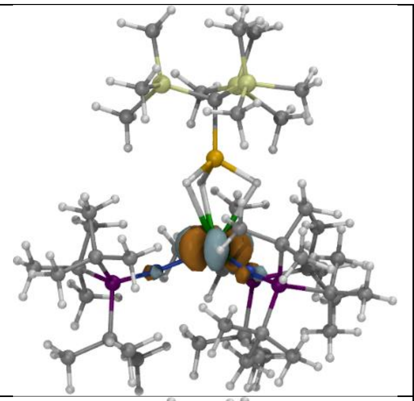	18.8%
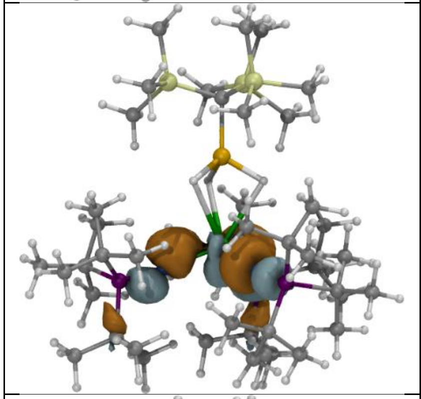	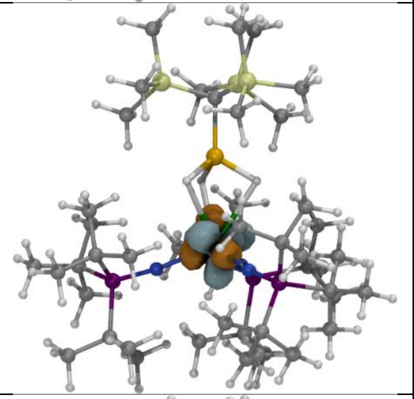	11.6%
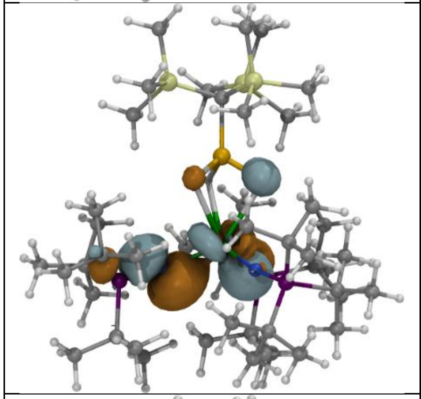	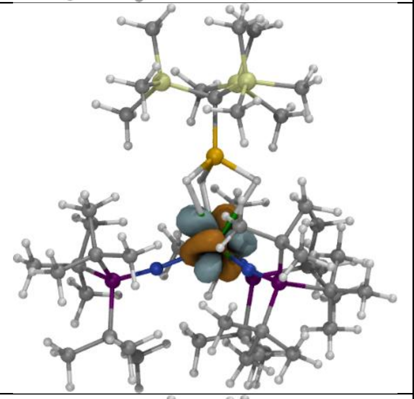	6.9%
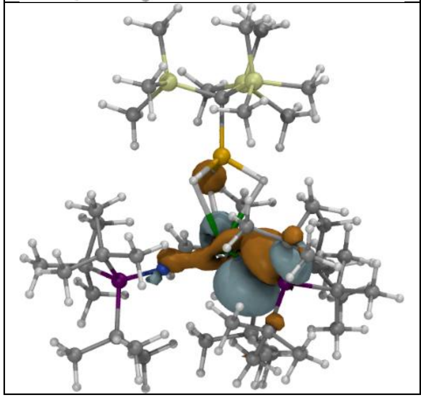	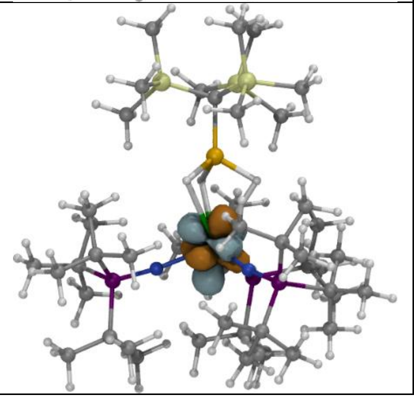	6.1%

<table>
<tr><td>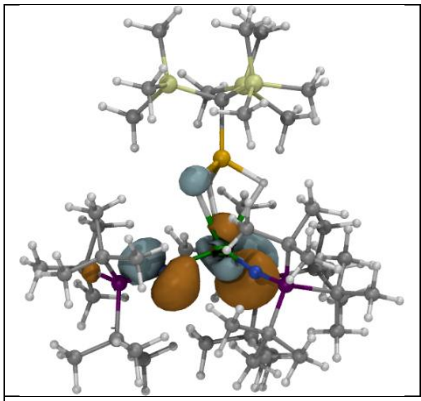</td><td>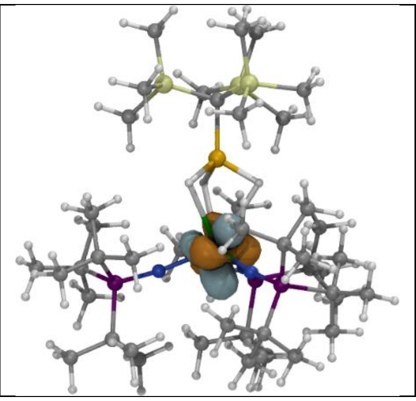</td><td>5.3%</td></tr>
<tr><td colspan="3">State 50: 4.383 eV (282.9 nm)</td></tr>
<tr><td>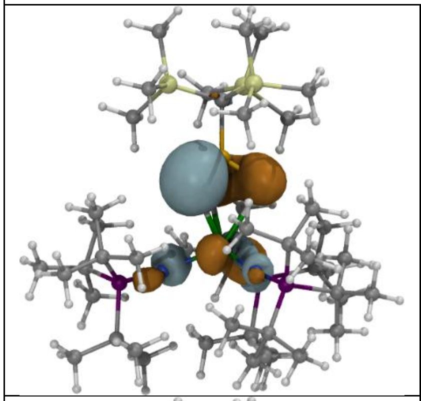</td><td>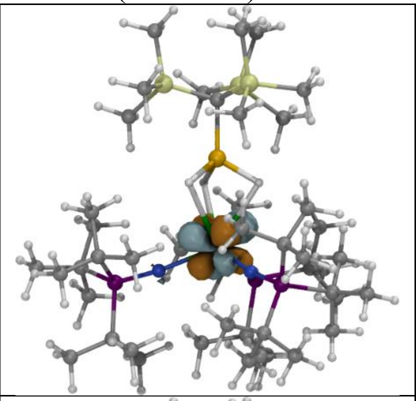</td><td>93.4%</td></tr>
<tr><td>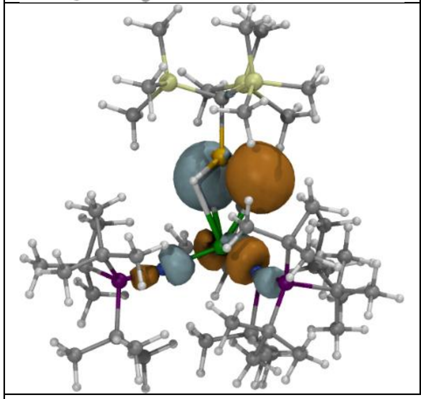</td><td>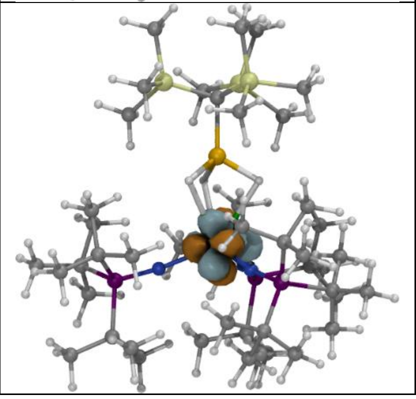</td><td>5.9%</td></tr>
<tr><td colspan="3">State 92: 5.445 eV (227.7 nm)</td></tr>
<tr><td>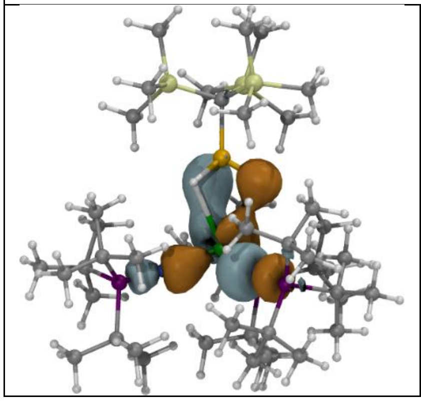</td><td>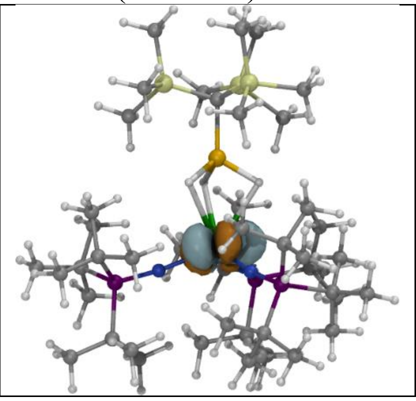</td><td>47.2%</td></tr>
</table>

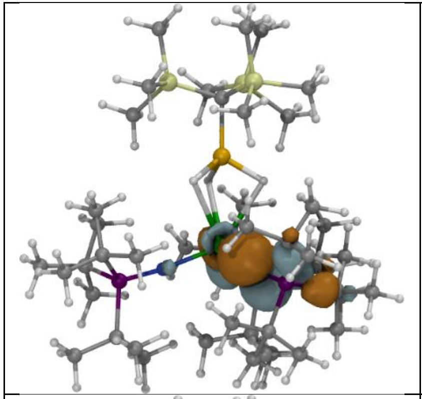	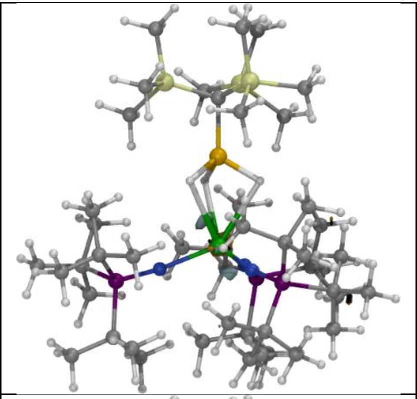	20.6%
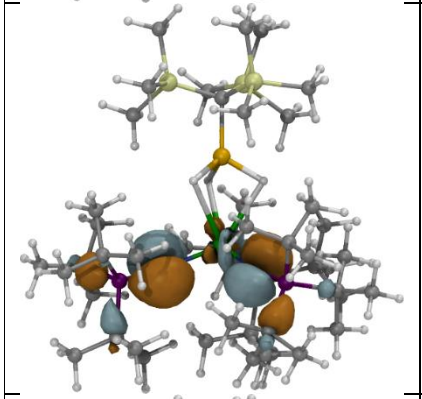	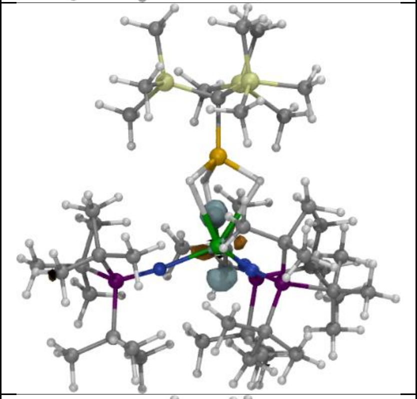	16.3%
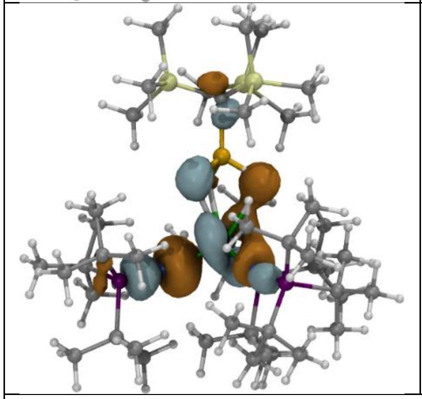	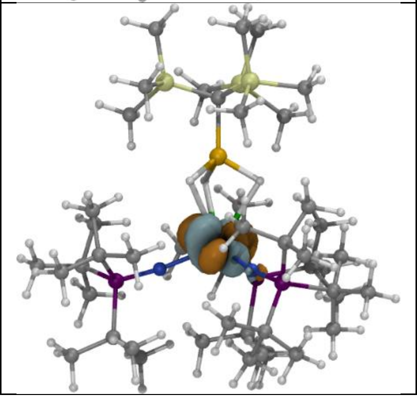	7.8%
State 95: 5.504 eV (225.3 nm)		
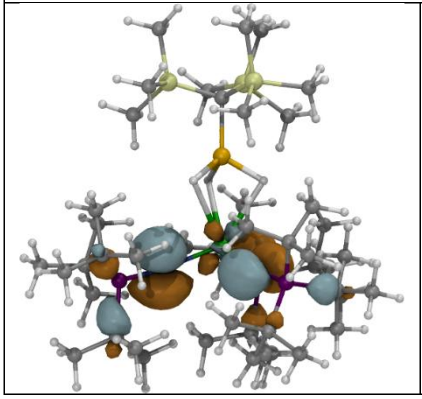	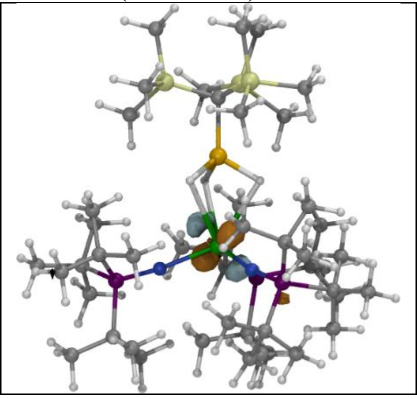	43.7%

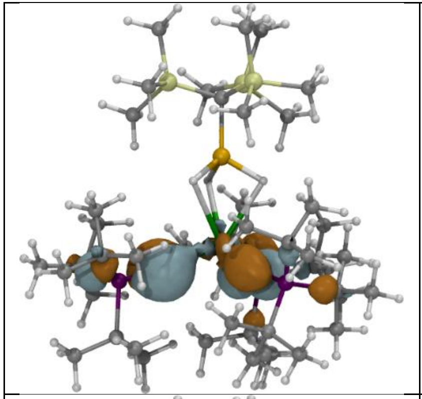	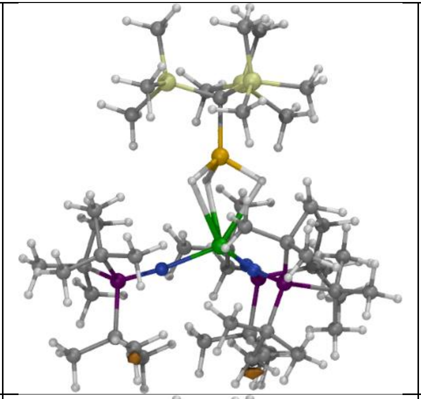	23.1%
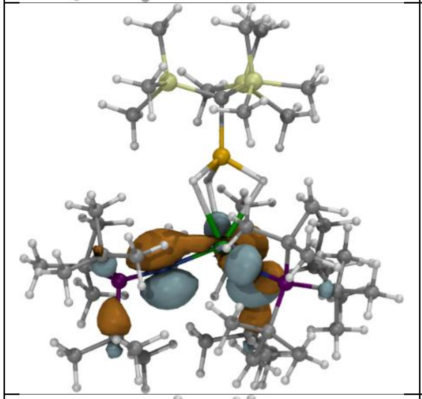	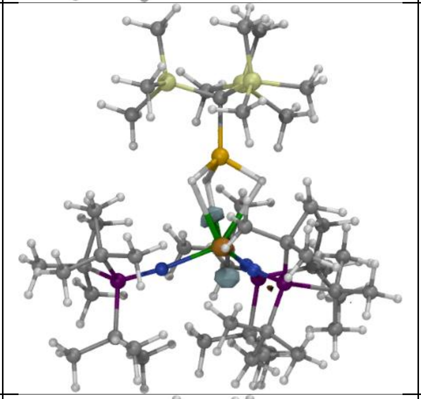	17.7%
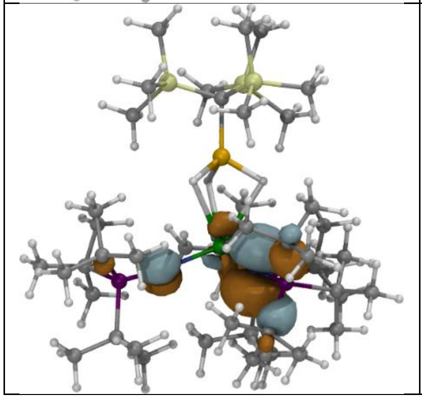	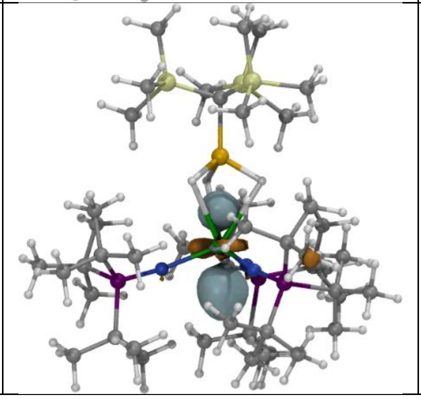	7.3%

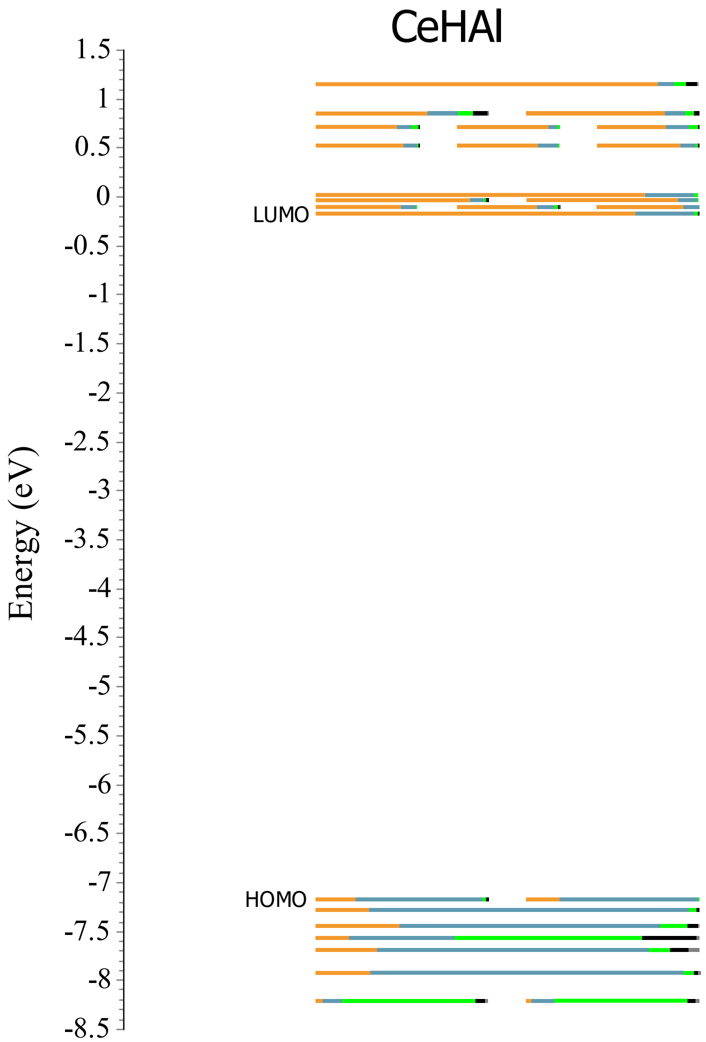

Figure S66. Molecular orbital energy diagram of **CeHAl** based on the CAM-B3LYP-D3 density. Fragments: Ce (orange), NP(^{t}Bu)$_3$ (turquoise), AlC(TMS)$_3$ (lime), κ^2-H (black), κ^1-H (gray).

References

(1) Gompa, T. P.; Rice, N. T.; Russo, D. R.; Aguirre Quintana, L. M.; Yik, B. J.; Bacsa, J.; La Pierre, H. S. Diethyl ether adducts of trivalent lanthanide iodides. *Dalton Trans.* **2019**, *48* (23), 8030-8033. DOI: 10.1039/C9DT00775J.

(2) Tateyama, H.; Boggiano, A. C.; Liao, C.; Otte, K. S.; Li, X.; La Pierre, H. S. Tetravalent Cerium Alkyl and Benzyl Complexes. *J. Am. Chem. Soc.* **2024**, *146* (15), 10268-10273. DOI: 10.1021/jacs.4c01964.

(3) Eaborn, C.; Gorrell, I. B.; Hitchcock, P. B.; Smith, J. D.; Tavakkoli, K. Preparation of New Lithium Organotrihydroaluminates: Crystal Structure of $\{(C_4H_8O)_2Li(\mu\text{-}H)_2AlH[C(SiMe_2Ph)_3]\}_2$. *Organometallics* **1994**, *13* (11), 4143-4144. DOI: 10.1021/om00023a007.

(4) Brown, A. C.; Altman, A. B.; Lohrey, T. D.; Hohloch, S.; Arnold, J. Hydride oxidation from a titanium–aluminum bimetallic complex: insertion, thermal and electrochemical reactivity. *Chem. Sci.* **2017**, *8* (7), 5153-5160. DOI: 10.1039/C7SC01835E.

(5) Evans, W. J.; Kozimor, S. A.; Ziller, J. W.; Kaltsoyannis, N. Structure, Reactivity, and Density Functional Theory Analysis of the Six-Electron Reductant, $[(C_5Me_5)_2U]_2(\mu\text{-}\eta^6\text{:}\eta^6\text{-}C_6H_6)$, Synthesized via a New Mode of $(C_5Me_5)_3M$ Reactivity. *J. Am. Chem. Soc.* **2004**, *126* (44), 14533-14547. DOI: 10.1021/ja0463886.

(6) Bergquist, C.; Bridgewater, B. M.; Harlan, C. J.; Norton, J. R.; Friesner, R. A.; Parkin, G. Aqua, Alcohol, and Acetonitrile Adducts of Tris(perfluorophenyl)borane: Evaluation of Brønsted Acidity and Ligand Lability with Experimental and Computational Methods. *J. Am. Chem. Soc.* **2000**, *122* (43), 10581-10590. DOI: 10.1021/ja001915g.

(7) Wilkerson, M. P.; Burns, C. J.; Dewey, H. J.; Martin, J. M.; Morris, D. E.; Paine, R. T.; Scott, B. L. Basicity of Uranyl Oxo Ligands upon Coordination of Alkoxides. *Inorg. Chem.* **2000**, *39* (23), 5277-5285. DOI: 10.1021/ic000142u.

(8) Brammer, L.; Connelly, N. G.; Edwin, J.; Geiger, W. E.; Orpen, A. G.; Sheridan, J. B. Structural consequences of electron transfer reactions. Pt. 17. One-electron oxidation of (N4-cot)RhCp: rearrangements of the radicalcation and of the resulting dimerization product. *Organometallics* **1988**, *7* (6), 1259-1265. DOI: 10.1021/om00096a004.

(9) Niklas, J. E.; Duffy, M. I.; La Pierre, H. S. A Guide to Nonaqueous Electrochemistry of f-Element Complexes. *Inorg. Chem.* **2026**, *65* (7), 3758-3770. DOI: 10.1021/acs.inorgchem.5c05041.

(10) Tateyama, H.; Liao, C.; Wilkinson, G. R.; Ramanathan, A.; Amidani, L.; Bazarkina, E.; Ressnik, F.; Engle, K. S.; Bacsa, J.; Sokaras, D.; et al. Single-Determinant Ground State in Ce^{4+} Imidophosphorane Complexes. *Inorg. Chem.* **2025**, *64* (50), 24401-24410. DOI: 10.1021/acs.inorgchem.5c03376.

(11) Boggiano, A. C.; Chowdhury, S. R.; Roy, M. D.; Bernbeck, M. G.; Greer, S. M.; Vlaisavljevich, B.; La Pierre, H. S. A Four-Coordinate Pr^{4+} Imidophosphorane Complex. *Angew. Chem. Int. Ed.* **2024**, *63* (43), e202409789. DOI: 10.1002/anie.202409789.

(12) *Bruker, SAINT, V8.40B, Bruker AXS Inc., Madison, Wisconsin, USA.*; (accessed 2026).

(13) Krause, L.; Herbst-Irmer, R.; Sheldrick, G. M.; Stalke, D. Comparison of silver and molybdenum microfocus X-ray sources for single-crystal structure determination. *J. Appl. Crystallogr.* **2015**, *48* (1), 3-10. DOI: doi:10.1107/S1600576714022985.

(14) Sheldrick, G. Crystal structure refinement with SHELXL. *Acta. Cryst. C* **2015**, *71* (1), 3-8. DOI: doi:10.1107/S2053229614024218.

(15) Sheldrick, G. SHELXT - Integrated space-group and crystal-structure determination. *Acta Cryst. A* **2015**, *71* (1), 3-8. DOI: doi:10.1107/S2053273314026370.
(16) *D. Kratzert, FinalCif, V155, https://dkratzert.de/finalcif.html.*; (accessed 2026).
(17) Mikeska, E. R.; Blakemore, J. D. Evidence for Reactivity of Decamethylcobaltocene with Dichloromethane. *Organometallics* **2023**, *42* (13), 1444-1447. DOI: 10.1021/acs.organomet.3c00176.
(18) Altman, A. B.; Brown, A. C.; Rao, G.; Lohrey, T. D.; Britt, R. D.; Maron, L.; Minasian, S. G.; Shuh, D. K.; Arnold, J. Chemical structure and bonding in a thorium(iii)–aluminum heterobimetallic complex. *Chem. Sci.* **2018**, *9* (18), 4317-4324. DOI: 10.1039/C8SC01260A.
(19) Mooney, J.; Kambhampati, P. Get the Basics Right: Jacobian Conversion of Wavelength and Energy Scales for Quantitative Analysis of Emission Spectra. *J. Phys. Chem. Lett.* **2013**, *4* (19), 3316-3318. DOI: 10.1021/jz401508t.
(20) Newville, M.; Otten, R.; Nelson, A.; Stensitzki, T.; Ingargiola, A.; Allan, D.; Fox, A.; Carter, F.; Michal; Osborn, R.; et al. lmfit/lmfit-py: 1.3.1. **2024**. DOI: 10.5281/zenodo.10998841.
(21) Rice, N. T.; Popov, I. A.; Russo, D. R.; Gompa, T. P.; Ramanathan, A.; Bacsa, J.; Batista, E. R.; Yang, P.; La Pierre, H. S. Comparison of tetravalent cerium and terbium ions in a conserved, homoleptic imidophosphorane ligand field. *Chem. Sci.* **2020**, *11* (24), 6149-6159. DOI: 10.1039/D0SC01414A.
(22) Rice, N. T.; Su, J.; Gompa, T. P.; Russo, D. R.; Telser, J.; Palatinus, L.; Bacsa, J.; Yang, P.; Batista, E. R.; La Pierre, H. S. Homoleptic Imidophosphorane Stabilization of Tetravalent Cerium. *Inorg. Chem.* **2019**, *58* (8), 5289-5304. DOI: 10.1021/acs.inorgchem.9b00368.
(23) Otte, K. S.; Niklas, J. E.; Studvick, C. M.; Boggiano, A. C.; Bacsa, J.; Popov, I. A.; La Pierre, H. S. Divergent Stabilities of Tetravalent Cerium, Uranium, and Neptunium Imidophosphorane Complexes. *Angew. Chem. Int. Ed.* **2023**, *62* (34), e202306580. DOI: 10.1002/anie.202306580.
(24) Franzke, Y. J.; Holzer, C.; Andersen, J. H.; Begušić, T.; Bruder, F.; Coriani, S.; Della Sala, F.; Fabiano, E.; Fedotov, D. A.; Fürst, S.; et al. TURBOMOLE: Today and Tomorrow. *J. Chem. Theory Comput.* **2023**, *19* (20), 6859-6890. DOI: 10.1021/acs.jctc.3c00347.
(25) *TURBOMOLE V7.8 2023, a development of University of Karlsruhe and Forschungszentrum Karlsruhe GmbH, 1989-2007, TURBOMOLE GmbH, since 2007; available from https://www.turbomole.org.*; (accessed 2026).
(26) Schäfer, A.; Horn, H.; Ahlrichs, R. Fully optimized contracted Gaussian basis sets for atoms Li to Kr. *J. Chem. Phys.* **1992**, *97* (4), 2571-2577. DOI: 10.1063/1.463096.
(27) Grimme, S.; Antony, J.; Ehrlich, S.; Krieg, H. A consistent and accurate ab initio parametrization of density functional dispersion correction (DFT-D) for the 94 elements H-Pu. *J. Chem. Phys.* **2010**, *132* (15). DOI: 10.1063/1.3382344.
(28) Treutler, O.; Ahlrichs, R. Efficient molecular numerical integration schemes. *J. Chem. Phys.* **1995**, *102* (1), 346-354. DOI: 10.1063/1.469408.
(29) Weigend, F.; Häser, M.; Patzelt, H.; Ahlrichs, R. RI-MP2: optimized auxiliary basis sets and demonstration of efficiency. *Chem. Phys. Lett.* **1998**, *294* (1), 143-152. DOI: 10.1016/S0009-2614(98)00862-8.
(30) Perdew, J. P.; Burke, K.; Ernzerhof, M. Generalized Gradient Approximation Made Simple. *Phys. Rev. Lett.* **1996**, *77* (18), 3865-3868. DOI: 10.1103/PhysRevLett.77.3865.
(31) Perdew, J. P.; Burke, K.; Ernzerhof, M. Generalized Gradient Approximation Made Simple [Phys. Rev. Lett. 77, 3865 (1996)]. *Phys. Rev. Lett.* **1997**, *78* (7), 1396-1396. DOI: 10.1103/PhysRevLett.78.1396.

(32) Perdew, J. P.; Ernzerhof, M.; Burke, K. Rationale for mixing exact exchange with density functional approximations. *J. Chem. Phys.* **1996**, *105* (22), 9982-9985. DOI: 10.1063/1.472933.
(33) Adamo, C.; Barone, V. Toward reliable density functional methods without adjustable parameters: The PBE0 model. *J. Chem. Phys.* **1999**, *110* (13), 6158-6170. DOI: 10.1063/1.478522.
(34) Krukau, A. V.; Vydrov, O. A.; Izmaylov, A. F.; Scuseria, G. E. Influence of the exchange screening parameter on the performance of screened hybrid functionals. *J. Chem. Phys.* **2006**, *125* (22). DOI: 10.1063/1.2404663.
(35) Becke, A. D. Density-functional thermochemistry. III. The role of exact exchange. *J. Chem. Phys.* **1993**, *98* (7), 5648-5652. DOI: 10.1063/1.464913.
(36) Yanai, T.; Tew, D. P.; Handy, N. C. A new hybrid exchange–correlation functional using the Coulomb-attenuating method (CAM-B3LYP). *Chem. Phys. Lett.* **2004**, *393* (1), 51-57. DOI: 10.1016/j.cplett.2004.06.011.
(37) Tao, J.; Perdew, J. P.; Staroverov, V. N.; Scuseria, G. E. Climbing the Density Functional Ladder: Nonempirical Meta--Generalized Gradient Approximation Designed for Molecules and Solids. *Phys. Rev. Lett.* **2003**, *91* (14), 146401. DOI: 10.1103/PhysRevLett.91.146401.
(38) Perdew, J. P.; Tao, J.; Staroverov, V. N.; Scuseria, G. E. Meta-generalized gradient approximation: Explanation of a realistic nonempirical density functional. *J. Chem. Phys.* **2004**, *120* (15), 6898-6911. DOI: 10.1063/1.1665298.
(39) Baerends, E. J.; Aguirre, N. F.; Austin, N. D.; Autschbach, J.; Bickelhaupt, F. M.; Bulo, R.; Cappelli, C.; van Duin, A. C. T.; Egidi, F.; Fonseca Guerra, C.; et al. The Amsterdam Modeling Suite. *J. Chem. Phys.* **2025**, *162* (16). DOI: 10.1063/5.0258496.
(40) Schreckenbach, G.; Ziegler, T. Calculation of NMR Shielding Tensors Using Gauge-Including Atomic Orbitals and Modern Density Functional Theory. *J. Phys. Chem.* **1995**, *99* (2), 606-611. DOI: 10.1021/j100002a024.
(41) Krykunov, M.; Ziegler, T.; Lenthe, E. v. Hybrid density functional calculations of nuclear magnetic shieldings using Slater-type orbitals and the zeroth-order regular approximation. *Int. J. Quantum Chem.* **2009**, *109* (8), 1676-1683. DOI: 10.1002/qua.21985.
(42) van Lenthe, E.; Ehlers, A.; Baerends, E.-J. Geometry optimizations in the zero order regular approximation for relativistic effects. *J. Chem. Phys.* **1999**, *110* (18), 8943-8953. DOI: 10.1063/1.478813.
(43) van Lenthe, E.; Baerends, E. J.; Snijders, J. G. Relativistic total energy using regular approximations. *J. Chem. Phys.* **1994**, *101* (11), 9783-9792. DOI: 10.1063/1.467943.
(44) Lenthe, E. v.; Baerends, E. J.; Snijders, J. G. Relativistic regular two-component Hamiltonians. *J. Chem. Phys.* **1993**, *99* (6), 4597-4610. DOI: 10.1063/1.466059.
(45) Van Lenthe, E.; Baerends, E. J. Optimized Slater-type basis sets for the elements 1–118. *J. Comput. Chem.* **2003**, *24* (9), 1142-1156. DOI: 10.1002/jcc.10255.
(46) Klamt, A.; Schüürmann, G. COSMO: a new approach to dielectric screening in solvents with explicit expressions for the screening energy and its gradient. *J. Chem. Soc., Perkin Trans. 2* **1993**, (5), 799-805. DOI: 10.1039/P29930000799.
(47) Autschbach, J.; King, H. F. Analyzing molecular static linear response properties with perturbed localized orbitals. *J. Chem. Phys.* **2010**, *133* (4). DOI: 10.1063/1.3455709.
(48) Autschbach, J.; Zheng, S. Analyzing Pt chemical shifts calculated from relativistic density functional theory using localized orbitals: The role of Pt 5d lone pairs. *Magn. Reson. Chem.* **2008**, *46* (S1), S45-S55. DOI: 10.1002/mrc.2289.

(49) Autschbach, J. Analyzing NMR shielding tensors calculated with two-component relativistic methods using spin-free localized molecular orbitals. *J. Chem. Phys.* **2008**, *128* (16). DOI: 10.1063/1.2905235.
(50) Rodríguez, J. I.; Bader, R. F. W.; Ayers, P. W.; Michel, C.; Götz, A. W.; Bo, C. A high performance grid-based algorithm for computing QTAIM properties. *Chem. Phys. Lett.* **2009**, *472* (1), 149-152. DOI: 10.1016/j.cplett.2009.02.081.
(51) Rodríguez, J. I. An efficient method for computing the QTAIM topology of a scalar field: The electron density case. *J. Comput. Chem.* **2013**, *34* (8), 681-686. DOI: 10.1002/jcc.23180.
(52) Ziegler, T.; Rauk, A. A theoretical study of the ethylene-metal bond in complexes between copper(1+), silver(1+), gold(1+), platinum(0) or platinum(2+) and ethylene, based on the Hartree-Fock-Slater transition-state method. *Inorg. Chem.* **1979**, *18* (6), 1558-1565. DOI: 10.1021/ic50196a034.
(53) Ziegler, T.; Rauk, A. Carbon monoxide, carbon monosulfide, molecular nitrogen, phosphorus trifluoride, and methyl isocyanide as sigma donors and pi acceptors. A theoretical study by the Hartree-Fock-Slater transition-state method. *Inorg. Chem.* **1979**, *18* (7), 1755-1759. DOI: 10.1021/ic50197a006.
(54) Bickelhaupt, F. M.; Baerends, E. J. Kohn-Sham Density Functional Theory: Predicting and Understanding Chemistry. In *Reviews in Computational Chemistry*, 2000; pp 1-86.
(55) Mitoraj, M. P.; Michalak, A.; Ziegler, T. A Combined Charge and Energy Decomposition Scheme for Bond Analysis. *J. Chem. Theory Comput.* **2009**, *5* (4), 962-975. DOI: 10.1021/ct800503d.
(56) Neese, F. The ORCA program system. *WIRES Comput. Mol. Sci.* **2012**, *2* (1), 73-78. DOI: 10.1002/wcms.81.
(57) Neese, F. Software Update: The ORCA Program System—Version 6.0. *WIRES Comput. Mol. Sci.* **2025**, *15* (2), e70019. DOI: 10.1002/wcms.70019.
(58) Pantazis, D. A.; Chen, X.-Y.; Landis, C. R.; Neese, F. All-Electron Scalar Relativistic Basis Sets for Third-Row Transition Metal Atoms. *J. Chem. Theory Comput.* **2008**, *4* (6), 908-919. DOI: 10.1021/ct800047t.
(59) Rolfes, J. D.; Neese, F.; Pantazis, D. A. All-electron scalar relativistic basis sets for the elements Rb–Xe. *J. Comput. Chem.* **2020**, *41* (20), 1842-1849. DOI: 10.1002/jcc.26355.
(60) Weigend, F.; Ahlrichs, R. Balanced basis sets of split valence, triple zeta valence and quadruple zeta valence quality for H to Rn: Design and assessment of accuracy. *Phys. Chem. Chem. Phys.* **2005**, *7* (18), 3297-3305. DOI: 10.1039/B508541A.
(61) Izsák, R.; Hansen, A.; Neese, F. The resolution of identity and chain of spheres approximations for the LPNO-CCSD singles Fock term. *Mol. Phys.* **2012**, *110* (19-20), 2413-2417. DOI: 10.1080/00268976.2012.687466.
(62) Izsák, R.; Neese, F. An overlap fitted chain of spheres exchange method. *J. Chem. Phys.* **2011**, *135* (14). DOI: 10.1063/1.3646921.
(63) Izsák, R.; Neese, F.; Klopper, W. Robust fitting techniques in the chain of spheres approximation to the Fock exchange: The role of the complementary space. *J. Chem. Phys.* **2013**, *139* (9). DOI: 10.1063/1.4819264.
